# Identification of novel genes involved in phosphate accumulation in *Lotus japonicus* through Genome Wide Association mapping of root system architecture and anion content

**DOI:** 10.1371/journal.pgen.1008126

**Published:** 2019-12-19

**Authors:** Marco Giovannetti, Christian Göschl, Christof Dietzen, Stig U. Andersen, Stanislav Kopriva, Wolfgang Busch

**Affiliations:** 1 Gregor Mendel Institute (GMI), Austrian Academy of Sciences, Vienna Biocenter (VBC), Vienna, Austria; 2 University of Cologne, Botanical Institute and Cluster of Excellence on Plant Sciences (CEPLAS), Cologne, Germany; 3 Department of Molecular Biology and Genetics, Aarhus University, Denmark; 4 Salk Institute for Biological Studies, Plant Molecular and Cellular Biology Laboratory, and Integrative Biology Laboratory, La Jolla, California, United States of America; Technical University of Munich, GERMANY

## Abstract

Phosphate represents a major limiting factor for plant productivity. Plants have evolved different solutions to adapt to phosphate limitation ranging from a profound tuning of their root system architecture and metabolic profile to the evolution of widespread mutualistic interactions. Here we elucidated plant responses and their genetic basis to different phosphate levels in a plant species that is widely used as a model for AM symbiosis: *Lotus japonicus*. Rather than focussing on a single model strain, we measured root growth and anion content in response to different levels of phosphate in 130 Lotus natural accessions. This allowed us not only to uncover common as well as divergent responses within this species, but also enabled Genome Wide Association Studies by which we identified new genes regulating phosphate homeostasis in Lotus. Among them, we showed that insertional mutants of a cytochrome B5 reductase and a Leucine-Rich-Repeat receptor showed different phosphate concentration in plants grown under phosphate sufficient condition. Under low phosphate conditions, we found a correlation between plant biomass and the decrease of plant phosphate concentration in plant tissues, representing a dilution effect. Altogether our data of the genetic and phenotypic variation within a species capable of AM complements studies that have been conducted in Arabidopsis, and advances our understanding of the continuum of genotype by phosphate level interaction existing throughout dicot plants.

## Author summary

Phosphate is one of the main limiting factors for plant growth. Plants have evolved different strategies to face phosphate limitation ranging from a profound tuning of their root system architecture and metabolic profile to the evolution of widespread mutualistic interactions, such as arbuscular mycorrhizal symbiosis. Here we investigate plant responses and their genetic basis to different phosphate levels in a model legume plant species, *Lotus japonicus*, a plant commonly used for studying root endosymbioses. We combine high throughput root phenotyping and nutrient measurements with a natural variation approach. With this approach, we elucidate relations between root phenotypic responses and nutrient accumulation and we uncover, under low phosphate conditions, a correlation between plant biomass and the decrease of plant phosphate concentration in plant tissues. Using Genome Wide Association mapping and integration of multiple traits, we identified new genes regulating phosphate homeostasis in Lotus and confirmed their functions using multiple independent insertion mutant lines.

## Introduction

Phosphate is an essential element for plant growth and its bioavailability represents a major limiting factor for plant productivity. Plants coping with phosphate deficiency exhibit dramatic changes at the developmental, nutritional and metabolic levels. For example, in *Arabidopsis thaliana*, the root developmental program has been described to be highly affected by phosphate deficiency. Primary root growth of the reference accession Col-0 is inhibited and there is an increase of lateral root formation and root hair growth [1]. The key genetic determinants of this process have been identified, mainly through forward genetic screening [2,3]. Recently, it has been shown that a main driver of primary root growth arrest is toxicity of iron that, upon phosphate starvation, accumulates in the meristematic zone and induces a progressive loss in the proliferative capacity of the cells, causing reduction in meristem length [4]. Beside this strong local effect at the root tip, phosphate deficiency has also a dramatic systemic effect causing a general remodeling of main cellular processes, mainly orchestrated by a transcriptomic cascade orchestrated by the interaction between PHR1 [5,6] and its SPX interactors [7–9]. In addition to that, phosphate-depleted plants usually display a high turnover of phospholipids into galactolipids and sulfolipids [10]. Furthermore, there is a substantial interaction of phosphate related processes and other environmental factors. For instance, red light frequencies lead to an increase of phosphate uptake and Arabidopsis accessions with light-sensing defects, such as Lm-2 and CSHL-5, take up less phosphate [11]. Moreover, plant responses to phosphate are altered by the abundance of metal ions, such as iron and zinc, with the extent of the impact being dependent on the genetic background [12,13]. Taken together, responses to phosphate and phosphate homeostasis in plants are regulated in a complex manner and are substantially dependent on plant genetic diversity and environmental abiotic and biotic factors.

For biotic factors, it was recently shown in Arabidopsis and related plants how fungi and bacteria play a key role in mediating phosphate accumulation [14,15]. Molecular hubs of phosphate metabolism, such as PHR1, can define the composition of microbial communities [16] and the analysis of microbial contribution to plant phosphate accumulation can even be mathematically modeled, giving rise to the possibility to design ad-hoc microbial communities with desired effects on plant phosphate uptake [17]. The most prominent example of microorganisms providing plants with phosphate is the symbiosis of plants with arbuscular mycorrhizal (AM) fungi. A key aspect of this is that fungal hyphae are very efficient in exploring the soil and taking up the highly immobile phosphate. Consequently, up to 80% of the needed phosphate can be acquired and transferred to plants by these fungi [18–21]. Numerous plant genetic determinants that are needed for the establishment of a functional mycorrhizal symbiosis have been described over the last 20 years but a lack of high-throughput methods and the complexity of the system impaired the comprehensive understanding of the crucial and complex feedback happening between plant phosphate status and the establishment of AM symbiosis [22].

Most genetic screens aiming for identifying genes that regulate plant responses to phosphate have been conducted in species incapable of AM symbiosis such as Arabidopsis. In this study we targeted the genetic basis of responses to phosphate levels in a plant widely used as a model for endosymbioses, as a first step for establishing tools allowing to use *Lotus japonicus* as a model for plant phosphate nutrition. To do so, we conducted large-scale studies of *Lotus japonicus* natural variation of root responses to different levels of phosphate, coupled with the measurement of anion accumulation. We discovered profound correlations of plant size and plant phosphate concentration that should be taken into account when working with concentration measures. Using high-density SNP data from the 130 Lotus accessions, we conducted Genome Wide Association Studies (GWAS) for all measured traits, finding hundreds of genetic loci associated with variation in phosphate-related traits. Finally, by comparing the lists of candidate genes for root system architecture and phosphate accumulation, we identified a Leucine-Rich Receptor kinase and a cytochrome B5 reductase involved in phosphate homeostasis as high confidence causal genes, which was further corroborated by phosphate dependent phenotypes of loss of function mutants for these genes.

## Results

### Phosphate deficiency shapes natural variation of root growth and anion levels in roots and shoots of *Lotus japonicus*

**Fig 1:**
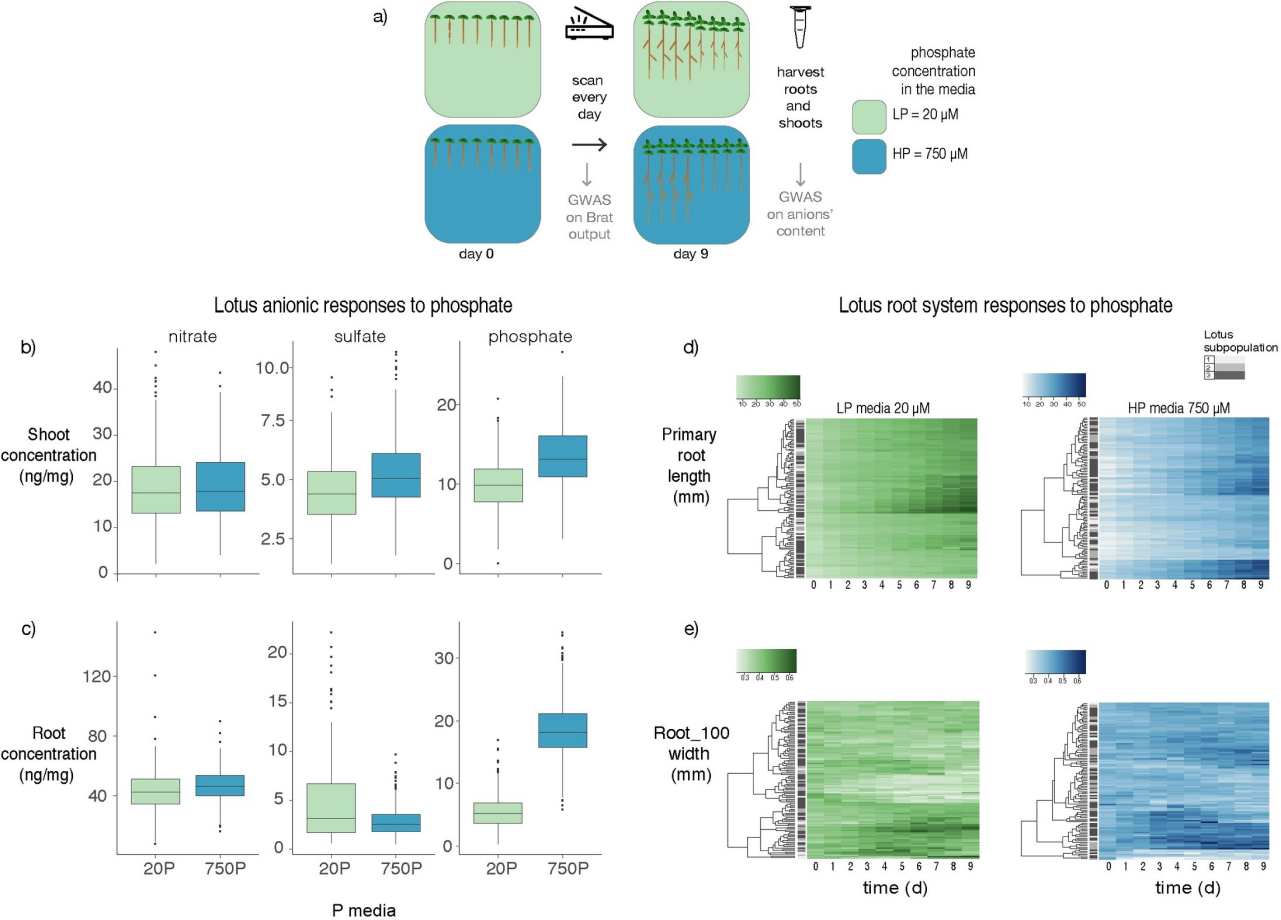
*Lotus japonicus* natural variation of root responses to phosphate media levels over time. a) Set up of the experiment. One-hundred and thirty Lotus accessions were grown on low (20 μM) or high (750 μM) phosphate media for 9 days. Plates were scanned every day, and root traits were quantified and segmented using BRAT. After 9 days, shoots and roots were harvested and nitrate, phosphate and sulfate concentration were measured. All traits were then used for running GWAS. Here, we show representative traits of anions’ accumulation and primary root traits of the Lotus panel. b-c) Concentration of anions in roots and shoots depends on the phosphate concentration in the media. Both shoot and root accumulation of anions show a significant effect in response to phosphate concentration in the media. A strong effect is shown for phosphate content in roots and shoots and sulfate content in shoots. d-e) Over a 9-day time course, Lotus natural accessions show a high diversity in root growth over LP and HP, both for primary root length and root width. Clustering of responses to nutrient is not dependent on Lotus subpopulation origin.

To study the genetic bases of root responses to low phosphate and phosphate accumulation in *Lotus japonicus* (Lotus) tissues, we performed a detailed root phenotyping of a panel of 130 diverse Lotus natural accessions [23] over a 9-day time course and subsequently quantified anions of the main macronutrients from the same material (Fig 1A). In particular, we grew the plants on vertical plates for 9 days on a modified Long-Ashton medium (S1 Table), containing either 20 μM (LP) or 750 μM (HP) of phosphate. We scanned the plates daily, at the same time of the day. At the end of the 9th day, we harvested and weighed total root and total shoot (stem and leaves) material for subsequent quantification of main macronutrients: nitrate, phosphate and sulfate.

**Fig 2:**
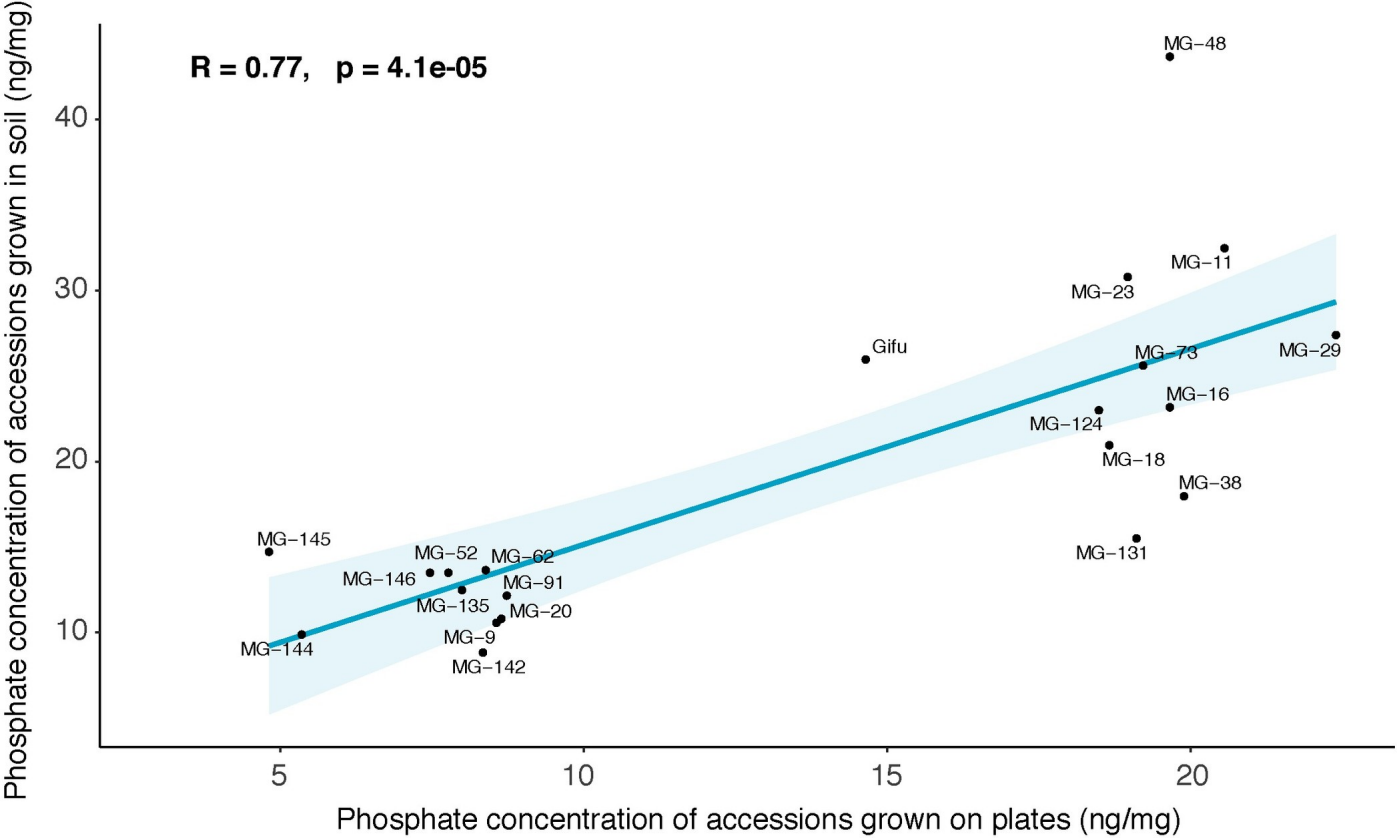
Correlation between phosphate concentration in plants grown on agar plates and in soil. Each dot represents median concentration of phosphate in *Lotus japonicus* accessions. The value results from 4 biological replicates. In the top left corner, Pearson’s coefficient and p value are indicated. Colored lined and colored shades represent linear regression and 95% confidence intervals. Both agar plates and soil contained non limiting concentration of phosphate: agar plates contained 750 μM of phosphate and soil contained 200 mg/L of phosphate.

As shown in Fig 1B and 1C, wide variation among macronutrient concentrations is observed in different accessions. Phosphate levels in the medium not only affect plant phosphate concentration in roots and shoots, but also plant sulfate levels and, to a minor extent, nitrate concentration in roots, exposing a similar cross-talk between the three anions in Lotus as the one that had been described in Arabidopsis [24]. With the exception of phosphate concentration of plants grown under phosphate starvation, the concentration of these anions did neither depend on plant size nor on plant developmental stage (S1 Fig). As agar plates are a highly artificial environment for plant growth, we wanted to test whether similar differences of anion levels would also occur in soil grown accessions. We therefore selected 20 Lotus accessions representing the highest and lowest phosphate accumulation respectively and grew them in soil filled pots. Importantly, phosphate concentrations were strongly positively and significantly correlated among the two growth systems (Fig 2) as were total phosphorus and soluble phosphate (S2 Fig).

By using a modified version of the Brat Fiji plugin [25,26], we quantified 16 root traits per each day (8 traits representing direct measurements from the plant image and 8 traits that are mathematically derived from these direct measurements; S14 Table). These root traits showed a broad spectrum of responses among accessions (Fig 1D and 1E). There was a pronounced effect of phosphate on the majority of traits and interactions of effects between genotype and phosphate (S1 File). We then explored whether the response to phosphate levels merely reflects the genetic relation between Lotus natural accessions. For this we conducted hierarchical clustering of root growth and root tip width in both phosphate levels and found that the clusters do not reflect the established genetic Lotus subpopulation structure [23] but rather depend on phosphate level in the medium (Fig 1D and 1E).

In contrast to the early root growth responses described in the Arabidopsis reference accession, low phosphate medium does not induce a dramatic arrest of primary root growth in most of the Lotus accessions (S3A Fig). Similar PR responses have also been also observed in non-reference Arabidopsis accessions [27], the diversity of root growth responses to phosphate levels in Lotus seems to resemble that in Arabidopsis. Beyond total root length, root traits related to the root diameter, such as root width, are substantially influenced by phosphate deficiency: the width of the first 20% adjacent to the root/shoot junction (denominated as trait “Root width 20”) and the distal 20% (“Root width 100”), do get larger over time in plants grown in HP (S3B and S3C Fig).

**Fig 3:**
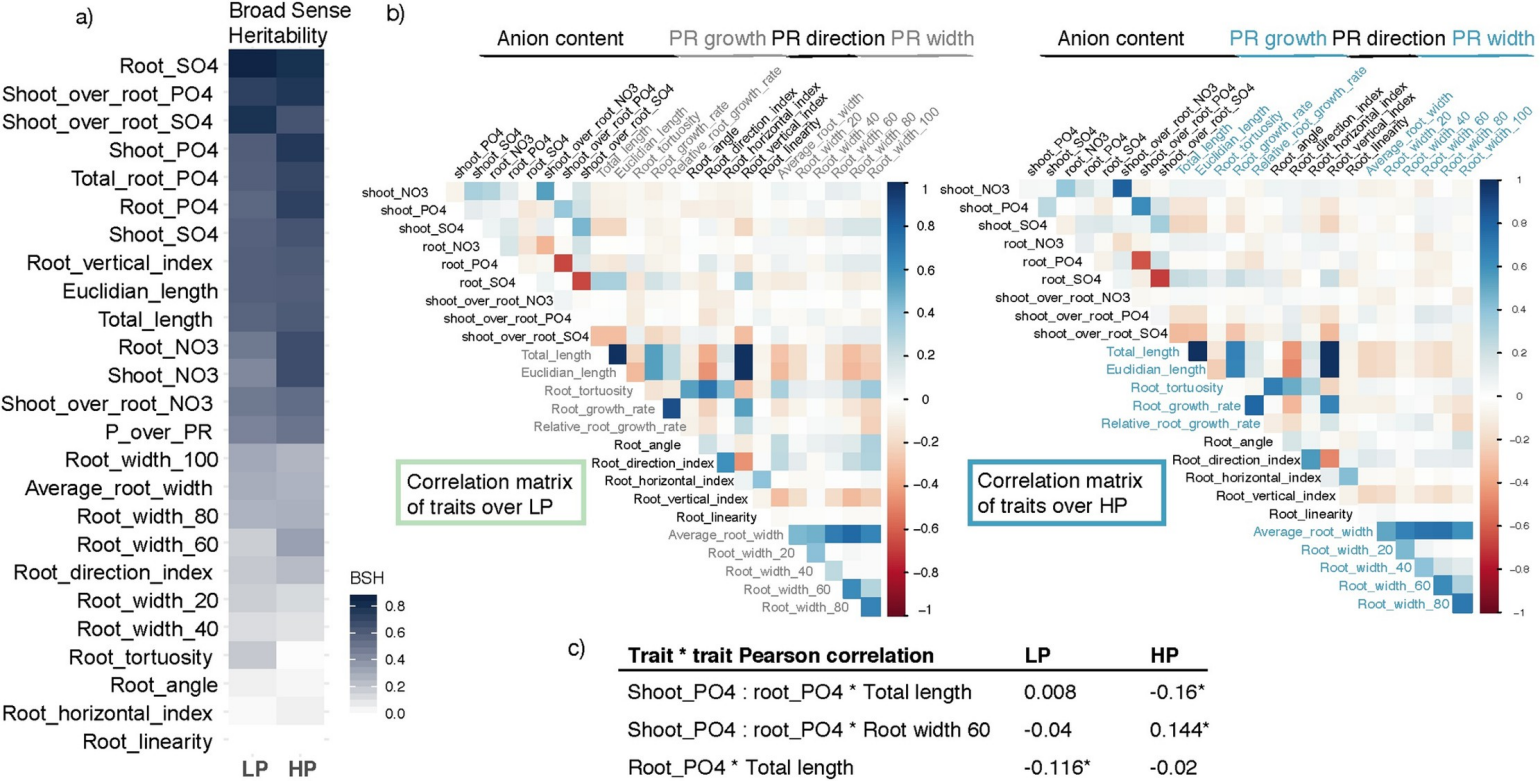
Pattern of Lotus correlation among root and anion traits and broad sense heritability at day 9 under LP or HP. a) Broad Sense Heritability of all measured traits on low and high phosphate. Highest heritability is shown by traits related to nutrient accumulation. Among root system architecture traits, those related to primary root length show higher broad sense heritability. b) Heatmap of Pearson’s pairwise correlations on every measured trait (root system architecture and nutrient accumulation) on day 9. On a population level, the root growth traits show distinct and recursive features: root width from the different root parts are all positively correlated. A similar pattern is shown also by traits related to primary root growth, such as total length, Euclidian length, root vertical index. By contrast root width and length parameters are negatively correlated: the longer the root, the thinner it gets. Correlation between root system architecture traits and tissue anion concentrations show the same pattern in LP and HP with a few exceptions. c) Total length and shootPO4:rootPO4 do show an opposite behavior among the two conditions. In particular in low phosphate plates, primary root length is negatively correlated with root phosphate concentration in discordance with standard phosphate condition. By contrast, shootPO4:rootPO4 displays a clear opposite pattern. Pearson’s r values are indicated and the asterisk represents p-value < 0.05.

The broad sense heritability (BSH) was different among the traits that we measured. While the variation of some traits could not be explained by genetics (Root linearity and root angle, ~0%), most traits are genetically determined and some to an extraordinarily high degree (root_SO4, 86%). Generally, traits related to nutrient accumulation showed higher heritability (Fig 3A). Among the root developmental traits, total root length showed the highest BSH (~60%), consistently with data from other root system architecture studies in *Arabidopsis thaliana* [28].

Taken together, we found that Lotus natural accessions exhibit a great variation of responses to phosphate concentrations, both at the phenotypic and at anion level. Moreover, the similar profound extent of natural variation of root growth between Arabidopsis and Lotus suggests that a large genotype by phosphate level interaction exist within species throughout the dicot group and regardless of whether a species is capable to form AM symbiosis.

### There is a trade-off of root phosphate concentration and root length specifically under low phosphate conditions

Phosphate starvation has a profound impact on root development and nutrient accumulation. Nevertheless, the link of the two processes remains largely unknown. Since we quantified phosphate, sulfate and nitrate concentrations from roots and shoots of single plants, and also measured root traits over time, we were able to compare trait correlations in LP or HP. This represents a valuable dataset to investigate the links between plant developmental adaptations and cellular metabolic tuning. For this, we calculated the pairwise Pearson's correlation coefficients of all root trait data and anion content in high and low phosphate from all Lotus accessions (Fig 3B). The contrasting phosphate levels in the two media did not perturb the majority of correlations among traits (Fig 3B): for example root width at different sectors along the root were highly positively correlated regardless of phosphate level, as well as the traits related to root length (Euclidian length, root growth rate, total length and relative root growth rate). Root length was negatively correlated with root width, in both conditions, indicating that longer roots are usually thinner in our working conditions.

Nevertheless, we could observe several peculiar correlations occurring exclusively in one of the two conditions: first, the ratio of phosphate concentration between shoot and root, that describes how much of the uptaken phosphate is transferred to the shoot, is significantly negatively correlated with root length and positively correlated with root width under HP but not LP (Fig 3C). This indicates that under HP less phosphate is allocated to the shoot when roots are elongating. However, when phosphate becomes limiting in the medium (LP), the total length of roots and root phosphate concentration are moderately negatively correlated (Fig 3B and S4 Fig). This suggests that available phosphate is distributed over a larger amount of root tissue if roots are longer and is thereby diluted. Dilution effect due to a change of nutrient availability, or different developmental stage, has been widely described [29]. Dilution also explains the negative correlation between plant biomass and phosphate concentration (S1 Fig). By contrast, and in agreement with this dilution model, the negative correlation of phosphate levels and root length is completely absent in HP media (Fig 3C) and much reduced when considering plant biomass and phosphate concentration (S1 Fig).

Altogether a parallel quantification of phosphate accumulation and root system architecture of a panel of 130 Lotus grown at two different phosphate concentrations allowed for the detection of trait correlation structure, showing that most of trait-correlation are not perturbed by phosphate levels. Our analysis also revealed that, in our experiment, the majority of nutrient accumulation traits show higher broad sense heritability compared to root developmental traits.

### GWAS for Lotus phosphate related traits identifies hundreds of unknown and known candidate genes for phosphate homeostasis

Given the extensive natural variation of most traits in the Lotus panel, we conducted a Genome Wide Association Study (GWAS) for each trait and each time point in LP and HP condition, using a mixed model algorithm, corrected for population structure [30–32] and using sequencing-based SNPs for the Lotus accessions (Shah et al., 2018). Each trait led to the identification of genetic loci significantly associated with variation of those traits: using a Benjamini-Hochberg FDR threshold of 5%, we found 900 SNPs associated with root growth parameters of plants grown on LP media (S3 Table), 939 SNPs with HP conditions (S4 Table), 3673 of LP/HP root growth ratio (S2 Table), 104 associated with phosphate content and 220 with anion content (S5 and S13 Tables).

Several obvious candidate genes were in these lists. For genes associated with morphological root traits, our analysis identified homologues of several known regulators of root development, such as an homologous gene of SCARECROW [33], Lj3g3v0821320, associated with Root horizontal index at day 6 under low phosphate conditions. Similarly, sequence variation in the genomic region of a Lotus BIG BROTHER homologue [34], Lj3g3v0489450, is associated with relative root growth rate over day 1–2. Another candidate gene identified as a potential regulator of Lotus root responses to low phosphate is the homologous gene of STOP1 (Lj0g3v0231229) that is associated with Root width 20 variation at day 3. In Arabidopsis, STOP1 was recently described as a key regulator of early root responses to phosphate deficiency-induced iron toxicity [35,36], therefore a similar role is conceivable for Lotus. Various fatty acyl-CoA reductases are highly associated with root width 80 day 2 and have been shown to be involved in alcohol synthesis in response to various stresses leading to suberin accumulation [37].

In parallel, the quantification of phosphate concentration at root and shoot level for the Lotus natural accessions grown at two different levels of phosphate led to the identification of several genetic loci associated with variations in those traits. Among the genes associated with phosphate concentration-related traits, we identified a trehalose-phosphate phosphatase-like protein (Lj4g3v2820240) associated with shoot_[PO4]_:root_[PO4]_ in LP. A significant association was also detected for a SNP within a UDP-glucuronic acid decarboxylase gene (Lj4g3v2312430), associated with shoot phosphate concentration in HP plants. Variation in the genetic region spanning a candidate sugar/phosphate translocator (Lj1g3v4830440) is correlated with root phosphate concentration in HP, as well as a UDP pyrophosphate phosphatase (Lj0g3v0276539) associated with shoot phosphate concentration in HP. Altogether many genes related to phosphate recycling seem to be linked with phosphate accumulation in shoots and roots in both media conditions, even though GO enrichment analysis did not highlight any obvious category (S11 and S12 Tables).

### Overlap among Lotus GWAS from different traits exposes loci associated with both Lotus root growth upon phosphate starvation and phosphate accumulation

**Fig 4:**
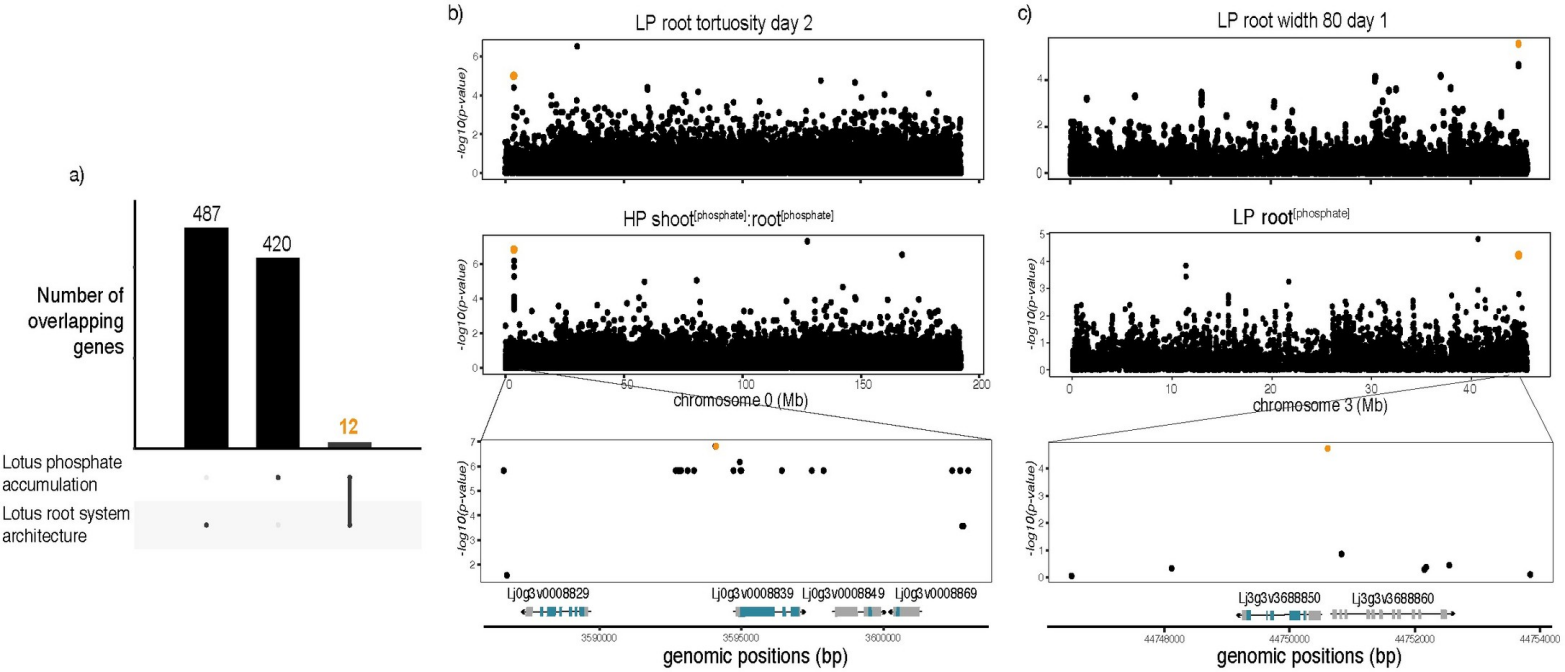
Overlap between GWAS hits from root system architecture and phosphate accumulation. a) The intersection of candidate genes associated with changes in root system architecture (487) and phosphate accumulation traits (420) is 12, corresponding to 7 genomic regions. B) Manhattan plots of two traits leading to the identification of one genomic region on chromosome 0. A close-up on that region shows a 20kb region containing two protein-coding genes and two non-coding genes. c) Manhattan plots of the two traits leading to the identification of a genomic region on chromosome 3. A close-up on a 20kb region containing one single protein-coding genes, a cytochrome B5-reductase.

Beyond investigating specific genetic associations between Lotus SNPs and particular root traits or phosphate accumulation values, one of our main interests within this study was to use both our nutrient and root growth data to specifically determine genes that control Lotus responses to phosphate. To accomplish that, we considered all genes in 10 kb genomic regions (Linkage Disequilibrium decays in Lotus, r^2^<0.2, with 10 kb [23]) centered on the SNPs passing our GWAS detection threshold. Because it was our purpose to assess overlaps, we took a non-conservative threshold for this approach. We considered up to 500 SNPs with a Benjamini-Hochberg threshold of 10^−5^ for the two groups of traits: phosphate content and root system architecture. This approach led to an overlap consisting of only 7 genomic regions (S8 Table) that were associated with both root and phosphate accumulation traits. These regions were in close proximity to 12 genes (Fig 4A, Table 1).

**Table 1:** List of overlapping genes between GWAS on phosphate content and root growth responses traits.

Overlapping genes	Description
Lj0g3v0008839	LRR receptor-like serine/threonine-protein kinase
Lj0g3v0008849	Non-coding gene
Lj2g3v0561100	Nodulation receptor kinase
Lj2g3v0561110	Plastid DNA-binding protein
Lj2g3v0636990	Non-coding gene
Lj2g3v1020000	Senescence-associated carboxylesterase 101
Lj2g3v3261180	DNA double-strand break repair Rad50 ATPase-like isoform X1
Lj2g3v3261190	DNA double-strand break repair Rad50 ATPase-like isoform X2
Lj2g3v3261200	Non-coding gene
Lj2g3v3261210	octicosapeptide/Phox/Bem1p domain-containing protein kinase
Lj3g3v3688850	cytochrome b5 reductase 4-like
Lj3g3v3688860	Non-coding gene

Among those regions, we specifically focused on genes coding for proteins and expressed in roots. As a first step, we focused on two of these regions, each encompassing a single protein coding gene that was expressed in roots (according to publicly available expression data [38]) and had possible functions related to signaling and/or acquisition of phosphate. One locus was associated with shoot_[PO4]_:root_[PO4]_ on HP and root tortuosity at day 2 on LP (Fig 4B, S5 Fig) and the other locus was associated with root phosphate concentration on LP and root width 80 at day 1 on LP (Fig 4C, S6 Fig). Interestingly, this locus was also associated with LP:HP root growth rate between day 7 and day 8 (S2 Table, S6 Fig).

**Fig 5:**
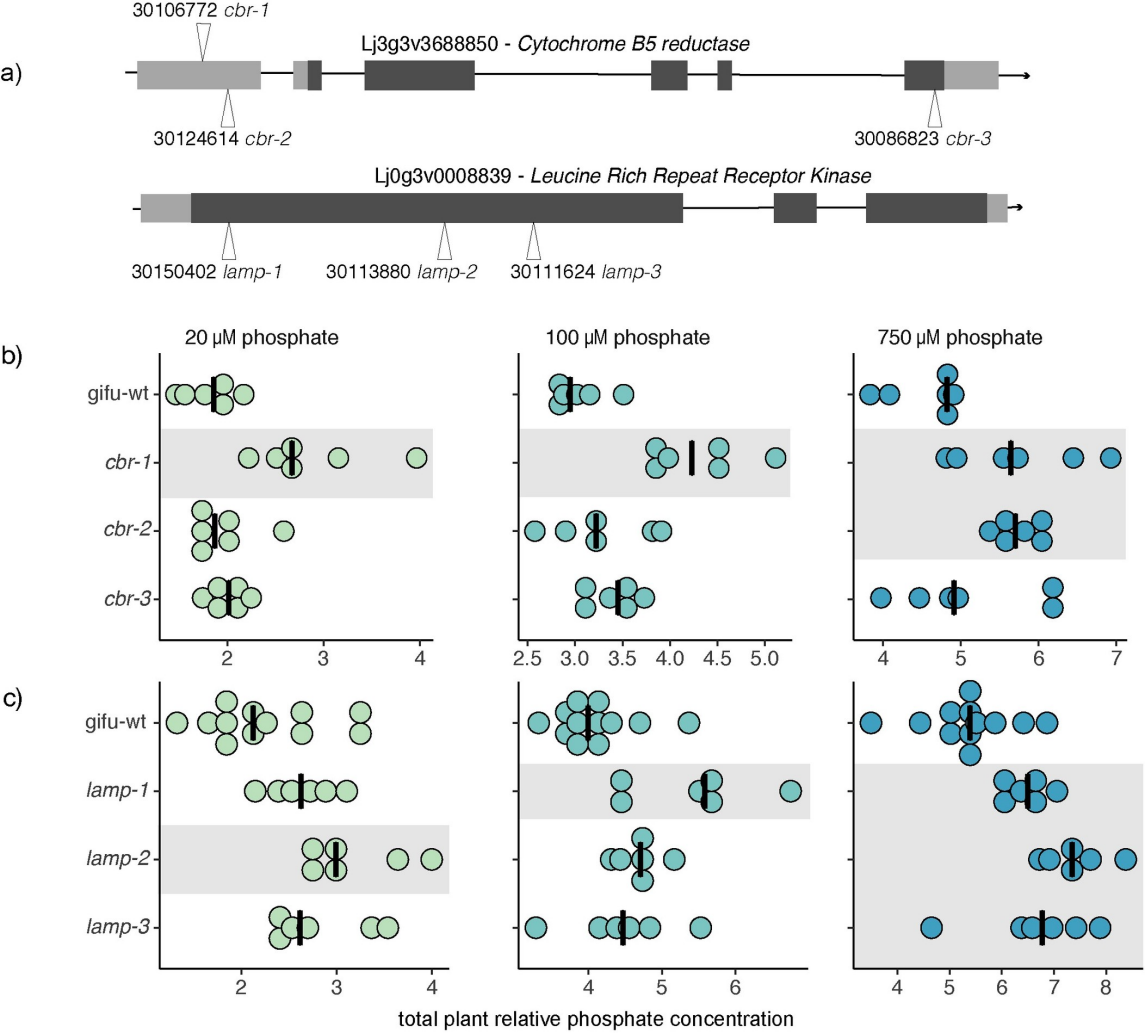
*cbr* and *lamp* mutants accumulate more phosphate than wt in high phosphate media. a) Gene structure and insertion mutants used in this study for *CBR* (*CYTOCHROME B5 REDUCTASE*) and *LAMP* (*LRR ACCUMULATING MORE PHOSPHATE*). Each number represents the Plant ID from Lotus Base. Three insertion mutants per each gene were used. b) Plant phosphate concentration levels of wt and LORE1 *cbr* insertion mutant plants growing under low (20 μM) or mid (100 μM) or high phosphate level (750 μM). Whereas at low and medium concentrations, only *cbr-1* show a significant difference compared to wt plants, at high phosphate concentration also *cbr-2* is accumulating significantly more phosphate. c) Total phosphate concentration of *lamp* mutant plants and wt in the three phosphate media conditions. All three *lamp* LORE1 insertion lines show a higher phosphate accumulation compared to wt. Each dot represents a single plant and black vertical lines represent the median among the group. Levels of phosphate are expressed relative to wt root plants at 20 μM. Different shades represent different groups compared to wt, following ANOVA test on estimated marginal means (Tukey’s adjusted p-value < 0.05).

**Fig 6:**
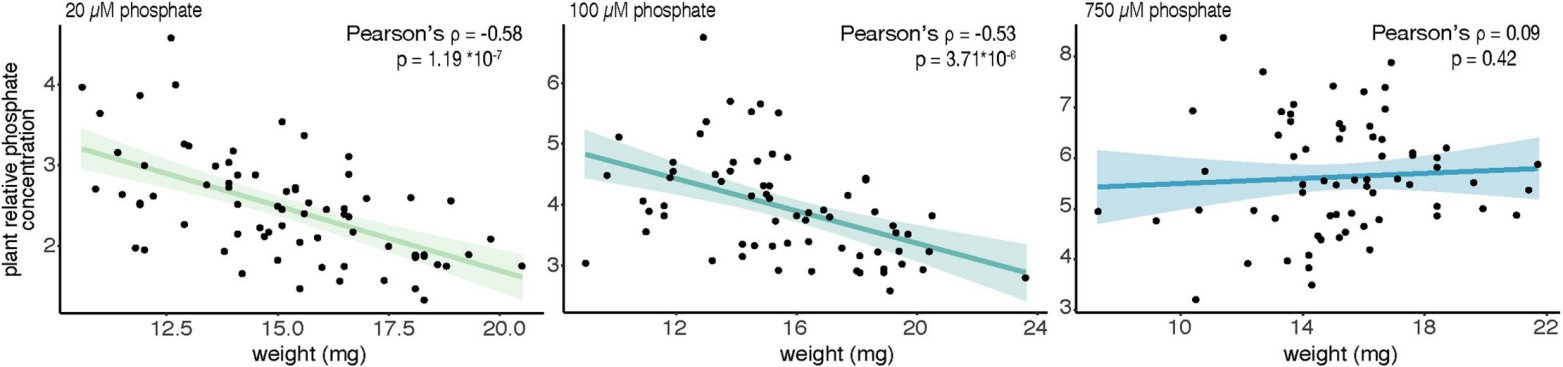
Plant phosphate concentration is negatively correlated with plant biomass when phosphate is the limiting factor. Phosphate concentrations of plants growing under low (20 μm) or medium (100 μM) phosphate are highly negatively correlated with plant biomass (Pearson’s correlation is -0.58 and -0.53 respectively and p-value <10^−6^). By contrast, under high phosphate level (750 μM), no significant correlation is observed between plant biomass and plant phosphate concentration. Each dot represents a single plant from different experiments. Phosphate concentration is calculated relative to wt roots phosphate concentration on 20 μM phosphate. Colored lines and colored shades represent linear regression and 95% confidence intervals respectively.

In both cases, the above mentioned SNPs span a 10kb LD region with one protein-coding gene that is expressed in roots: Lj0g3v0008839, coding for a LRR receptor-like serine/-rich (LRR-RK) protein on chromosome 0 and Lj3g3v3688850, a putative cytochrome b5 reductase (CBR) (S15–S19 Table). To test whether these candidate genes have a role in controlling responses to phosphate level, we selected homozygous plants (S7 Fig) from multiple insertional mutants for each gene from Lotus Base [38,39] as represented in Fig 5A. Two of the LORE1 insertion lines (line 30106772 and line 30124614, further denominated as *cbr-1* and *cbr-2* respectively) harbor insertions in the 5’UTR of the gene, whereas the line 30086823 (*cbr-3*) carries an insertion that is located at the far end of the last exon of the gene. All three LORE1 lines for the LRR-RK carry insertions in the first exon. We quantified the tissue phosphate concentration in homozygous mutant plants for both genes in three different phosphate concentrations (20, 100 and 750 μM), 10 days after transfer to specific media plates (Fig 5B and 5C). All three independent mutant lines of the LRR-RK showed an increased total plant phosphate concentration on high phosphate concentrations (Fig 5B and 5C and S7 Table). Accordingly, we named the gene *LAMP* (**L**RR-RK **A**ccumulating **M**ore **P**hosphate). Two out of three *cbr* mutant lines showed an increased total plant phosphate concentration on high phosphate concentrations, specifically driven by shoot phosphate levels (S8 Fig). The WT-like responding mutant line was different in the transposon insertion site with respect to the other two mutant lines and still might have some remaining activity of the protein due to its transposon insertion being at the far end of the gene (Fig 5A). While the observable effects of the loss of function of these two genes was significant in HP, a diverse and variable phosphate accumulation took place in LP conditions. We reasoned that the effect of LP on biomass and root length that we had observed earlier (S1 Fig) might confound the effects on phosphate content. Therefore, we assessed these correlations also across the wt and LORE1 insertion lines. As observed within the large panel of accessions, we found that while total phosphate concentration from mutant and wt plants grown under strong or mild phosphate starvation (20, and 100 μM) was strongly negatively correlated with plant biomass (ρ = -0.58, ρ = -0.55, respectively) (Fig 6), this correlation was completely absent under HP (750 μM). Interestingly the stronger correlations are more pronounced considering the whole plant compared to root or shoot separately (S9 Fig). To further test whether LORE1 insertions in the genes and not background mutations were responsible for the observed phenotypes in phosphate accumulation, we analyzed segregating progeny from the analyzed LORE1 lines. Consistent with the causality of the LORE1 insertions in the target genes, we found that plants containing the mutant alleles have higher phosphate concentration than those with wt alleles, which is not due to differences in plant biomass (S10 Fig). In a similar way, also the LORE1 insertional lines did not show any consistent plant growth difference compared to wt (S11 Fig), thereby demonstrating that the effects on phosphate concentration are not due to different plant growth.

While we selected these two candidate genes for being associated with phosphate level dependent phosphate content as well as root growth traits, we could not observe a consistent and significant root growth difference to wildtype in any of the tested phosphate concentration (S12 Fig).

Altogether, we provided the community with a quantification of natural variation of root growth and anion related Lotus phenotypes, showed that root system variation within a genotype by phosphate interaction is not specific to Arabidopsis but also occurs in a plant able to form AM symbiosis -even in the absence of the symbiont-, we generated a catalogue dataset of genes associated with root and anionic responses to phosphate, we investigated phenes and cross-links shaping Lotus natural variations of responses to phosphate and we genetically validated new candidate genes involved in phosphate accumulation. Lastly, a clear confounding element has been unveiled that could prevent future inappropriate conclusions.

## Materials and methods

### Plant material and growth conditions

In total, 130 *Lotus japonicus* accessions were used [23]. The names and accession numbers are listed in S6 Table. Seeds were scarified with sandpaper and then sterilized 14 minutes in 0.05% sodium hypochlorite. Subsequently, seeds were rinsed and washed 5 times in sterile distilled water. For the germination, seeds were positioned in imbibed filter paper, in sterile Petri dishes, and wrapped in aluminium foil. After 3 days at 21°C, young seedling were transferred to square plates (12 x 12 cm) containing growth medium (as described in [25]). Both media used in this study were based on Long-Ashton solution (with two levels of phosphate concentration -20 or 750 μM, LP or HP, respectively- as in S1 Table) with 0.8% MES buffer (Duchefa Biochemie, Haarlem, The Netherlands), 0.8% agarose (to minimize phosphate contamination), and adjusted to pH 5.7 with 1M KOH. After adding the medium, plates were dried, closed, overnight in a sterile laminar flow hood. Two accessions, with four replicates per each accession, were placed on each plate. Each plate was replicated, with mirrored position of each accession to minimize any positional growth effects. Plates were placed vertically, and plants grown under long-day conditions (21°C, 16 h light/8 h dark cycle) with white light bulbs emitting 50 μmol/m^2^/s and roots were exposed to light. Every day at the same time, the racks were transported to the image acquisition room where images of each plate were acquired with eight Epson V600 CCD flatbed color image scanners (Seiko Epson) and then immediately returned to the growth chamber. Each day, the position of plates was shuffled to avoid positional effects. LTR retrotransposon insertion LORE1 Lotus mutants were ordered from Lotus Base [38] and homozygous plants selected with specific primers (S9 Table). Since each mutant plant hosts more than one insertion, multiple mutants for each gene were selected for analysis.

For soil experiments, we used Einheitserde special substrate containing 200 mg/L of phosphate (P_2_O_5_), pH = 5.8, 60% of organic matter. The substrate is composed of acid peat, clay, perlite, calcareous corrective and mineral fertilizer (NPK). We watered the pots with tap water twice per week.

### Analysis of root growth and anion content

Each day at the same time, for 9 days, plates were scanned with CCD flatbed scanners (EPSON Perfection V600 Photo, Seiko Epson, Nagano, Japan), and the images were used to quantify root parameters using Brat 2.0 (as described in [25]). After 10 days, roots and shoots from 4 plants of each accession were weighed and frozen. For anion measurements in the initial screening, frozen plant material from 4 biological replicates was then homogenized in 1 mL of deionized water, and the anions -nitrate, phosphate and sulfate- were separated by the Dionex ICS-1100 chromatography system on a Dionex IonPac AS22 RFIC 4x250 mm analytic column (Thermo Scientific, Darmstadt, Germany) with 4.5 mM NaCO_3_/1.4 mM NaHCO_3_ as running buffer. Extraction with 1 mL 10 mM HCL did not affect the measured phosphate values. Total P was determined by inductively coupled plasma mass spectrometry (ICP-MS) from ca. 10 mg lyophilized shoot tissue by the Biocenter MS Platform Cologne, University of Cologne, using an Agilent 7700 ICP-MS (Agilent Technologies, Santa Clara, CA, USA) [14].

### Genome‐wide association studies and overlap analysis

GWA mapping was conducted on the mean and median trait values using a mixed model algorithm [31], which has been shown to correct for population structure confounding [32], and using the homozygous SNP data from the Lotus accessions [23]. SNPs with minor allele counts less than 10 were not taken into account. The significance of SNP associations was determined around the 5% FDR threshold computed by the Benjamini–Hochberg–Yekutieli method to correct for multiple testing [40] and genes within a 10-kb genomic region spanning each SNP were considered, taking into account that LD decays to 0.2 in Lotus [23]. We used the raw sequencing files [23] to investigate further SNPs associated with the phenotype, visualizing them in IGV [41] and following default setup for colour coding.

### Inorganic phosphate concentration measurements

Shoots and roots were collected, weighed and ground into a powder in liquid nitrogen. The powder was incubated at 98°C in NanoPure water, for 1 hr, centrifuged for 20 minutes at maximum speed. Then 25 uL of a dilution 1:10 were used to determine inorganic phosphate concentrations using the phosphate assay kit (Sigma, #MAK308), following instructions, as previously described [42]. Briefly, 50 uL of the reagents were added to our samples, left in darkness for 30 minutes and, subsequently, OD 620 was measured in a spectrophotometer. Each 96-well plate contained a calibration curve for assessing phosphate concentration.

### Figures and statistical analysis

Data analysis and plots were conducted in Rstudio (RStudio Team, 2016) using the following packages: tidyverse, emmeans, UpSetR, corrplot, RColorBrewer, rmarkdown, multcompView, ggpubr and gplots. Plots were further modified for colours and layout in Adobe Illustrator CS6. All the scripts used to generate raw figures can be found (S1 File) and raw measurements data (S10 Table). Number of replicates and statistical tests are indicated below every graph.

## Discussion

In this study, we generated a comprehensive atlas of root system architecture and nutrient accumulation responses to two levels of phosphate in 130 accessions of *Lotus japonicus* and studied trait relationships, their genetic basis and identified two genes controlling accumulation of phosphate. Overall, our results exposed general patterns of phenotypic and anion responses to phosphate, as well as significant natural variation in these responses across Lotus accessions, which importantly were not necessarily related to the Lotus subpopulation classes (Fig 1D and 1E). This indicates that population structure doesn’t confound to a large extent when studying responses to phosphate levels and don’t preclude screening for natural allelic variants that underlie these traits.

### Root responses to phosphate and the heritability of root and anion content traits

Low phosphate has been mostly associated with the inhibition of primary root growth and this process has been shown to be regulated by phosphate dependent iron accumulation [43]. However, it is not frequently considered that Arabidopsis accessions other than Col-0 are not showing any inhibition of primary root growth upon phosphate starvation [27], a finding that had indicated that this response is not canonical. In line with that, phosphate deficiency dependent inhibition of early root growth was not observed in our Lotus panel as LP does only have a minor effect on Lotus primary root growth (S3 Fig). This is consistent with previous reports for Lotus MG-20 ecotype [44]. Our data shows a broad variation of root growth responses among Lotus accessions, depending on phosphate availability. Altogether, this points towards possible different adaptive strategies that could have been selected in the Lotus natural populations in order to cope with phosphate starvation in their natural soil environments, in a similar manner to the natural variation of this response that has been described in Arabidopsis natural accessions.

Hierarchical clustering among accessions does depend on the phosphate level and not on the Lotus subpopulation (Fig 1D and 1E), indicating that the observed responses to phosphate are not just an expression of the kinship of these accessions.

Beyond highly heritable traits, such as flowering time [45] and seed dormancy [46], whose variation strongly depends on plant adaptation to environmental conditions, in the last years different studies successfully used GWA for identifying genes and alleles regulating both plant nutrient concentration and root growth traits. By measuring plant cadmium[47], sulfur [48,49], sodium [50] and phosphate [13] tissue concentration, causal genes were identified. A similar approach has been used to trace and map root growth responses to iron [51,52], salt [53], zinc [54], nitrogen [55] and phosphate [56] levels. In our attempts to integrate the two last approaches and recapitulate the natural variation of Lotus phosphate accumulation and root system architecture responses to phosphate levels, we observed a great variability among broad sense heritability between the two groups of traits (Fig 3A). Interestingly, in our set up, the majority of traits related to anions showed higher BSH compared to root growth related traits, with the exception of primary root growth length. Lower BSH reflects a higher trait variance within genotype compared to the trait variance found across genotypes. We therefore expect that traits showing higher BSH are either highly responsive to the external environmental conditions and factors not taken into account in our experimental set-up, such as plate micropatterning or seed size, or that our measurement error was too high for those traits. Another possible reason for the difference of BSH between these trait classes could be the different number of replicates: for the RSA analysis, we analysed 8 biological replicates, whereas the anion content was based on 4 biological replicates. Nevertheless, the higher BSH did not result in larger number of significantly associated SNPs for single anion traits (S13 Fig).

### Relation of phosphate content and root growth

During our investigation we found a correlation between phosphate content and growth-related traits exclusively in LP conditions (Fig 3C): the longer the root, the less concentrated the phosphate. The most likely explanation for this seems to be that we observe phosphate dilution effect in which the limited amount of P that is available in the plant is distributed over a larger amount of tissue in case of larger accessions. Dilution effects have been observed in a large number of plant species for many nutrient and environmentally related changes including interactions of biomass and nutrient abundance [29]. Our initial observation on a broad panel of accessions, became even more evident when focusing on a single genetic background (Gifu), where less genetic confounding effect are present (Fig 6). In this scenario, when plants are grown under low (20 μM) and mid (100 μM) phosphate, an even stronger and more significant negative correlation between plant biomass and plant phosphate concentration emerges. Again, this correlation is completely absent from plant grown under sufficient phosphate concentration (750 μM). While nutrient dilutions effects have been described before, our study system might be useful to enable genetic dissection of dilution effects. Overall, our findings are also an important reminder that plant biomass should always be taken into account when dealing with nutrient starvation condition, to avoid recursive confounding effects.

### Candidate genes for phosphate homeostasis

Phosphate is one of the main macronutrients and a limiting factor for plant growth, which is highly variable in natural and agricultural soils [57]. We therefore expect a strong selection on plant genomes due to soil phosphate concentration and/or soil microenvironment (both biotic and abiotic). Nevertheless, only few studies have identified causal genes involved in plant phosphate nutrition in the light of natural variation (for example [13,56,58]). By contrast, much more detailed knowledge has been acquired through forward genetics screening and transcriptomics approaches (mainly in Arabidopsis and rice) and subsequent validation of candidate genes.

Our GWAS analysis has detected hundreds of significant associations, among which are known regulators of plant root responses to low phosphate, such as *STOP1*. By combining candidate genes that were overlapping among traits, an approach that was similarly used in cereals [56], we selected and validated two of these, a Leucine-Rich-Repeat receptor kinase and a cytochrome B5 reductase. For each of these candidate genes, multiple LORE1 insertional mutants accumulate more soluble phosphate than the wt plants on high phosphate media (Fig 5). Despite allelic variation of the candidate genes being also associated with root traits, the mutants did not show aberrant root phenotypes. However, the absence of a root phenotype is not sufficient to conclude that there is no causality of the alleles for the observed root traits in the accessions. For instance, the activity of the minor allele in the reference genotype Gifu, which is wildtype for the LORE1 plants, might not be different enough from a loss of function allele, or redundancy or genetic buffering might compensate for these genes for early root growth, or the same loci might have a minor effect on RSA and a stronger effect on phosphate levels, and/or the same SNPs were in LD with other genes that control RSA. Because of this, it is not possible to conclude much regarding whether our overlapping strategy did or didn’t constitute an advantage over selecting candidate genes just from phosphate level GWAS hits. It might indeed be possible that there is no advantage of an overlap strategy, as phosphate dependent root growth responses are considered local responses and anion levels are considered to be strongly determined by systemic processes. However, our findings that among all the measured traits, root length is negatively correlated with phosphate concentration exclusively under phosphate limiting conditions in our screening population (130 Lotus accessions, ~1000 individual plants), and the strong negative correlation between plant biomass and phosphate concentration under phosphate limiting conditions indicate that phosphate starvation has an effect both on root length and on cellular phosphate concentration. It will therefore be very interesting to conduct more follow-up studies to explore these relations.

Regardless of this, the clear involvement of these genes in the control of root phosphate concentration exposes two new phosphate regulating genes, which are among the first phosphate regulators known in Lotus.

The Arabidopsis homologue of the cytochrome B5 reductase, CBR1 [59], was recently described as a crucial factor for iron uptake due to its role in activating plasma membrane H^+^-ATPase, responsible for acidification of the rhizosphere. CBR1 is involved in energy transfer at the ER level, it therefore could also control other important plant ion pumps that depends on electron potential. The inactivation of the Lotus homologue leads to the accumulation of phosphate in plant cells, even though the localization and the pool partitioning remains to be uncovered. Similarly, AVP1, a proton-pyrophosphatase gene, was found to confer increased shoot biomass in Arabidopsis under limiting P conditions [60]. What might be the causal polymorphisms in *CBR* that contribute to the natural variation of accumulation of phosphate in Lotus natural accessions? There are two prime candidate polymorphism that differentiate contrasting accessions accumulating more phosphate under LP: the lead SNP, which is located in the second intron and might have consequences on splicing or RNA expression, and a second SNP located in the 5^th^ exon coding sequence of CBR, which leads to an amino acid change and thereby might affect protein function (S14 Fig).

*LAMP*, the LRR-RK is involved in regulating internal plant phosphate levels and might therefore, similarly to other plant membrane receptors that regulate nitrogen metabolism in Arabidopsis and/or rhizobial abundance in Lotus [61,62], be involved in nutrient signalling. Lotus accessions with higher shoot:root phosphate ratio show the lead GWAS SNP in the promoter region together with many other SNPs that could affect transcriptional regulation. There are also three SNPs in the coding region of *LAMP* which cause amino-acids changes in the predicted transmembrane and kinase domains (S15 Fig). These allelic differences could be investigated further to dissect the causal mechanisms of the phenotypic variation caused by LAMP allelic variation.

In conclusion, we have made substantial headway in dissecting responses to phosphate starvation and their natural variation in *Lotus japonicus*. Our work exposed the natural variation and relations between root phenotypic responses and nutrient accumulation in Lotus, identified genes that play roles in the phosphate homeostasis in Lotus, and are a starting point for further functional studies to mechanistically understand the role of the genes we identified in phosphate uptake and homeostasis in Lotus.

## Supporting information

S1 Fig Sulfate and nitrate concentrations are not dependent on plant size. In the scatterplots, each dot represents a *Lotus japonicus* plant: its anion concentration on the y axis and its plant fresh weight on the x axis. The blue lines indicate linear regression and 90% confidence interval. On the left green panel *Lotus japonicus* natural accessions grown under low phosphate media (20 μM) and on the right blue panel plants grown under high phosphate media (750 μM). Only phosphate shows a moderate negative correlation, evident under low phosphate condition (ρ = -0.21), whereas the concentration of the other anions (nitrate and sulfate) is not correlated with variation in fresh plant weight. Graphs and correlation values were obtained with ggplot2 package using the Spearman method.
(TIF)

S2 Fig Correlation between phosphate and phosphorus concentration. Each dot represents the median concentration of phosphate and phosphorus in *Lotus japonicus* accessions. This value results from 3 or 4 biological replicates. On the top left corner, Pearson’s coefficient and p value are indicated. Colored lines and colored shades represent linear regression and 95% confidence intervals.
(TIF)

S3 Fig *Lotus japonicus* natural variation of root responses to phosphate media levels over time. Over a 9-day time course, Lotus natural accessions show a high diversity of root growth in LP and HP. a) Primary root growth over time. b) The width of the first 20% of the root (Root_20 width) and c) the width of the last 20% (Root_100 width) show an increase in plants grown in higher phosphate concentration compared to lower phosphate concentration.
(TIF)

S4 Fig *Lotus japonicu*s primary root length is negatively correlated with root phosphate concentration in plants grown under phosphate limitation. Considering the whole population level, primary root growth of Lotus accessions is negatively correlated with root phosphate concentration exclusively in plants grown in 20 μM of phosphate (r = -0.11, p = 0.03). By contrast, no correlation can be observed in plants grown in 750 μM of phosphate (r = -0.02, p = 0.59). Each point represents a single root measurement (root phosphate concentration and total primary root length are respectively on the x and y-axis).
(TIF)

S5 Fig Manhattan plots leading to the identification of the *CBR* locus. Manhattan plots depicting genome wide SNP associations for LP:HP ratio of root growth rate day 7 –day8, LP root width on day 1 and LP root phosphate concentration. The chromosomes are depicted in different colors. The horizontal blue dash-dot line corresponds to a nominal 0.05 significance threshold after Benjamini-Hochberg correction and the red dashed line corresponds to Bonferroni correction. Black boxes indicate the overlapping associated locus. x-axis: chromosomal position of SNP; x-axis: -log10(p-value).
(TIF)

S6 Fig Manhattan plots leading to the identification of the *LAMP* locus. Manhattan plots depicting genome wide SNP associations for LP root tortuosity and HP shoot:root phosphate concentration. The chromosomes are depicted in different colors. The horizontal blue dash-dot line corresponds to a nominal 0.05 significance threshold after Benjamini-Hochberg Correction and the red dashed line corresponds to Bonferroni correction. Black boxes indicate the overlapping associated locus. x-axis: chromosomal position of SNP; x-axis: -log10(p-value).
(TIF)

S7 Fig Genotyping of *cbr* and *lamp* mutant LORE1 insertion lines. a) Gene structure and insertion mutants used in this study for the cytochrome B5 reductase. Each number represents the Plant ID from Lotus Base. Three insertion mutants per gene were used. For the *cbr-2* line (plant ID 30106772), also wt segregant plants were selected. b) Gene structure and insertional mutants used in this study for the Leucine Rich Repeat Receptor Kinase LAMP. Each number represents the Plant ID from Lotus Base. Three insertion mutants per gene were used. For the *lamp-1* line (plant ID 3015040), also wt segregant plants were selected. After selecting homozygous plants, two progeny plants were reanalyzed to confirm the previous selection.
(TIF)

S8 Fig *cbr* and *lamp* mutants accumulate more phosphate than wt in high phosphate medium. a) Total phosphate concentration levels of root, shoot and total plants growing under low (20 μM), medium (100 μM) or high phosphate level (750 μM) for insertion mutants in *cbr* mutants. Most phosphate root concentrations are consistent with shoot phosphate concentrations. *cbr3* insertion lines shows higher phosphate concentration in the three tested medium conditions, whereas the other LORE1 mutants are condition dependent. b) Total phosphate concentration levels of root, shoot and total plants growing under low (20 μM), medium (100 μM) or high phosphate level (750 μM) for insertion mutants of *lamp*. All the three LORE1 insertional mutants display higher phosphate concentration at total plant level compared to wt. Each dot represents a single biological replicate and black vertical lines represent the median among the group.
(TIF)

S9 Fig Plant phosphate concentration is negatively correlated with plant biomass when phosphate is the limiting factor. Phosphate concentration levels of plants growing under low (20 μm, panel a) or medium (100 μM, panel b) phosphate is highly negatively correlated with plant biomass. By contrast under high phosphate level (750 μM, panel c), no significant correlation is observed. Each dot represents a single plant from different experiments. Phosphate concentration is calculated relative to wt roots phosphate concentration on 20 uM phosphate conditions. Colored lines and colored shades represent linear regression and 95% confidence intervals.
(TIF)

S10 Fig *cbr* and *lamp* mutant plants accumulate more phosphate than segregant wt plants in high phosphate medium independently from plant biomass. a) Plant phosphate concentration levels of wt and LORE1 *cytochrome B5 reductase* insertional mutant plants growing under low (20 μM) or medium (100 μM) or high phosphate level (750 μM). Whereas at low and medium concentration no significant difference is observed between wt and mutant homozygous segregant plants, at high phosphate concentration *cbr-2* is accumulating more phosphate than cbr2-wt plants. b) Plant phosphate concentration of *lamp* mutant plants and wt in the three phosphate media conditions. Each boxplot represents six biological replicates. *lamp-1* plants are accumulating more phosphate than wt plants both and middle and high levels of phosphate. Levels of phosphate are expressed relative to wt root plants at 20 μM. P-value from ANOVA test are indicated. c-d) Plant biomass (mg) of the four genotypes analyzed.
(TIF)

S11 Fig *cbr* and *lamp* mutants do not show consistent plant biomass differences compared to wt. a-b) Plant fresh weight (mg) and Tukey’s test of wt and LORE1 *cbr* insertion mutant plants growing under low (20 μM) or mid (100 μM) or high phosphate level (750 μM). Only *cbr-1* has a significant lower biomass compared to wt. c-d) Plant fresh weight (mg) and Tukey’s test of *lamp* mutant plants and wt in the three phosphate media conditions. At high phosphate concentration (750 μM), *lamp-2* shows significant lower biomass compared to wt. Each dot represents a single plant and black vertical lines represent the median among the group. Different letters in the stats panel represent different groups, following ANOVA test on estimated marginal means and Tukey’s adjusted p-value < 0.05.
(TIF)

S12 Fig *cbr* and *lamp* mutants are not affected in root growth over different phosphate concentration. a) Linear model of *lamp* primary root growth over time of plants growing under low (20 μM) or medium (100 μM) or high phosphate level (750 μM) based on data gathered over 5 time points on more than 5 replicates each. Shaded areas represent 95% confidence interval. b) Linear model of *cbr* primary root growth over time of plants growing under low (20 μM) or medium (100 μM) or high phosphate level (750 μM) based on data gathered over 5 time points on more than 5 replicate each. Shaded areas represent 95% confidence interval.
(TIF)

S13 Fig Number of significantly associated SNPs (p-value < FDR) per trait in anions vs. Brat analysis. Density plot representing the number of significant SNPs per each trait of RSA-related measurements compared to phosphate accumulation traits. No significant difference was observed between the two groups.
(TIF)

S14 Fig Natural *CBR* locus alleles and their association with root phosphate concentration across contrasting *L*. *japonicus* accessions. Dashed boxes highlight the shared genetic variation between the depicted accessions accumulating more root phosphate: the lead GWAS SNP (present in 32% of the Lotus accessions) in the second intron, and a SNP leading to an amino acid change in the 5^th^ exon. Each row visualizes sequence files for a single accession with coverage depth indicated on the y-axis and plotted in gray shades. Different colors represent SNPs compared to the reference accession MG20. Plotting according to IGV [41] default setup. Vertical lines with two colors indicate heterozygous sites. Horizontal bar plots on the right represent the average of root phosphate content under low phosphate conditions of the respective Lotus accessions. The *CBR* gene model is plotted at the bottom of the figure: exons in black, intron as lines and the direction of transcription indicated by arrows. Two different splicing forms are described in Lotus MG20 annotation.
(TIF)

S15 Fig Natural *LAMP* locus alleles and their association with shoot:root phosphate ratio across contrasting *L*. *japonicus* accessions. Dashed boxes highlight the shared genetic variation between the depicted accessions accumulating more shoot phosphate compared to the root phosphate content: the lead GWAS SNP (1kb upstream the coding sequence, present in 10% of the Lotus accessions) is within a region containing many SNPs in the predicted promoter region of *LAMP*. In the beginning of the coding sequence are two SNPs which cause amino acid changes. Towards the end of the coding region, in the putative kinase domain, there is another SNP causing an amino acid change, which is unique for the accessions that show higher values of shoot:root phosphate ratios. Each row visualizes sequence files for a single accession with coverage depth indicated on the y-axis and plotted in gray shades. Different colors represent SNPs compared to the reference accession MG20. Plotting according to IGV [41] default setup. Vertical lines with two colors indicate heterozygous sites. Horizontal bar plots on the right represent the average of shoot:root phosphate ratio root phosphate content under high phosphate conditions of the respective Lotus accessions. The *LAMP* gene model is plotted at the bottom of the figure: exons in black, intron as lines and the direction of transcription indicated by arrows.
(TIF)

S1 Table Modified Long-Ashton media solution. (XLSX)

S2 Table GWAS hits from LP:HP root growth traits ratios. (XLSX)

S3 Table GWAS hits from Lotus root growth traits on LP media. (XLSX)

S4 Table GWAS hits from Lotus root growth traits on HP media. (XLSX)

S5 Table GWAS hits from Lotus anion content. (XLSX)

S6 Table List of *Lotus japonicus* accessions. (XLSX)

S7 Table Summary statistics for *cbr* and *lamp* mutants. (XLSX)

S8 Table Top 500 SNPs from anion and root growth traits with p-value < 10^−5^. (XLSX)

S9 Table Primers and mutant plants used in this study. (XLSX)

S10 Table Raw data from root system analysis and anion measurements. (XLSX)

S11 Table GO enrichment for GWAS hits of root system traits from LP media. (XLSX)

S12 Table GO enrichment for GWAS hits of root system traits from HP media. (XLSX)

S13 Table List of significant SNPs crossing benjamini-Hochberg FDR threshold. (XLSX)

S14 Table List of root traits extracted and evaluated by BRAT. (XLSX)

S15 Table GWAS hits from LP median Root growth rate day7-8. (CSV)

S16 Table GWAS hits from LP mean Root width 80 day 1. (CSV)

S17 Table GWAS hits from LP mean Root tortuosity day 2. (CSV)

S18 Table GWAS hits from LP mean root phosphate concentration day 9. (CSV)

S19 Table GWAS hits from HP ratio shoot:root phosphate concentration day 9. (CSV)

S1 File List of R codes and plots used in this study (Rmd file). (PDF)

## References

[1] López-Bucio J, Hernández-Abreu E, Sánchez-Calderón L, Nieto-Jacobo MF, Simpson J, Herrera-Estrella L. Phosphate Availability Alters Architecture and Causes Changes in Hormone Sensitivity in the Arabidopsis Root System. Plant Physiol. 2002;129: 244–256. 10.1104/pp.010934 12011355 PMC155888

[2] Svistoonoff S, Creff A, Reymond M, Sigoillot-Claude C, Ricaud L, Blanchet A, et al Root tip contact with low-phosphate media reprograms plant root architecture. Nat Genet. 2007;39: 792–796. 10.1038/ng2041 17496893

[3] Wang X, Wang Z, Zheng Z, Dong J, Song L, Sui L, et al Genetic dissection of Fe-dependent signaling in root developmental responses to phosphate deficiency. Plant Physiol. 2019; pp.009072018. 10.1104/pp.18.00907 30420567 PMC6324241

[4] Müller J, Toev T, Heisters M, Teller J, Moore KL, Hause G, et al Iron-Dependent Callose Deposition Adjusts Root Meristem Maintenance to Phosphate Availability. Dev Cell. 2015;33: 216–230. 10.1016/j.devcel.2015.02.007 25898169

[5] Rubio V, Linhares F, Solano R, Martín AC, Iglesias J, Leyva A, et al A conserved MYB transcription factor involved in phosphate starvation signaling both in vascular plants and in unicellular algae. Genes Dev. 2001;15: 2122–2133. 10.1101/gad.204401 11511543 PMC312755

[6] Bustos R, Castrillo G, Linhares F, Puga MI, Rubio V, Pérez-Pérez J, et al A Central Regulatory System Largely Controls Transcriptional Activation and Repression Responses to Phosphate Starvation in Arabidopsis. PLOS Genet. 2010;6: e1001102 10.1371/journal.pgen.1001102 20838596 PMC2936532

[7] Puga MI, Mateos I, Charukesi R, Wang Z, Franco-Zorrilla JM, Lorenzo L de, et al SPX1 is a phosphate-dependent inhibitor of PHOSPHATE STARVATION RESPONSE 1 in Arabidopsis. Proc Natl Acad Sci. 2014;111: 14947–14952. 10.1073/pnas.1404654111 25271326 PMC4205628

[8] Wang Z, Ruan W, Shi J, Zhang L, Xiang D, Yang C, et al Rice SPX1 and SPX2 inhibit phosphate starvation responses through interacting with PHR2 in a phosphate-dependent manner. Proc Natl Acad Sci U S A. 2014;111: 14953–14958. 10.1073/pnas.1404680111 25271318 PMC4205599

[9] Lv Q, Zhong Y, Wang Y, Wang Z, Zhang L, Shi J, et al SPX4 Negatively Regulates Phosphate Signaling and Homeostasis through Its Interaction with PHR2 in Rice. Plant Cell. 2014;26: 1586–1597. 10.1105/tpc.114.123208 24692424 PMC4036573

[10] Essigmann B, Güler S, Narang RA, Linke D, Benning C. Phosphate availability affects the thylakoid lipid composition and the expression of SQD1, a gene required for sulfolipid biosynthesis in Arabidopsis thaliana. Proc Natl Acad Sci U S A. 1998;95: 1950–1955. 10.1073/pnas.95.4.1950 9465123 PMC19220

[11] Sakuraba Y, Kanno S, Mabuchi A, Monda K, Iba K, Yanagisawa S. A phytochrome-B-mediated regulatory mechanism of phosphorus acquisition. Nat Plants. 2018;4: 1089 10.1038/s41477-018-0294-7 30518831

[12] Briat J-F, Rouached H, Tissot N, Gaymard F, Dubos C. Integration of P, S, Fe, and Zn nutrition signals in Arabidopsis thaliana: potential involvement of PHOSPHATE STARVATION RESPONSE 1 (PHR1). Front Plant Sci. 2015;6 10.3389/fpls.2015.00290 25972885 PMC4411997

[13] Kisko M, Bouain N, Safi A, Medici A, Akkers RC, Secco D, et al LPCAT1 controls phosphate homeostasis in a zinc-dependent manner. Harrison MJ, editor. eLife. 2018;7: e32077 10.7554/eLife.32077 29453864 PMC5826268

[14] Almario J, Jeena G, Wunder J, Langen G, Zuccaro A, Coupland G, et al Root-associated fungal microbiota of nonmycorrhizal Arabis alpina and its contribution to plant phosphorus nutrition. Proc Natl Acad Sci. 2017;114: E9403–E9412. 10.1073/pnas.1710455114 28973917 PMC5676915

[15] Hiruma K, Gerlach N, Sacristán S, Nakano RT, Hacquard S, Kracher B, et al Root Endophyte Colletotrichum tofieldiae Confers Plant Fitness Benefits that Are Phosphate Status Dependent. Cell. 2016;165: 464–474. 10.1016/j.cell.2016.02.028 26997485 PMC4826447

[16] Castrillo G, Teixeira PJPL, Paredes SH, Law TF, de Lorenzo L, Feltcher ME, et al Root microbiota drive direct integration of phosphate stress and immunity. Nature. 2017;543: 513–518. 10.1038/nature21417 28297714 PMC5364063

[17] Paredes SH, Gao T, Law TF, Finkel OM, Mucyn T, Teixeira PJPL, et al Design of synthetic bacterial communities for predictable plant phenotypes. PLOS Biol. 2018;16: e2003962 10.1371/journal.pbio.2003962 29462153 PMC5819758

[18] Choi J, Summers W, Paszkowski U. Mechanisms Underlying Establishment of Arbuscular Mycorrhizal Symbioses. Annu Rev Phytopathol. 2018;56: 135–160. 10.1146/annurev-phyto-080516-035521 29856935

[19] Giovannetti M, Volpe V, Salvioli A, Bonfante P. Chapter 7—Fungal and Plant Tools for the Uptake of Nutrients in Arbuscular Mycorrhizas: A Molecular View In: Johnson NC, Gehring C, Jansa J, editors. Mycorrhizal Mediation of Soil. Elsevier; 2017 pp. 107–128. 10.1016/B978-0-12-804312-7.00007-3

[20] Lanfranco L, Fiorilli V, Gutjahr C. Partner communication and role of nutrients in the arbuscular mycorrhizal symbiosis. New Phytol. 2018;220: 1031–1046. 10.1111/nph.15230 29806959

[21] MacLean AM, Bravo A, Harrison MJ. Plant Signaling and Metabolic Pathways Enabling Arbuscular Mycorrhizal Symbiosis. Plant Cell. 2017;29: 2319–2335. 10.1105/tpc.17.00555 28855333 PMC5940448

[22] Carbonnel S, Gutjahr C. Control of arbuscular mycorrhiza development by nutrient signals. Front Plant Sci. 2014;5 10.3389/fpls.2014.00462 25309561 PMC4160938

[23] Shah N, Wakabayashi T, Kawamura Y, Skovbjerg CK, Wang M-Z, Mustamin Y, et al Extreme genetic signatures of local adaptation during plant colonization. bioRxiv. 2018; 485789 10.1101/485789 PMC6959357 31937774

[24] Kellermeier F, Armengaud P, Seditas TJ, Danku J, Salt DE, Amtmann A. Analysis of the Root System Architecture of Arabidopsis Provides a Quantitative Readout of Crosstalk between Nutritional Signals. Plant Cell. 2014;26: 1480–1496. 10.1105/tpc.113.122101 24692421 PMC4036566

[25] Giovannetti M, Małolepszy A, Göschl C, Busch W. Large-Scale Phenotyping of Root Traits in the Model Legume Lotus japonicus In: Busch W, editor. Plant Genomics: Methods and Protocols. New York, NY: Springer New York; 2017 pp. 155–167. 10.1007/978-1-4939-7003-2_11 28439863

[26] Slovak R, Göschl C, Su X, Shimotani K, Shiina T, Busch W. A Scalable Open-Source Pipeline for Large-Scale Root Phenotyping of Arabidopsis. Plant Cell. 2014;26: 2390–2403. 10.1105/tpc.114.124032 24920330 PMC4114940

[27] Chevalier F, Pata M, Nacry P, Doumas P, Rossignol M. Effects of phosphate availability on the root system architecture: large-scale analysis of the natural variation between Arabidopsis accessions. Plant Cell Environ. 2003;26: 1839–1850. 10.1046/j.1365-3040.2003.01100.x

[28] Ristova D, Giovannetti M, Metesch K, Busch W. Natural genetic variation shapes root system responses to phytohormones in Arabidopsis. Plant J. 2018;96: 468–481. 10.1111/tpj.14034 30030851 PMC6220887

[29] Jarrell WM, Beverly RB. The Dilution Effect in Plant Nutrition Studies In: Brady NC, editor. Advances in Agronomy. Academic Press; 1981 pp. 197–224. 10.1016/S0065-2113(08)60887-1

[30] Yu J, Pressoir G, Briggs WH, Vroh Bi I, Yamasaki M, Doebley JF, et al A unified mixed-model method for association mapping that accounts for multiple levels of relatedness. Nat Genet. 2006;38: 203–208. 10.1038/ng1702 16380716

[31] Kang HM, Zaitlen NA, Wade CM, Kirby A, Heckerman D, Daly MJ, et al Efficient Control of Population Structure in Model Organism Association Mapping. Genetics. 2008;178: 1709–1723. 10.1534/genetics.107.080101 18385116 PMC2278096

[32] Seren Ü, Vilhjálmsson BJ, Horton MW, Meng D, Forai P, Huang YS, et al GWAPP: A Web Application for Genome-Wide Association Mapping in Arabidopsis. Plant Cell. 2012;24: 4793–4805. 10.1105/tpc.112.108068 23277364 PMC3556958

[33] Di Laurenzio L, Wysocka-Diller J, Malamy JE, Pysh L, Helariutta Y, Freshour G, et al The SCARECROW gene regulates an asymmetric cell division that is essential for generating the radial organization of the Arabidopsis root. Cell. 1996;86: 423–433. 10.1016/s0092-8674(00)80115-4 8756724

[34] Cattaneo P, Hardtke CS. BIG BROTHER Uncouples Cell Proliferation from Elongation in the Arabidopsis Primary Root. Plant Cell Physiol. 2017;58: 1519–1527. 10.1093/pcp/pcx091 28922745 PMC5914324

[35] Mora-Macías J, Ojeda-Rivera JO, Gutiérrez-Alanís D, Yong-Villalobos L, Oropeza-Aburto A, Raya-González J, et al Malate-dependent Fe accumulation is a critical checkpoint in the root developmental response to low phosphate. Proc Natl Acad Sci. 2017;114: E3563–E3572. 10.1073/pnas.1701952114 28400510 PMC5410833

[36] Balzergue C, Dartevelle T, Godon C, Laugier E, Meisrimler C, Teulon J-M, et al Low phosphate activates STOP1-ALMT1 to rapidly inhibit root cell elongation. Nat Commun. 2017;8: 15300 10.1038/ncomms15300 28504266 PMC5440667

[37] Domergue F, Vishwanath SJ, Joubès J, Ono J, Lee JA, Bourdon M, et al Three Arabidopsis Fatty Acyl-Coenzyme A Reductases, FAR1, FAR4, and FAR5, Generate Primary Fatty Alcohols Associated with Suberin Deposition. Plant Physiol. 2010;153: 1539–1554. 10.1104/pp.110.158238 20571114 PMC2923872

[38] Mun T, Bachmann A, Gupta V, Stougaard J, Andersen SU. Lotus Base: An integrated information portal for the model legume Lotus japonicus. Sci Rep. 2016;6: 39447 10.1038/srep39447 28008948 PMC5180183

[39] Małolepszy A, Mun T, Sandal N, Gupta V, Dubin M, Urbański D, et al The LORE1 insertion mutant resource. Plant J. 2016;88: 306–317. 10.1111/tpj.13243 27322352

[40] Benjamini Y, Yekutieli D. The Control of the False Discovery Rate in Multiple Testing under Dependency. Ann Stat. 2001;29: 1165–1188.

[41] Robinson JT, Thorvaldsdóttir H, Winckler W, Guttman M, Lander ES, Getz G, et al Integrative genomics viewer. Nat Biotechnol. 2011;29: 24–26. 10.1038/nbt.1754 21221095 PMC3346182

[42] Ames BN. [10] Assay of inorganic phosphate, total phosphate and phosphatases Methods in Enzymology. Academic Press; 1966 pp. 115–118. 10.1016/0076-6879(66)08014-5

[43] Ward JT, Lahner B, Yakubova E, Salt DE, Raghothama KG. The Effect of Iron on the Primary Root Elongation of Arabidopsis during Phosphate Deficiency. Plant Physiol. 2008;147: 1181–1191. 10.1104/pp.108.118562 18467463 PMC2442553

[44] Volpe V, Giovannetti M, Sun X-G, Fiorilli V, Bonfante P. The phosphate transporters LjPT4 and MtPT4 mediate early root responses to phosphate status in non mycorrhizal roots. Plant Cell Environ. 2016;39: 660–671. 10.1111/pce.12659 26476189

[45] Atwell S, Huang YS, Vilhjálmsson BJ, Willems G, Horton M, Li Y, et al Genome-wide association study of 107 phenotypes in a common set of Arabidopsis thaliana inbred lines. Nature. 2010;465: 627–631. 10.1038/nature08800 20336072 PMC3023908

[46] Kerdaffrec E, Filiault DL, Korte A, Sasaki E, Nizhynska V, Seren Ü, et al Multiple alleles at a single locus control seed dormancy in Swedish Arabidopsis. Hardtke CS, editor. eLife. 2016;5: e22502 10.7554/eLife.22502 27966430 PMC5226650

[47] Chao D-Y, Silva A, Baxter I, Huang YS, Nordborg M, Danku J, et al Genome-Wide Association Studies Identify Heavy Metal ATPase3 as the Primary Determinant of Natural Variation in Leaf Cadmium in Arabidopsis thaliana. PLOS Genet. 2012;8: e1002923 10.1371/journal.pgen.1002923 22969436 PMC3435251

[48] Koprivova A, Giovannetti M, Baraniecka P, Lee B-R, Grondin C, Loudet O, et al Natural Variation in the ATPS1 Isoform of ATP Sulfurylase Contributes to the Control of Sulfate Levels in Arabidopsis. Plant Physiol. 2013;163: 1133–1141. 10.1104/pp.113.225748 24027241 PMC3813639

[49] Huang X-Y, Chao D-Y, Koprivova A, Danku J, Wirtz M, Müller S, et al Nuclear Localised MORE SULPHUR ACCUMULATION1 Epigenetically Regulates Sulphur Homeostasis in Arabidopsis thaliana. PLOS Genet. 2016;12: e1006298 10.1371/journal.pgen.1006298 27622452 PMC5021336

[50] Baxter I, Brazelton JN, Yu D, Huang YS, Lahner B, Yakubova E, et al A Coastal Cline in Sodium Accumulation in Arabidopsis thaliana Is Driven by Natural Variation of the Sodium Transporter AtHKT1;1. PLOS Genet. 2010;6: e1001193 10.1371/journal.pgen.1001193 21085628 PMC2978683

[51] Satbhai SB, Setzer C, Freynschlag F, Slovak R, Kerdaffrec E, Busch W. Natural allelic variation of FRO2 modulates Arabidopsis root growth under iron deficiency. Nat Commun. 2017;8: 15603 10.1038/ncomms15603 28537266 PMC5458102

[52] Li B, Sun L, Huang J, Göschl C, Shi W, Chory J, et al GSNOR provides plant tolerance to iron toxicity via preventing iron-dependent nitrosative and oxidative cytotoxicity. Nat Commun. 2019;10: 1–13. 10.1038/s41467-018-07882-8 31467270 PMC6715714

[53] Julkowska MM, Koevoets IT, Mol S, Hoefsloot H, Feron R, Tester MA, et al Genetic Components of Root Architecture Remodeling in Response to Salt Stress. Plant Cell. 2017;29: 3198–3213. 10.1105/tpc.16.00680 29114015 PMC5757256

[54] Bouain N, Satbhai SB, Korte A, Saenchai C, Desbrosses G, Berthomieu P, et al Natural allelic variation of the AZI1 gene controls root growth under zinc-limiting condition. PLOS Genet. 2018;14: e1007304 10.1371/journal.pgen.1007304 29608565 PMC5897037

[55] Gifford ML, Banta JA, Katari MS, Hulsmans J, Chen L, Ristova D, et al Plasticity Regulators Modulate Specific Root Traits in Discrete Nitrogen Environments. PLOS Genet. 2013;9: e1003760 10.1371/journal.pgen.1003760 24039603 PMC3764102

[56] Stetter MG, Schmid K, Ludewig U. Uncovering Genes and Ploidy Involved in the High Diversity in Root Hair Density, Length and Response to Local Scarce Phosphate in Arabidopsis thaliana. PLOS ONE. 2015;10: e0120604 10.1371/journal.pone.0120604 25781967 PMC4364354

[57] Orgiazzi A, Ballabio C, Panagos P, Jones A, Fernández‐Ugalde O. LUCAS Soil, the largest expandable soil dataset for Europe: a review. Eur J Soil Sci. 2018;69: 140–153. 10.1111/ejss.12499

[58] Yang M, Lu K, Zhao F-J, Xie W, Ramakrishna P, Wang G, et al Genome-Wide Association Studies Reveal the Genetic Basis of Ionomic Variation in Rice. Plant Cell. 2018;30: 2720–2740. 10.1105/tpc.18.00375 30373760 PMC6305983

[59] Oh YJ, Kim H, Seo SH, Hwang BG, Chang YS, Lee J, et al Cytochrome b5 Reductase 1 Triggers Serial Reactions that Lead to Iron Uptake in Plants. Mol Plant. 2016;9: 501–513. 10.1016/j.molp.2015.12.010 26712506

[60] Yang H, Zhang X, Gaxiola RA, Xu G, Peer WA, Murphy AS. Over-expression of the Arabidopsis proton-pyrophosphatase AVP1 enhances transplant survival, root mass, and fruit development under limiting phosphorus conditions. J Exp Bot. 2014;65: 3045–3053. 10.1093/jxb/eru149 24723407 PMC4071825

[61] Tabata R, Sumida K, Yoshii T, Ohyama K, Shinohara H, Matsubayashi Y. Perception of root-derived peptides by shoot LRR-RKs mediates systemic N-demand signaling. Science. 2014;346: 343–346. 10.1126/science.1257800 25324386

[62] Okamoto S, Shinohara H, Mori T, Matsubayashi Y, Kawaguchi M. Root-derived CLE glycopeptides control nodulation by direct binding to HAR1 receptor kinase. Nat Commun. 2013;4: 2191 10.1038/ncomms3191 23934307

