# Supplementary figures and images for: Identification of novel genes involved in phosphate accumulation in Lotus japonicus through Genome Wide Association mapping of root system architecture and anion content
Source: PLoS Genet. 2019 Dec 19;15(12):e1008126. doi: 10.1371/journal.pgen.1008126 (PMC6941899; doi:10.1371/journal.pgen.1008126)

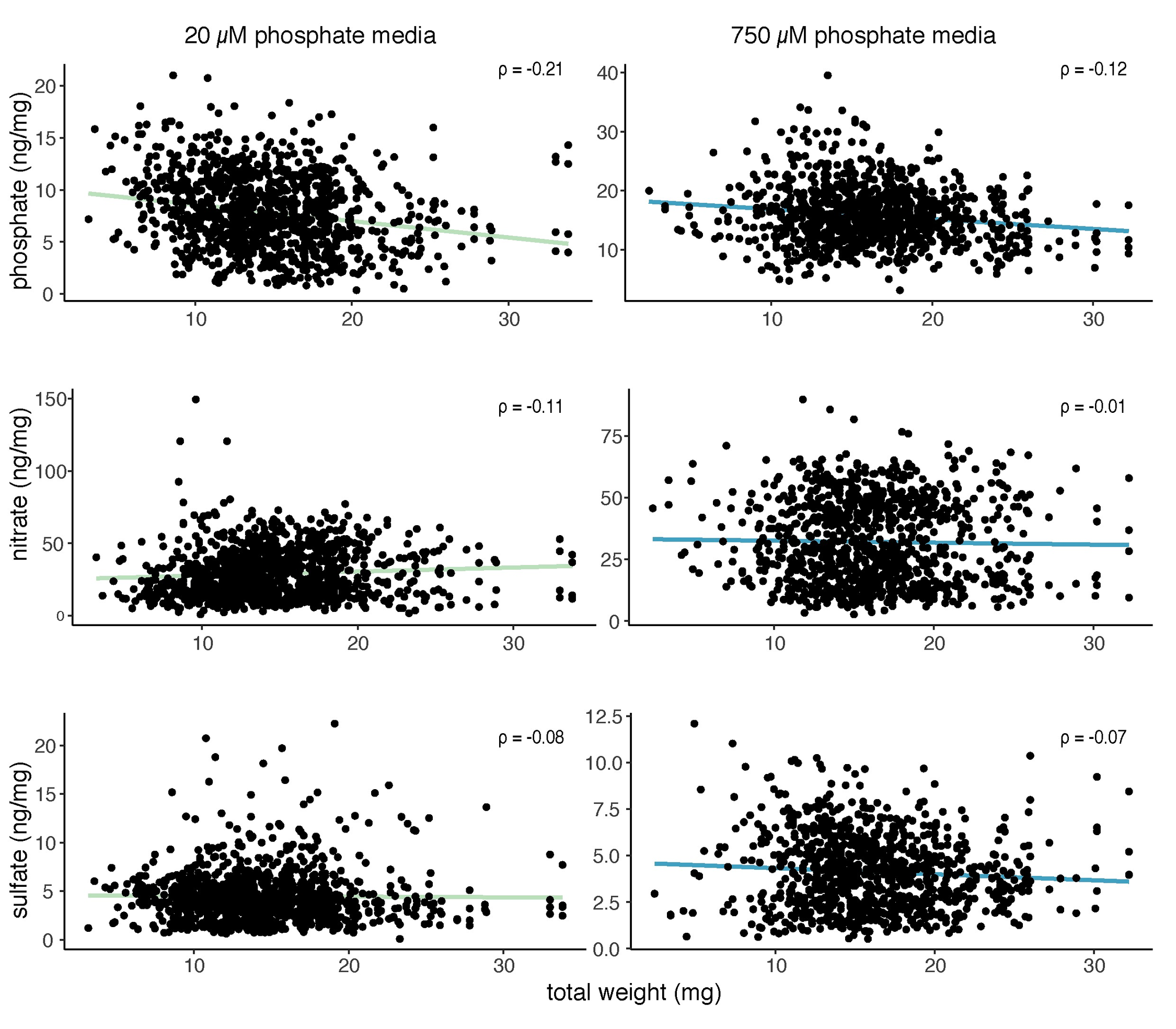

Supplement: S1 Fig — In the scatterplots, each dot represents a Lotus japonicus plant: its anion concentration on the y axis and its plant fresh weight on the x axis. The blue lines indicate linear regression and 90% confidence interval. On the left green panel Lotus japonicus natural accessions grown under low phosphate media (20 μM) and on the right blue panel plants grown under high phosphate media (750 μM). Only phosphate shows a moderate negative correlation, evident under low phosphate condition (ρ = -0.21), whereas the concentration of the other anions (nitrate and sulfate) is not correlated with variation in fresh plant weight. Graphs and correlation values were obtained with ggplot2 package using the Spearman method. (TIF) [file pgen.1008126.s001.tif]

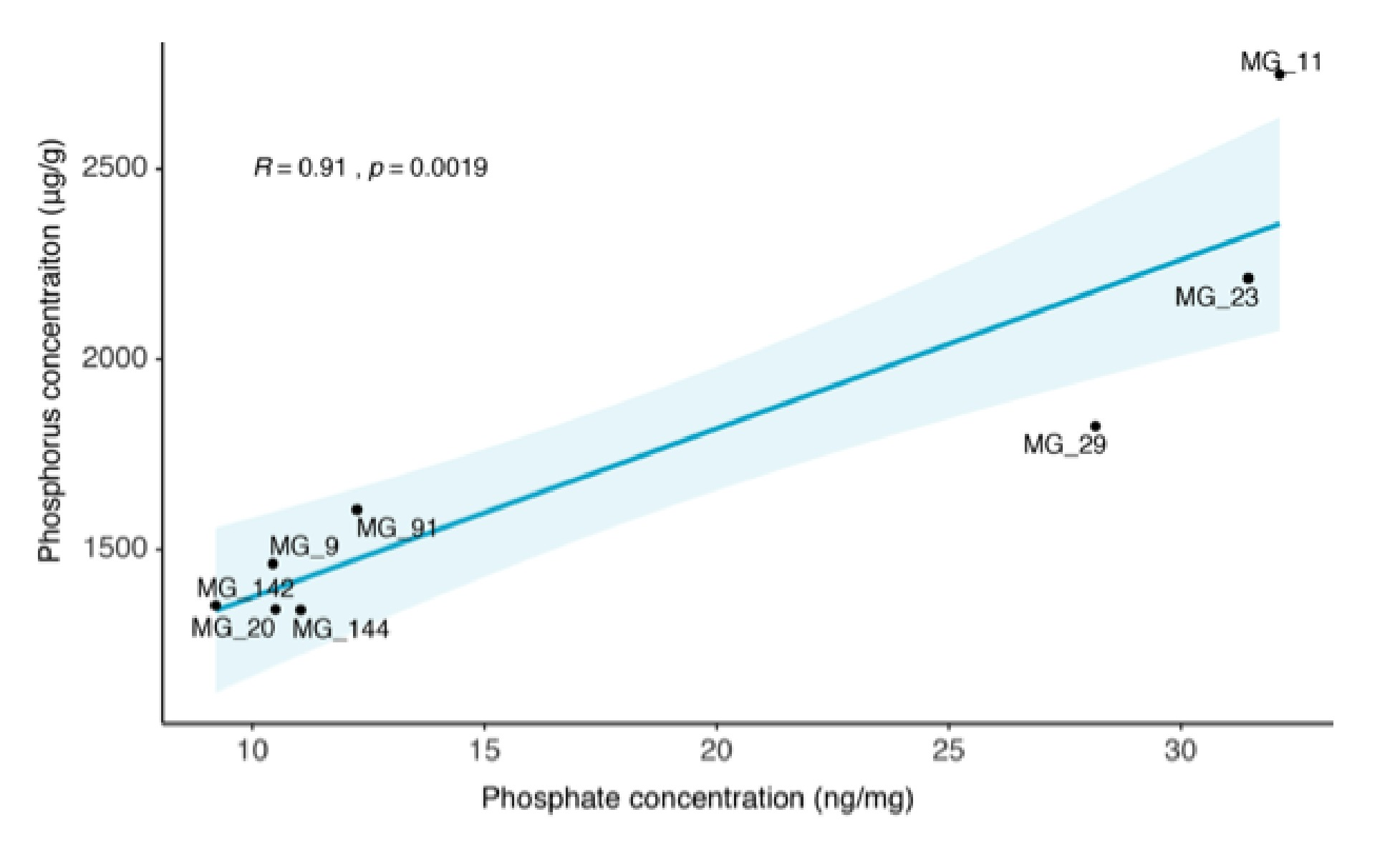

Supplement: S2 Fig — Each dot represents the median concentration of phosphate and phosphorus in Lotus japonicus accessions. This value results from 3 or 4 biological replicates. On the top left corner, Pearson’s coefficient and p value are indicated. Colored lines and colored shades represent linear regression and 95% confidence intervals. (TIF) [file pgen.1008126.s002.tif]

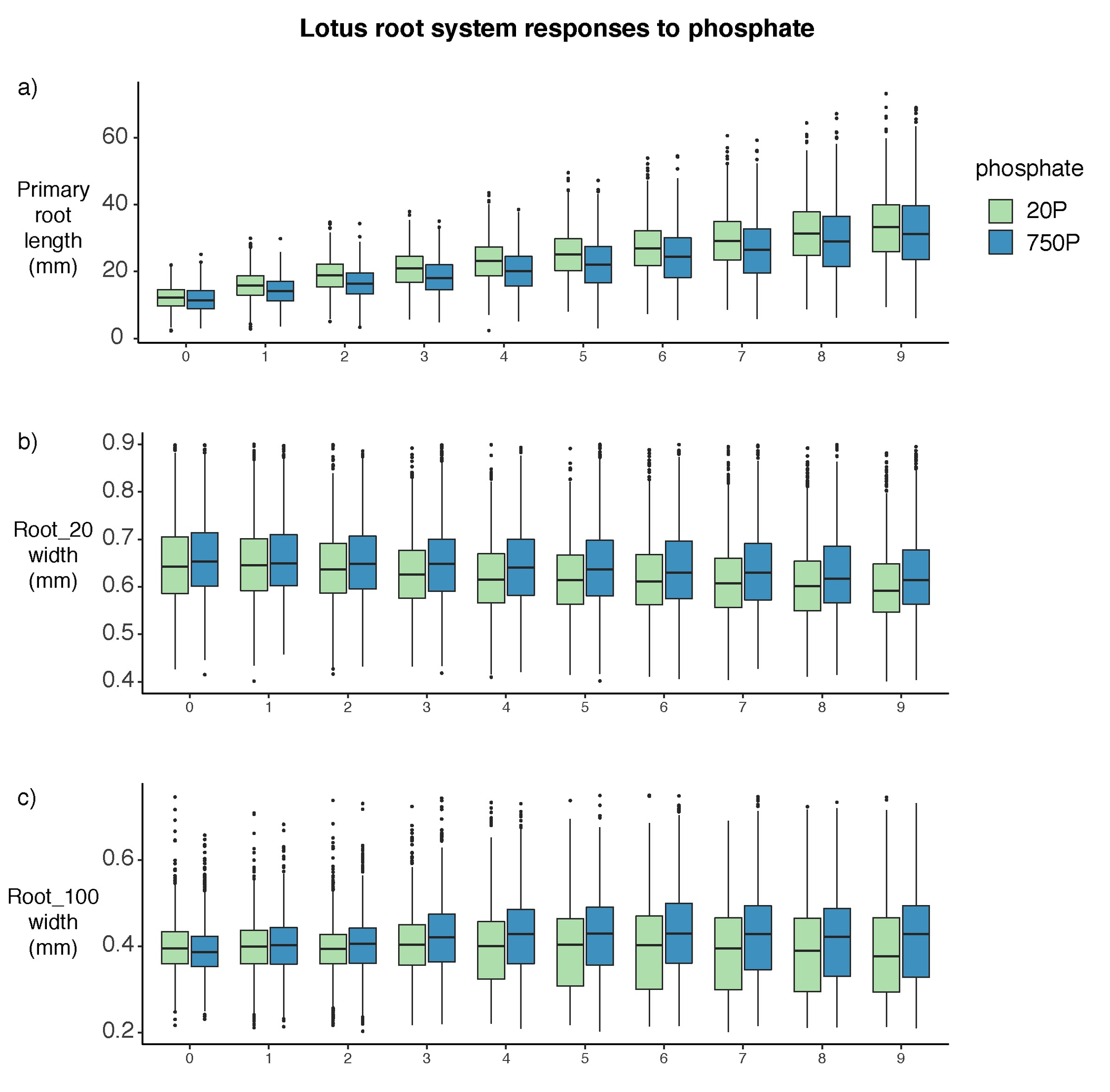

Supplement: S3 Fig — Over a 9-day time course, Lotus natural accessions show a high diversity of root growth in LP and HP. a) Primary root growth over time. b) The width of the first 20% of the root (Root_20 width) and c) the width of the last 20% (Root_100 width) show an increase in plants grown in higher phosphate concentration compared to lower phosphate concentration. (TIF) [file pgen.1008126.s003.tif]

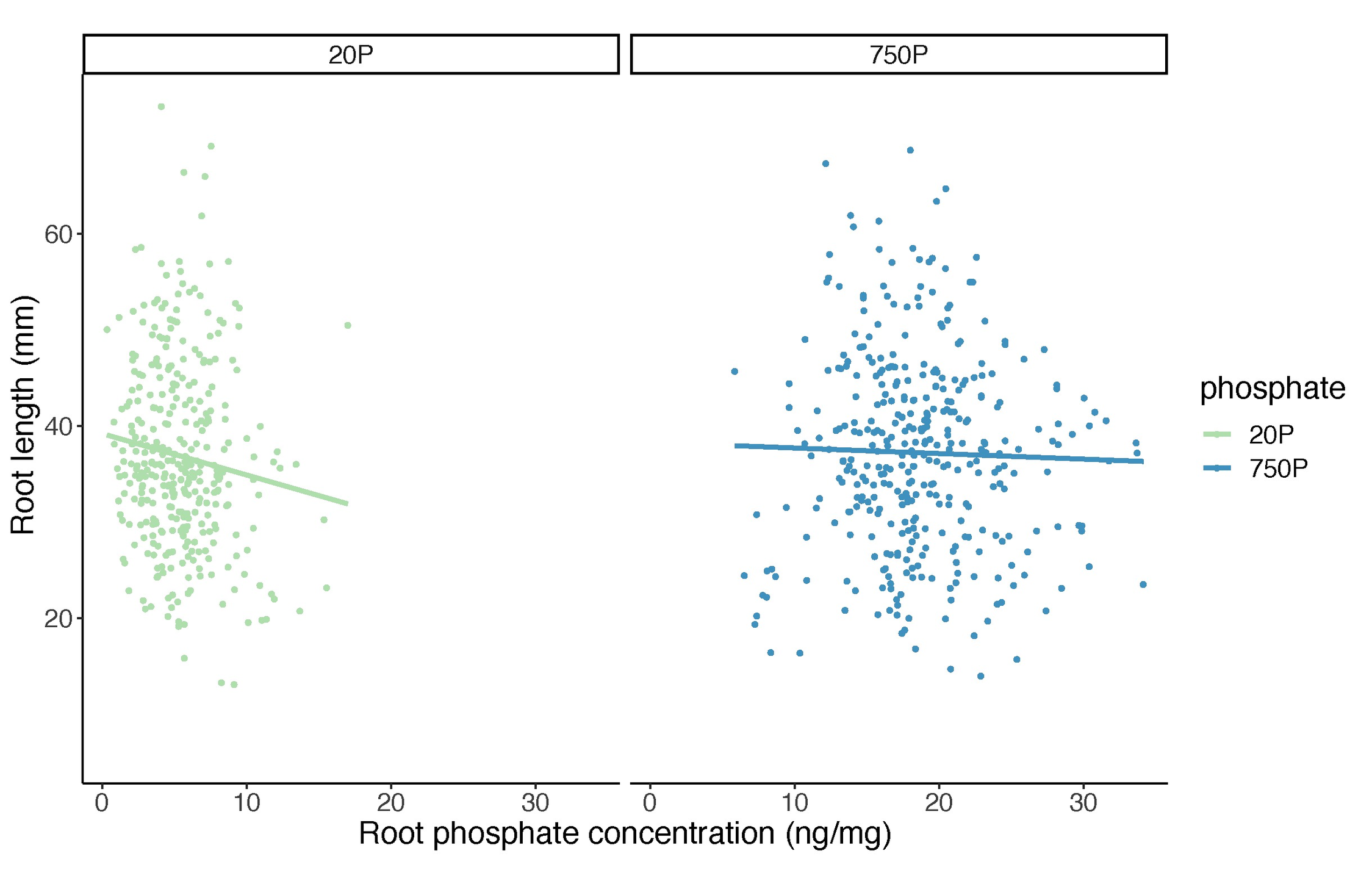

Supplement: S4 Fig — Considering the whole population level, primary root growth of Lotus accessions is negatively correlated with root phosphate concentration exclusively in plants grown in 20 μM of phosphate (r = -0.11, p = 0.03). By contrast, no correlation can be observed in plants grown in 750 μM of phosphate (r = -0.02, p = 0.59). Each point represents a single root measurement (root phosphate concentration and total primary root length are respectively on the x and y-axis). (TIF) [file pgen.1008126.s004.tif]

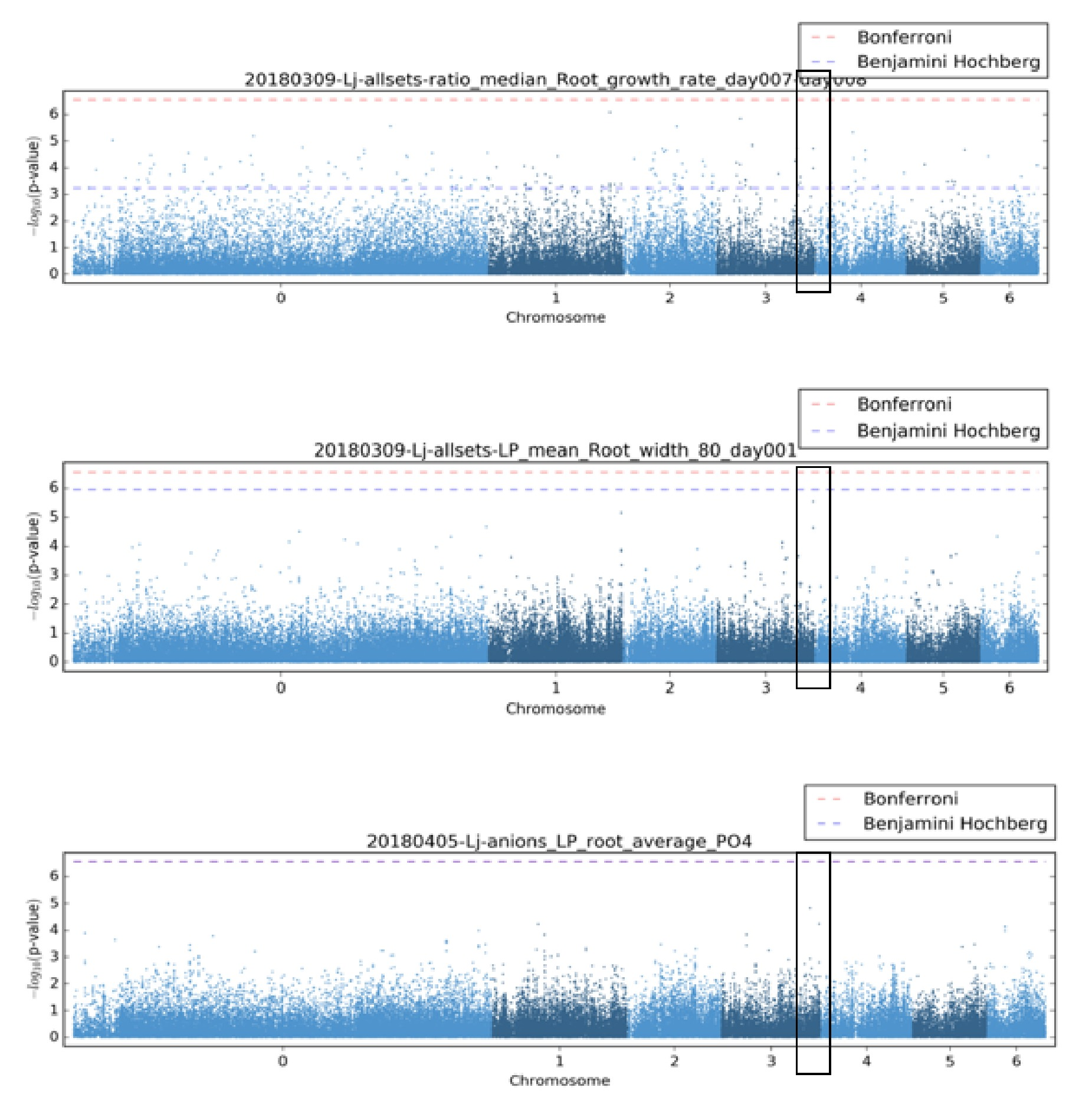

Supplement: S5 Fig — Manhattan plots depicting genome wide SNP associations for LP:HP ratio of root growth rate day 7 –day8, LP root width on day 1 and LP root phosphate concentration. The chromosomes are depicted in different colors. The horizontal blue dash-dot line corresponds to a nominal 0.05 significance threshold after Benjamini-Hochberg correction and the red dashed line corresponds to Bonferroni correction. Black boxes indicate the overlapping associated locus. x-axis: chromosomal position of SNP; x-axis: -log10(p-value). (TIF) [file pgen.1008126.s005.tif]

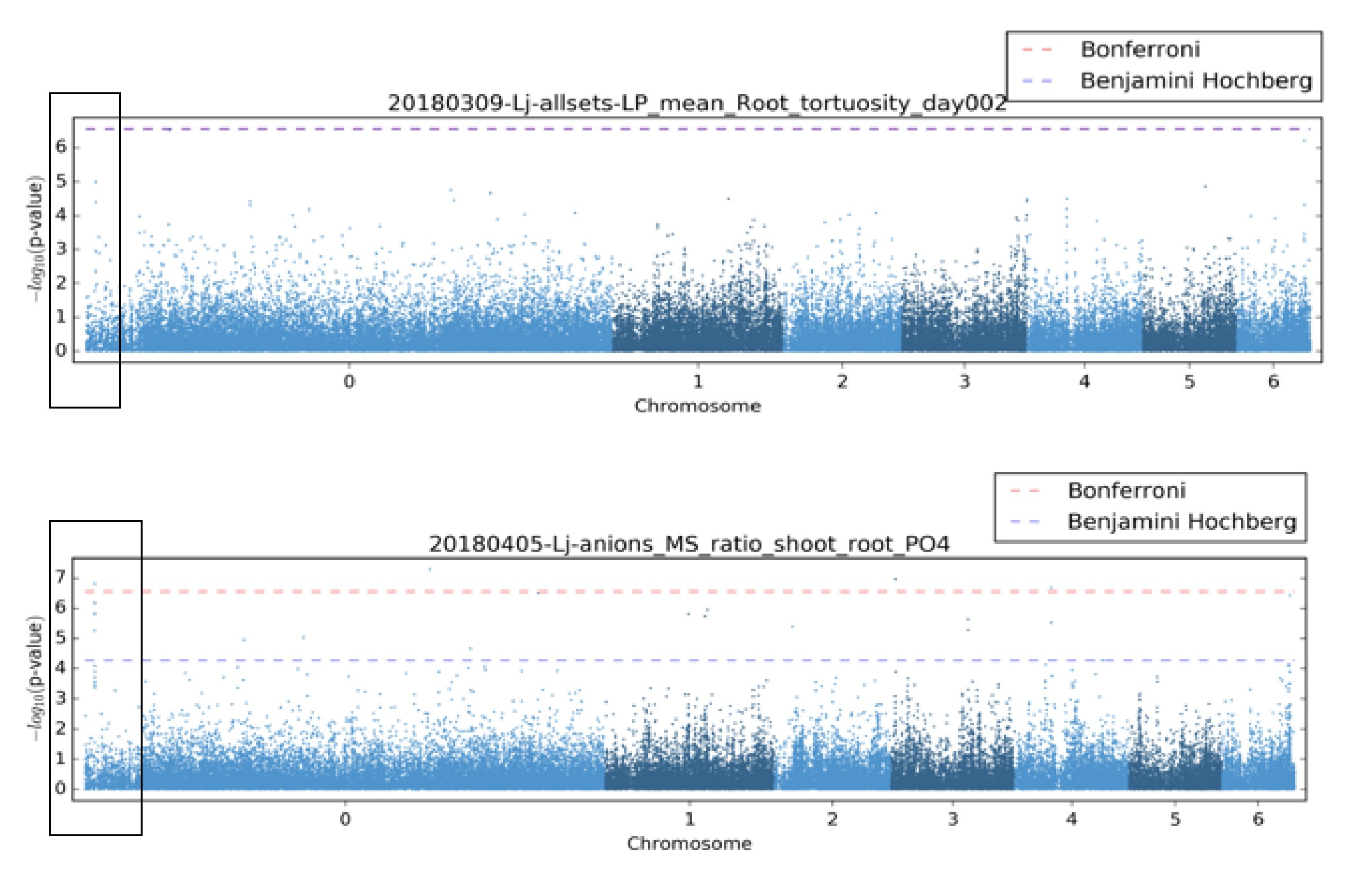

Supplement: S6 Fig — Manhattan plots depicting genome wide SNP associations for LP root tortuosity and HP shoot:root phosphate concentration. The chromosomes are depicted in different colors. The horizontal blue dash-dot line corresponds to a nominal 0.05 significance threshold after Benjamini-Hochberg Correction and the red dashed line corresponds to Bonferroni correction. Black boxes indicate the overlapping associated locus. x-axis: chromosomal position of SNP; x-axis: -log10(p-value). (TIF) [file pgen.1008126.s006.tif]

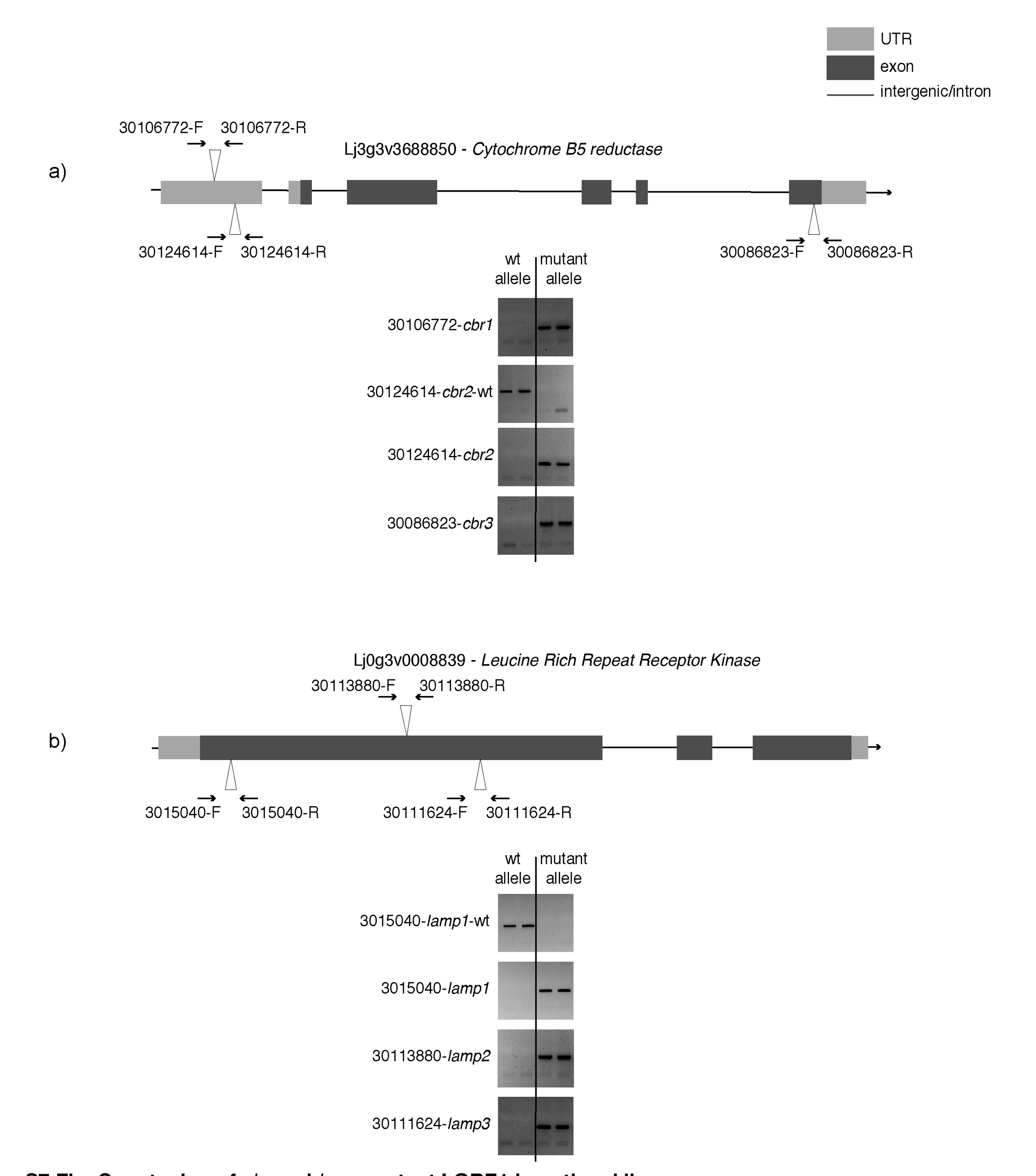

Supplement: S7 Fig — a) Gene structure and insertion mutants used in this study for the cytochrome B5 reductase. Each number represents the Plant ID from Lotus Base. Three insertion mutants per gene were used. For the cbr-2 line (plant ID 30106772), also wt segregant plants were selected. b) Gene structure and insertional mutants used in this study for the Leucine Rich Repeat Receptor Kinase LAMP. Each number represents the Plant ID from Lotus Base. Three insertion mutants per gene were used. For the lamp-1 line (plant ID 3015040), also wt segregant plants were selected. After selecting homozygous plants, two progeny plants were reanalyzed to confirm the previous selection. (TIF) [file pgen.1008126.s007.tif]

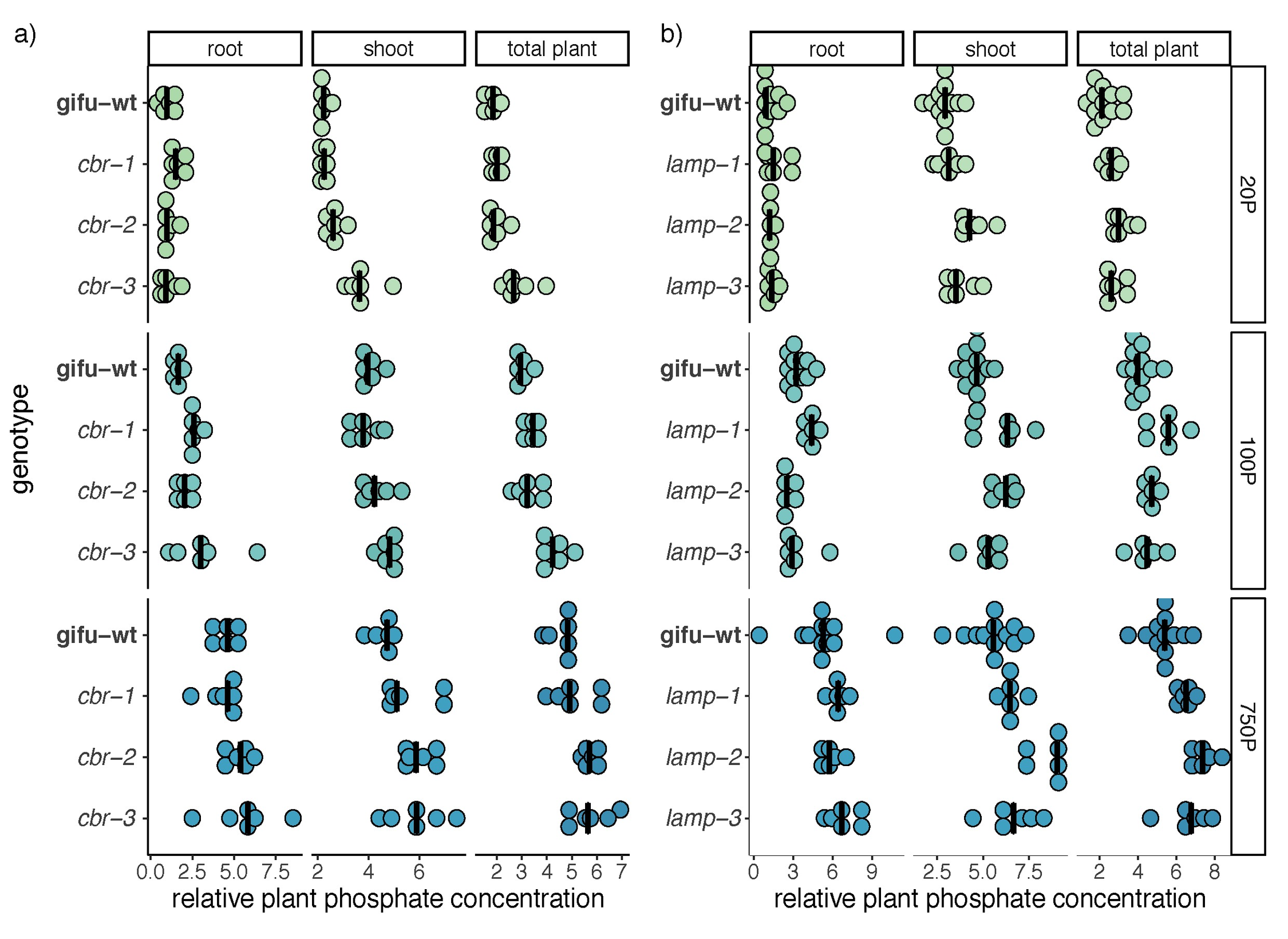

Supplement: S8 Fig — a) Total phosphate concentration levels of root, shoot and total plants growing under low (20 μM), medium (100 μM) or high phosphate level (750 μM) for insertion mutants in cbr mutants. Most phosphate root concentrations are consistent with shoot phosphate concentrations. cbr3 insertion lines shows higher phosphate concentration in the three tested medium conditions, whereas the other LORE1 mutants are condition dependent. b) Total phosphate concentration levels of root, shoot and total plants growing under low (20 μM), medium (100 μM) or high phosphate level (750 μM) for insertion mutants of lamp. All the three LORE1 insertional mutants display higher phosphate concentration at total plant level compared to wt. Each dot represents a single biological replicate and black vertical lines represent the median among the group. (TIF) [file pgen.1008126.s008.tif]

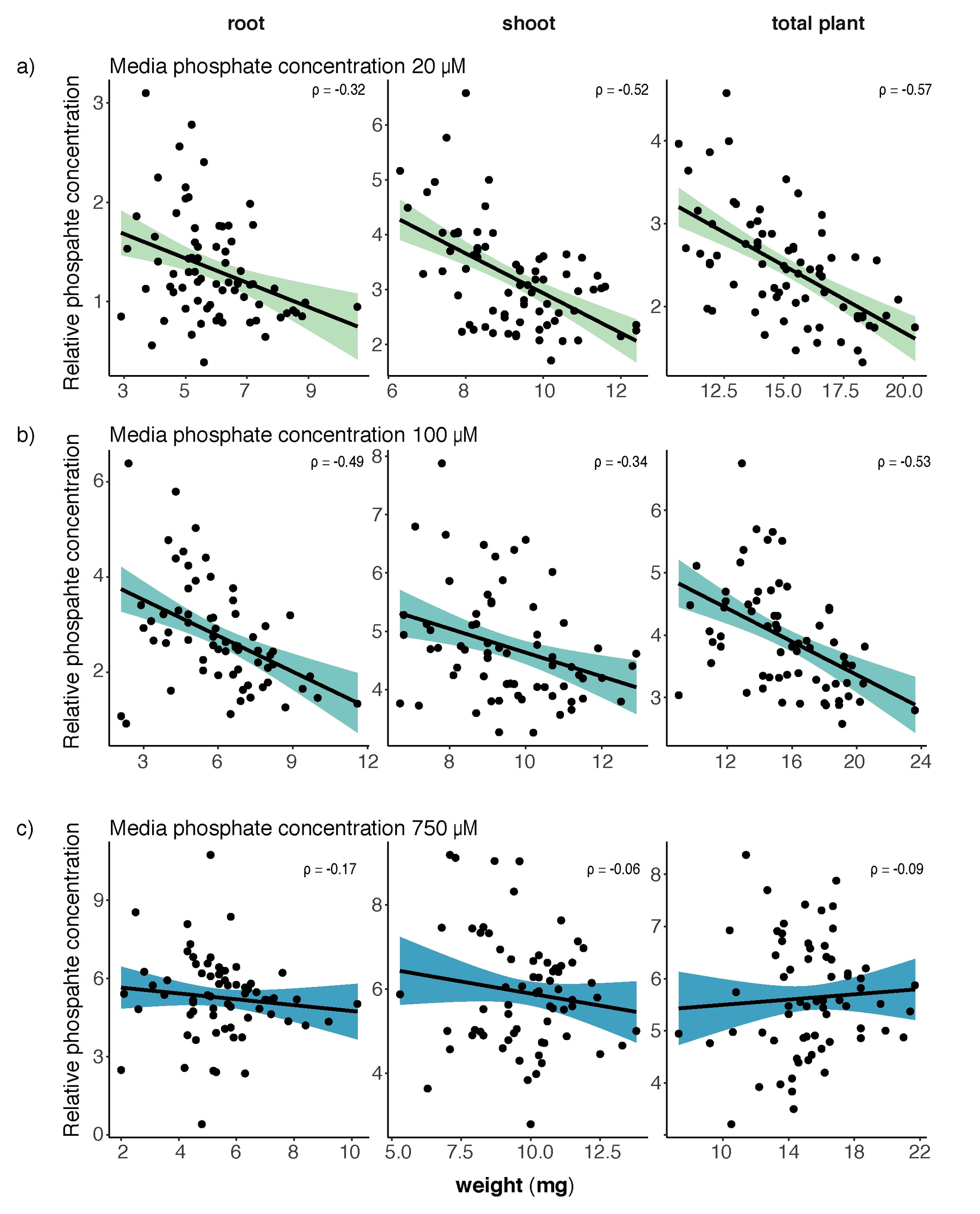

Supplement: S9 Fig — Phosphate concentration levels of plants growing under low (20 μm, panel a) or medium (100 μM, panel b) phosphate is highly negatively correlated with plant biomass. By contrast under high phosphate level (750 μM, panel c), no significant correlation is observed. Each dot represents a single plant from different experiments. Phosphate concentration is calculated relative to wt roots phosphate concentration on 20 uM phosphate conditions. Colored lines and colored shades represent linear regression and 95% confidence intervals. (TIF) [file pgen.1008126.s009.tif]

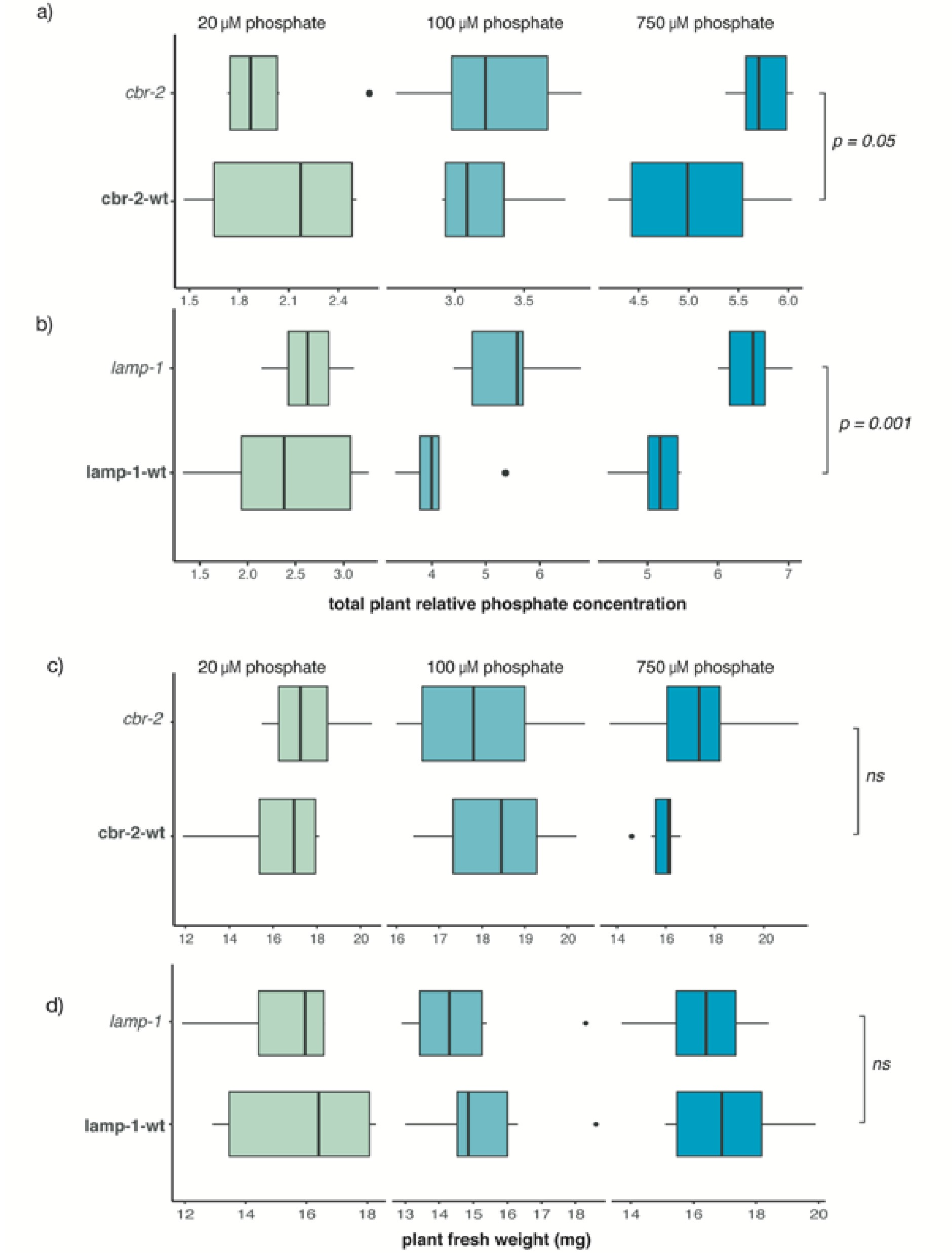

Supplement: S10 Fig — a) Plant phosphate concentration levels of wt and LORE1 cytochrome B5 reductase insertional mutant plants growing under low (20 μM) or medium (100 μM) or high phosphate level (750 μM). Whereas at low and medium concentration no significant difference is observed between wt and mutant homozygous segregant plants, at high phosphate concentration cbr-2 is accumulating more phosphate than cbr2-wt plants. b) Plant phosphate concentration of lamp mutant plants and wt in the three phosphate media conditions. Each boxplot represents six biological replicates. lamp-1 plants are accumulating more phosphate than wt plants both and middle and high levels of phosphate. Levels of phosphate are expressed relative to wt root plants at 20 μM. P-value from ANOVA test are indicated. c-d) Plant biomass (mg) of the four genotypes analyzed. (TIF) [file pgen.1008126.s010.tif]

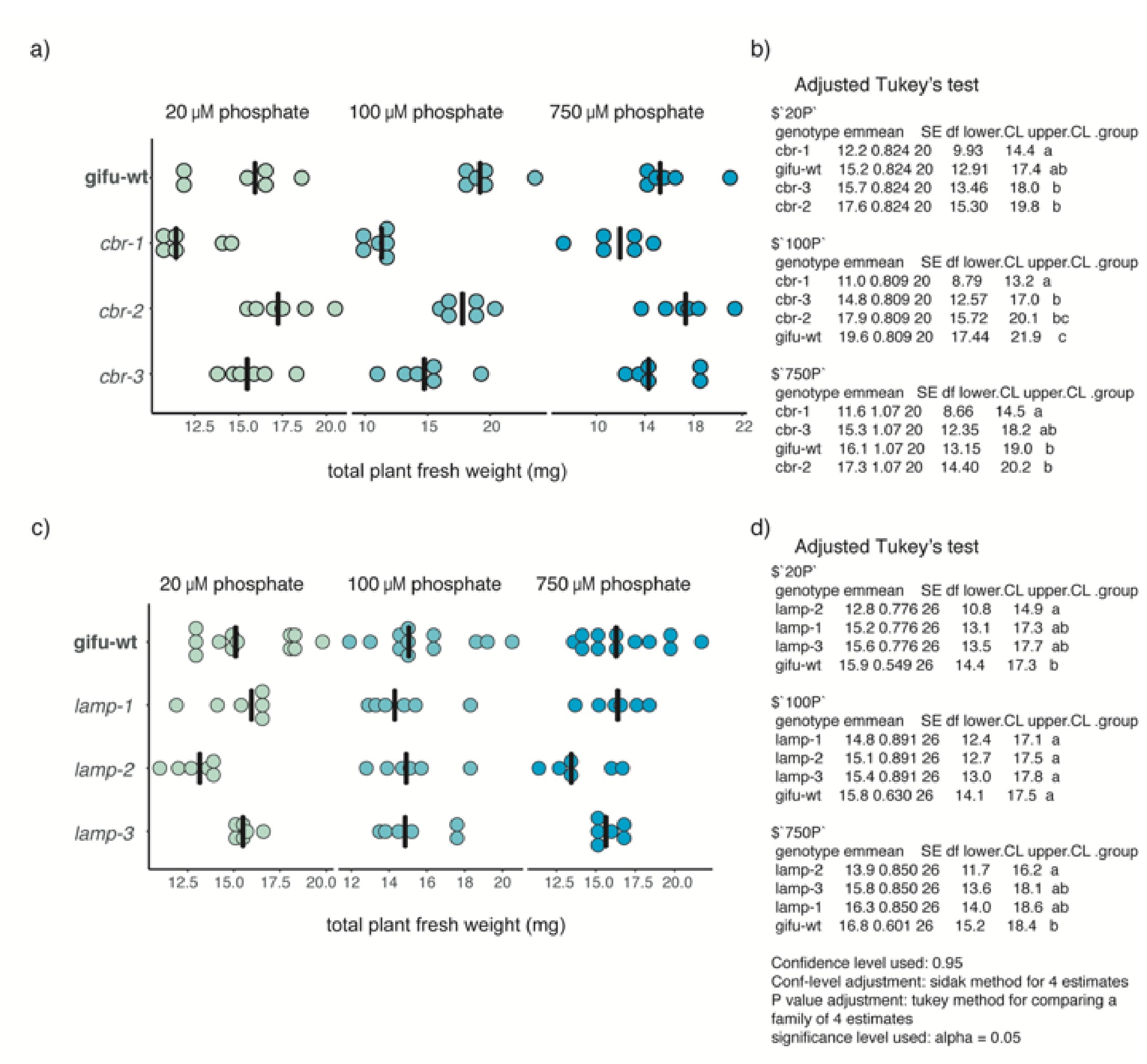

Supplement: S11 Fig — a-b) Plant fresh weight (mg) and Tukey’s test of wt and LORE1 cbr insertion mutant plants growing under low (20 μM) or mid (100 μM) or high phosphate level (750 μM). Only cbr-1 has a significant lower biomass compared to wt. c-d) Plant fresh weight (mg) and Tukey’s test of lamp mutant plants and wt in the three phosphate media conditions. At high phosphate concentration (750 μM), lamp-2 shows significant lower biomass compared to wt. Each dot represents a single plant and black vertical lines represent the median among the group. Different letters in the stats panel represent different groups, following ANOVA test on estimated marginal means and Tukey’s adjusted p-value < 0.05. (TIF) [file pgen.1008126.s011.tif]

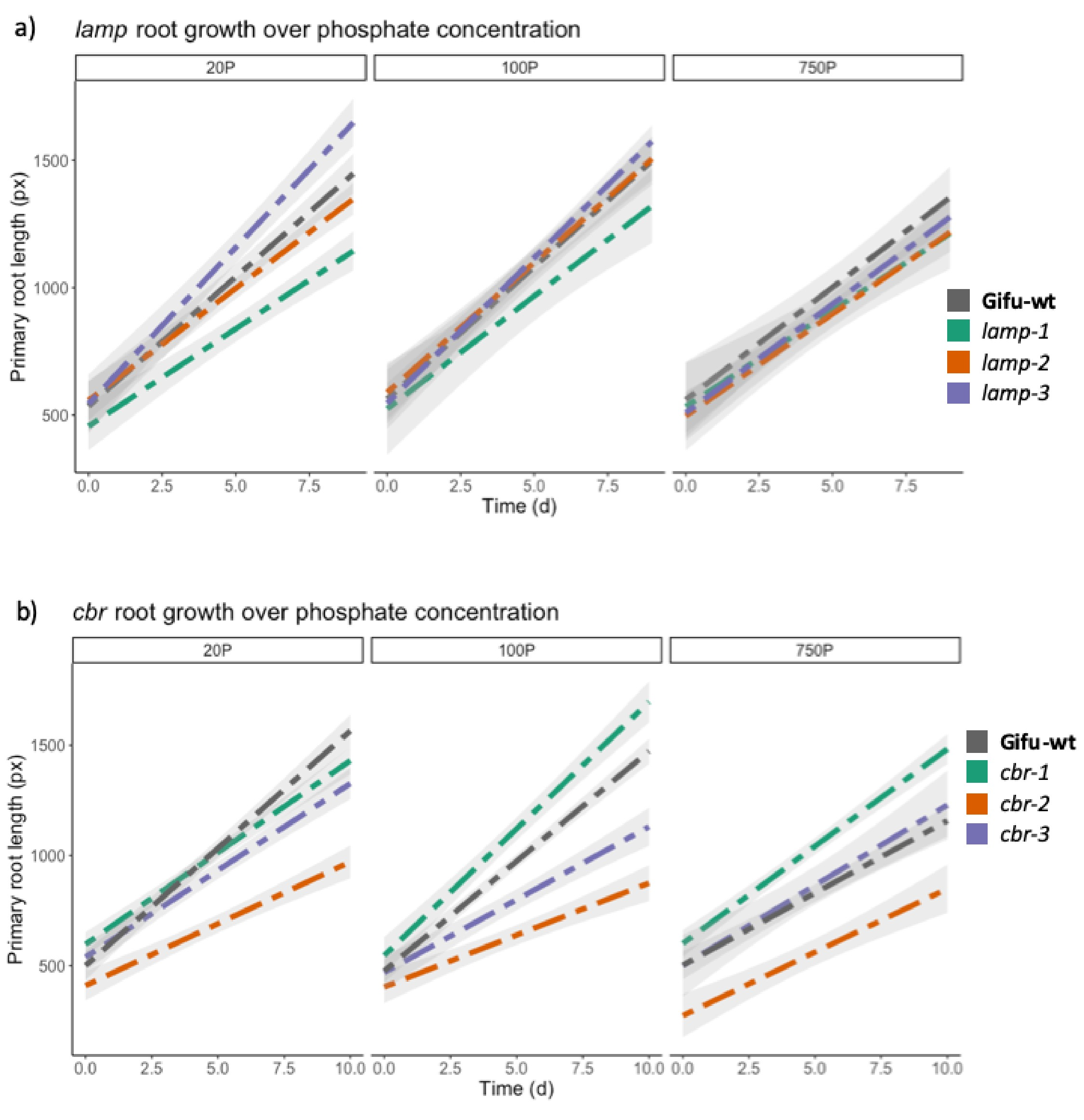

Supplement: S12 Fig — a) Linear model of lamp primary root growth over time of plants growing under low (20 μM) or medium (100 μM) or high phosphate level (750 μM) based on data gathered over 5 time points on more than 5 replicates each. Shaded areas represent 95% confidence interval. b) Linear model of cbr primary root growth over time of plants growing under low (20 μM) or medium (100 μM) or high phosphate level (750 μM) based on data gathered over 5 time points on more than 5 replicate each. Shaded areas represent 95% confidence interval. (TIF) [file pgen.1008126.s012.tif]

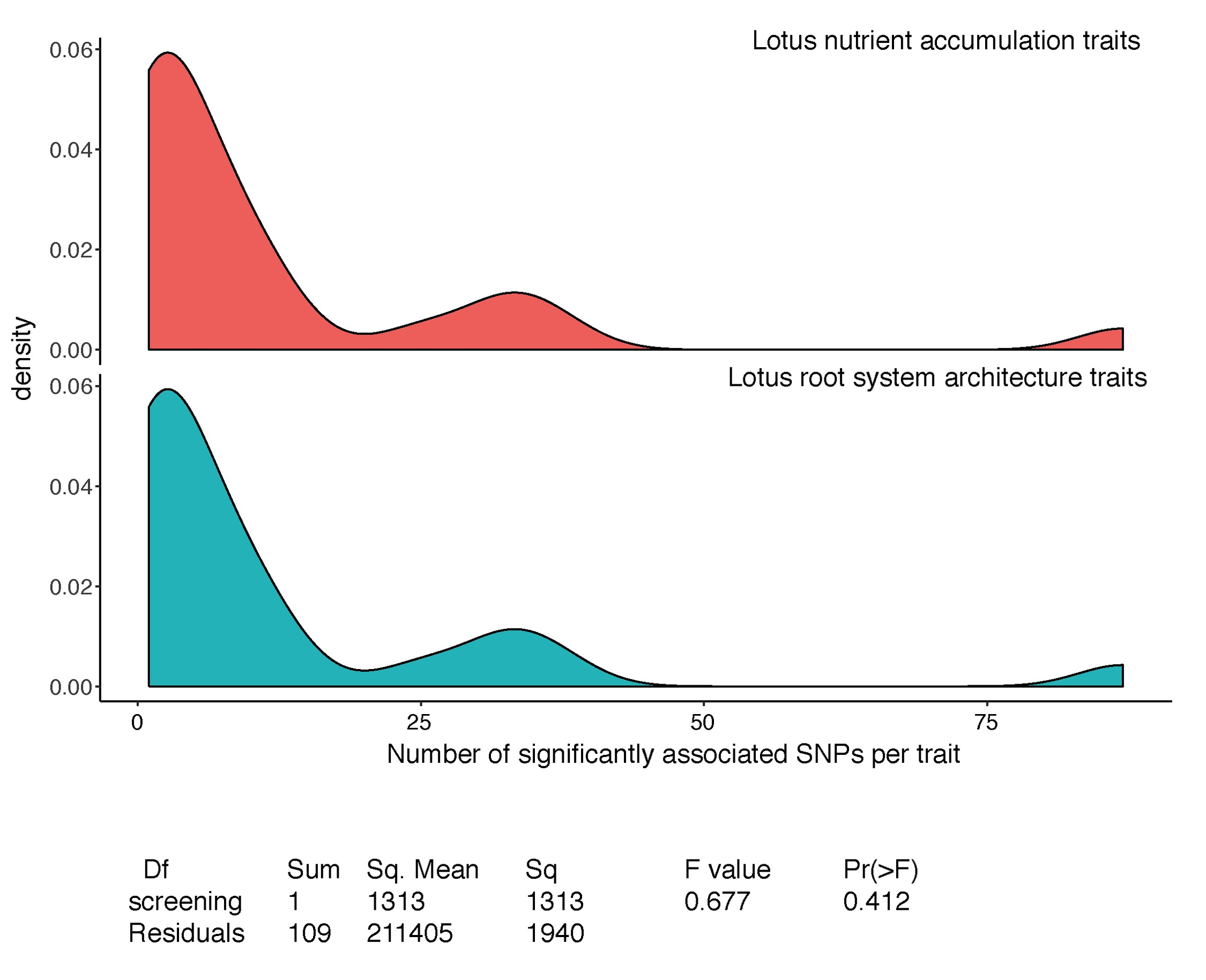

Supplement: S13 Fig — Density plot representing the number of significant SNPs per each trait of RSA-related measurements compared to phosphate accumulation traits. No significant difference was observed between the two groups. (TIF) [file pgen.1008126.s013.tif]

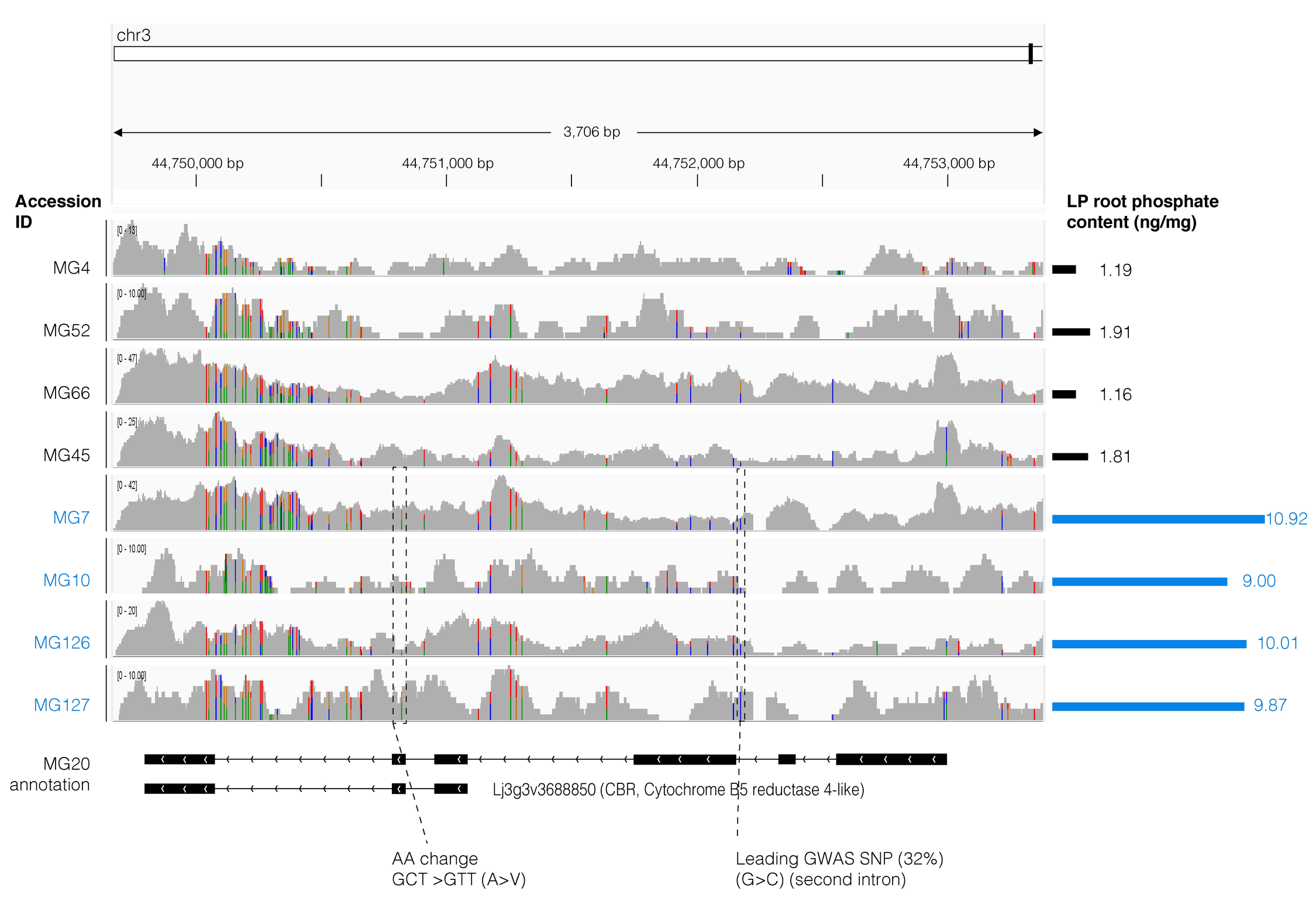

Supplement: S14 Fig — Dashed boxes highlight the shared genetic variation between the depicted accessions accumulating more root phosphate: the lead GWAS SNP (present in 32% of the Lotus accessions) in the second intron, and a SNP leading to an amino acid change in the 5th exon. Each row visualizes sequence files for a single accession with coverage depth indicated on the y-axis and plotted in gray shades. Different colors represent SNPs compared to the reference accession MG20. Plotting according to IGV [41] default setup. Vertical lines with two colors indicate heterozygous sites. Horizontal bar plots on the right represent the average of root phosphate content under low phosphate conditions of the respective Lotus accessions. The CBR gene model is plotted at the bottom of the figure: exons in black, intron as lines and the direction of transcription indicated by arrows. Two different splicing forms are described in Lotus MG20 annotation. (TIF) [file pgen.1008126.s014.tif]

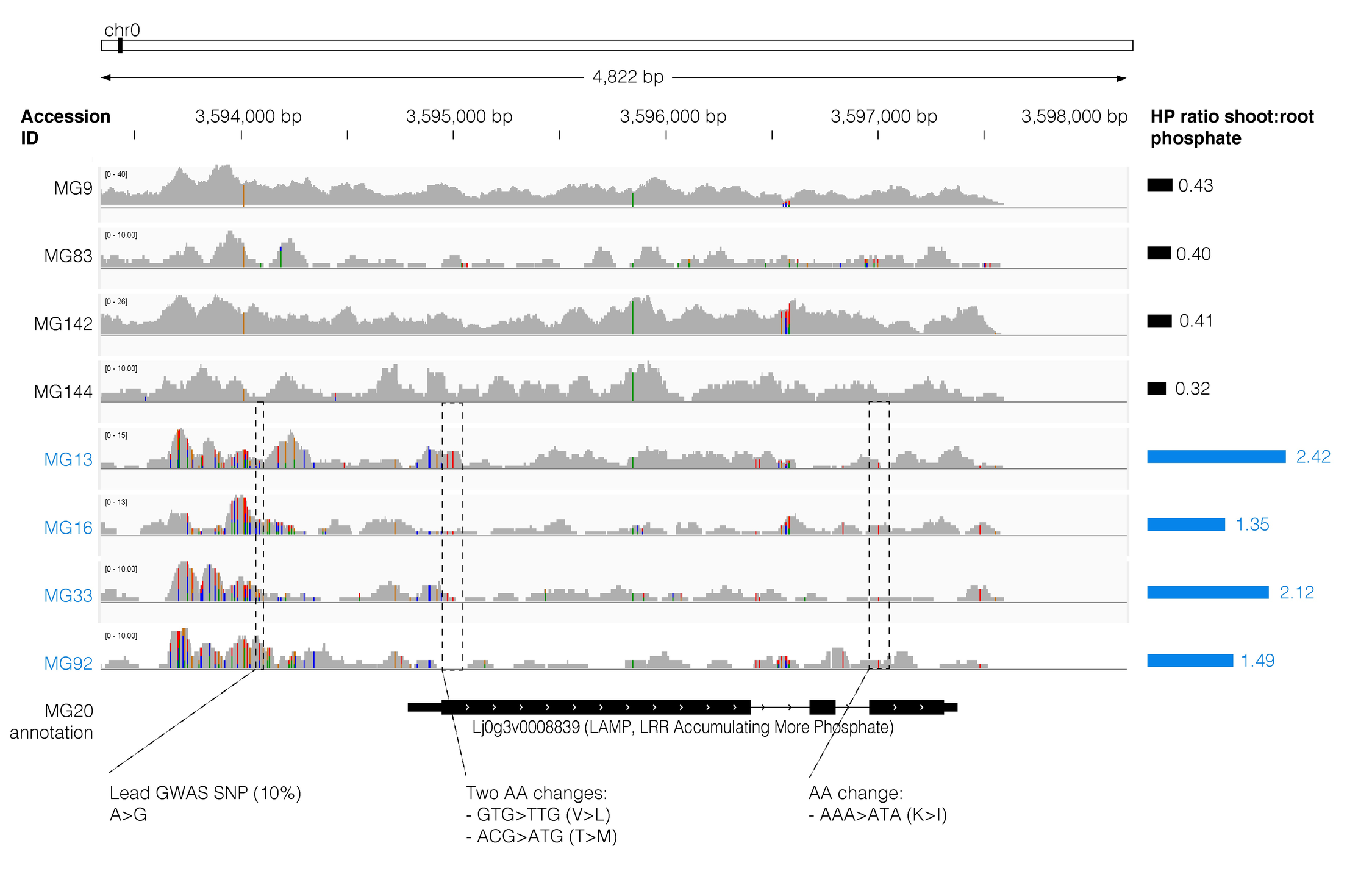

Supplement: S15 Fig — Dashed boxes highlight the shared genetic variation between the depicted accessions accumulating more shoot phosphate compared to the root phosphate content: the lead GWAS SNP (1kb upstream the coding sequence, present in 10% of the Lotus accessions) is within a region containing many SNPs in the predicted promoter region of LAMP. In the beginning of the coding sequence are two SNPs which cause amino acid changes. Towards the end of the coding region, in the putative kinase domain, there is another SNP causing an amino acid change, which is unique for the accessions that show higher values of shoot:root phosphate ratios. Each row visualizes sequence files for a single accession with coverage depth indicated on the y-axis and plotted in gray shades. Different colors represent SNPs compared to the reference accession MG20. Plotting according to IGV [41] default setup. Vertical lines with two colors indicate heterozygous sites. Horizontal bar plots on the right represent the average of shoot:root phosphate ratio root phosphate content under high phosphate conditions of the respective Lotus accessions. The LAMP gene model is plotted at the bottom of the figure: exons in black, intron as lines and the direction of transcription indicated by arrows. (TIF) [file pgen.1008126.s015.tif]
