# Supplementary material for: Identification of novel genes involved in phosphate accumulation in Lotus japonicus through Genome Wide Association mapping of root system architecture and anion content
Source: PLoS Genet. 2019 Dec 19;15(12):e1008126. doi: 10.1371/journal.pgen.1008126 (PMC6941899; doi:10.1371/journal.pgen.1008126)
Supplement: S1 File — (PDF) [file pgen.1008126.s035.pdf]

Lotus root responses to phosphate

Table of Contents

```
library(tidyverse)

## ── Attaching packages ─────────────────────────────────────────────────────────────────────────────────────────────────────────────────────────────────── tidyverse 1.2.1 ──

## ✔ ggplot2 3.1.0     ✔ purrr   0.2.5
## ✔ tibble  1.4.2     ✔ dplyr   0.7.8
## ✔ tidyr   0.8.2     ✔ stringr 1.3.1
## ✔ readr   1.2.1     ✔ forcats 0.3.0

## ── Conflicts ────────────────────────────────────────────────────────────────────────────────────────────────────────────────────────────────────────────── tidyverse_conflicts() ──
## ✖ dplyr::filter() masks stats::filter()
## ✖ dplyr::lag()    masks stats::lag()

###session info
sessionInfo()

## R version 3.5.1 (2018-07-02)
## Platform: x86_64-apple-darwin15.6.0 (64-bit)
## Running under: macOS High Sierra 10.13.6
##
## Matrix products: default
## BLAS:
/Library/Frameworks/R.framework/Versions/3.5/Resources/lib/libRblas.0.dylib
## LAPACK:
/Library/Frameworks/R.framework/Versions/3.5/Resources/lib/libRlapack.dylib
##
## locale:
## [1] en_US.UTF-8/en_US.UTF-8/en_US.UTF-8/C/en_US.UTF-8/en_US.UTF-8
##
## attached base packages:
## [1] stats     graphics  grDevices utils     datasets  methods   base
##
## other attached packages:
## [1] forcats_0.3.0   stringr_1.3.1   dplyr_0.7.8     purrr_0.2.5
## [5] readr_1.2.1     tidyr_0.8.2     tibble_1.4.2    ggplot2_3.1.0
```

```
## [9] tidyverse_1.2.1
##
## loaded via a namespace (and not attached):
##  [1] Rcpp_1.0.0       cellranger_1.1.0 pillar_1.3.0
compiler_3.5.1
##  [5] plyr_1.8.4       bindr_0.1.1      tools_3.5.1
digest_0.6.18
##  [9] lubridate_1.7.4  jsonlite_1.6     evaluate_0.12    nlme_3.1-137
## [13] gtable_0.2.0     lattice_0.20-35  pkgconfig_2.0.2
rlang_0.3.0.1
## [17] cli_1.0.1        rstudioapi_0.8   yaml_2.2.0       haven_2.0.0
## [21] bindrcpp_0.2.2   withr_2.1.2      xml2_1.2.0       httr_1.3.1
## [25] knitr_1.20       hms_0.4.2        generics_0.0.2   grid_3.5.1
## [29] tidyselect_0.2.5 glue_1.3.0       R6_2.3.0         readxl_1.1.0
## [33] rmarkdown_1.11   modelr_0.1.2     magrittr_1.5
backports_1.1.2
## [37] scales_1.0.0     htmltools_0.3.6  rvest_0.3.2
assertthat_0.2.0
## [41] colorspace_1.3-2 stringi_1.2.4    lazyeval_0.2.1
munsell_0.5.0
## [45] broom_0.5.1      crayon_1.3.4
```

```
data <-
read.csv("/Volumes/busch/lab_new/Marco/20180301_draft_manuscript/Lotus_
Brat/20180403_allsets_quant_single_root_anions_edited.csv", header=T,
na.string=c("na", "NA", "null"))
str(data)
```

```
## 'data.frame':    20622 obs. of  36 variables:
##  $ ACC                       : Factor w/ 129 levels
"1","10","100",..: 42 18 39 30 40 128 24 7 111 127 ...
##  $ phosphate                 : Factor w/ 2 levels "20P","750P": 1 1 1
1 1 2 2 1 1 2 ...
##  $ set                       : int  9 12 4 4 4 8 12 3 4 4 ...
##  $ plate                     : int  2 5 8 22 10 9 16 1 2 42 ...
##  $ abs_pos                   : int  3 4 3 3 8 6 1 6 5 2 ...
##  $ row                       : int  1 1 1 1 1 1 1 1 1 1 ...
##  $ col                       : int  1 1 1 1 2 2 1 2 2 1 ...
##  $ day                       : int  0 0 0 0 0 0 0 0 0 0 ...
##  $ phosph_set_acc_pos        : Factor w/ 2608 levels
"LP_1_10_1","LP_1_10_2",..: 1240 292 1007 943 1020 2457 1479 844 1097
2264 ...
##  $ shoot_NO3                 : num  NA NA NA NA NA NA NA NA NA NA ...
##  $ shoot_PO4                 : num  NA NA NA NA NA NA NA NA NA NA ...
##  $ shoot_SO4                 : num  NA NA NA NA NA NA NA NA NA NA ...
##  $ root_NO3                  : num  NA NA NA NA NA NA NA NA NA NA ...
##  $ root_PO4                  : num  NA NA NA NA NA NA NA NA NA NA ...
##  $ root_SO4                  : num  NA NA NA NA NA NA NA NA NA NA ...
##  $ shoot_over_root_NO3       : num  NA NA NA NA NA NA NA NA NA NA ...
##  $ shoot_over_root_PO4       : num  NA NA NA NA NA NA NA NA NA NA ...
```

```
##  $ shoot_over_root_SO4      : num  NA NA NA NA NA NA NA NA NA NA ...
##  $ Core_filename            : Factor w/ 20622 levels
"LP001001001001",..: 5555 7177 3696 4517 3857 13793 16873 2366 3295
12966 ...
##  $ ROI                      : int  3 4 3 3 8 6 1 6 5 2 ...
##  $ Total_length             : num  2.32 11.99 14.25 5.18 9.13 ...
##  $ Euclidian_length         : num  2.08 9.65 13.15 3.03 8.33 ...
##  $ Root_tortuosity          : num  1.11 1.24 1.08 1.71 1.1 ...
##  $ Root_growth_rate         : num  NA NA NA NA NA NA NA NA NA NA ...
##  $ Relative_root_growth_rate: num  NA NA NA NA NA NA NA NA NA NA ...
##  $ Root_angle               : num  -17.146 0.503 -11.889 112.62 -
17.146 ...
##  $ Root_direction_index     : num  0.367 0.498 0.245 0.549 0.352
0.218 0.172 0.196 0.189 0.151 ...
##  $ Root_horizontal_index    : num  0.166 0.696 0.649 0.835 0.76 ...
##  $ Root_vertical_index      : num  0.593 2.883 3.783 0.659 2.31 ...
##  $ Root_linearity           : num  0.739 0.011 0.877 0.481 0.92
0.724 0.867 0.882 0.066 0.444 ...
##  $ Average_root_width       : num  0.889 0.64 0.56 1.082 0.586 ...
##  $ Root_width_20            : num  0.963 0.849 0.562 1.305 0.662 ...
##  $ Root_width_40            : num  0.96 0.649 0.517 0.86 0.568 ...
##  $ Root_width_60            : num  0.83 0.514 0.397 0.905 0.541 ...
##  $ Root_width_80            : num  0.851 0.443 0.61 1.65 0.491 ...
##  $ Root_width_100           : num  0.84 0.745 0.716 0.692 0.666 ...
```

```
summary(data)
```

```
##       ACC        phosphate        set             plate
##  gifu   :  422   20P :10277   Min.   : 1.000   Min.   : 1.00
##  20     :  396   750P:10345   1st Qu.: 4.000   1st Qu.: 8.00
##  18     :  331                Median : 9.000   Median :15.00
##  73     :  327                Mean   : 8.487   Mean   :16.12
##  7      :  286                3rd Qu.:13.000   3rd Qu.:23.00
##  86     :  273                Max.   :17.000   Max.   :46.00
##  (Other):18587
##     abs_pos           row         col             day
##  Min.   :1.000   Min.   :1   Min.   :1.000   Min.   :0.000
##  1st Qu.:3.000   1st Qu.:1   1st Qu.:1.000   1st Qu.:2.000
##  Median :5.000   Median :1   Median :2.000   Median :4.000
##  Mean   :4.556   Mean   :1   Mean   :1.507   Mean   :4.394
##  3rd Qu.:6.000   3rd Qu.:1   3rd Qu.:2.000   3rd Qu.:7.000
##  Max.   :8.000   Max.   :1   Max.   :2.000   Max.   :9.000
##
##    phosph_set_acc_pos   shoot_NO3        shoot_PO4        shoot_SO4
##  MS_4_gifu_2:   20    Min.   : 0.737   Min.   : 0.000   Min.   :
1.465
##  MS_4_gifu_3:   20    1st Qu.:11.762   1st Qu.: 8.943   1st Qu.:
3.845
##  MS_12_42_2 :   19    Median :16.119   Median :11.234   Median :
4.709
```

```
##  MS_12_42_4 :   19    Mean   :17.284   Mean   :11.519   Mean   :
4.817
##  MS_12_42_5 :   19    3rd Qu.:22.214   3rd Qu.:13.895   3rd Qu.:
5.645
##  LP_13_144_3:   18    Max.   :46.437   Max.   :26.646   Max.
:10.367
##  (Other)    :20507    NA's   :19805    NA's   :19805    NA's
:19805
##     root_NO3          root_PO4         root_SO4
shoot_over_root_NO3
##  Min.   :  7.519   Min.   : 0.346   Min.   : 0.526   Min.   :0.016
##  1st Qu.: 36.916   1st Qu.: 5.346   1st Qu.: 1.753   1st Qu.:0.276
##  Median : 44.630   Median :10.935   Median : 2.676   Median :0.363
##  Mean   : 45.017   Mean   :12.152   Mean   : 3.698   Mean   :0.398
##  3rd Qu.: 52.529   3rd Qu.:18.197   3rd Qu.: 4.390   3rd Qu.:0.481
##  Max.   :149.487   Max.   :34.133   Max.   :22.232   Max.   :3.903
##  NA's   :19909     NA's   :19908    NA's   :19911    NA's   :19909
##  shoot_over_root_PO4 shoot_over_root_SO4        Core_filename
##  Min.   : 0.000      Min.   :0.135       LP001001001001:    1
##  1st Qu.: 0.693      1st Qu.:1.023       LP001001001002:    1
##  Median : 1.016      Median :1.749       LP001001001004:    1
##  Mean   : 1.562      Mean   :2.051       LP001001001005:    1
##  3rd Qu.: 1.896      3rd Qu.:2.718       LP001001001006:    1
##  Max.   :13.293      Max.   :8.581       LP001001001007:    1
##  NA's   :19909       NA's   :19911       (Other)       :20616
##       ROI         Total_length    Euclidian_length Root_tortuosity
##  Min.   :1.000   Min.   : 2.237   Min.   : 1.928   Min.   :1.013
##  1st Qu.:3.000   1st Qu.:15.149   1st Qu.:13.895   1st Qu.:1.049
##  Median :5.000   Median :20.921   Median :19.394   Median :1.067
##  Mean   :4.545   Mean   :22.495   Mean   :20.990   Mean   :1.080
##  3rd Qu.:6.000   3rd Qu.:28.334   3rd Qu.:26.551   3rd Qu.:1.094
##  Max.   :8.000   Max.   :73.256   Max.   :69.606   Max.   :3.098
##
##  Root_growth_rate  Relative_root_growth_rate   Root_angle
##  Min.   :-18.995   Min.   :-0.732            Min.   :-71.5650
##  1st Qu.:  1.217   1st Qu.: 0.058            1st Qu.: -4.7672
##  Median :  2.337   Median : 0.106            Median : -0.8175
##  Mean   :  2.409   Mean   : 0.131            Mean   : -0.7145
##  3rd Qu.:  3.507   3rd Qu.: 0.171            3rd Qu.:  3.0570
##  Max.   : 28.825   Max.   : 3.013            Max.   :245.6740
##  NA's   :4892      NA's   :4892
##  Root_direction_index Root_horizontal_index Root_vertical_index
##  Min.   :0.0310       Min.   :0.02115       Min.   : 0.5423
##  1st Qu.:0.1200       1st Qu.:0.39851       1st Qu.: 3.9961
##  Median :0.1690       Median :0.64096       Median : 5.5787
##  Mean   :0.1956       Mean   :0.75117       Mean   : 6.0489
##  3rd Qu.:0.2420       3rd Qu.:0.97851       3rd Qu.: 7.6536
##  Max.   :1.2510       Max.   :4.31347       Max.   :20.0331
##
##  Root_linearity   Average_root_width Root_width_20    Root_width_40
```

```
##  Min.   :0.0000   Min.   :0.2809     Min.   :0.1560   Min.   :0.2518
##  1st Qu.:0.2840   1st Qu.:0.4667     1st Qu.:0.5761   1st Qu.:0.4699
##  Median :0.6320   Median :0.5053     Median :0.6309   Median :0.5129
##  Mean   :0.5555   Mean   :0.5103     Mean   :0.6456   Mean   :0.5240
##  3rd Qu.:0.8260   3rd Qu.:0.5474     3rd Qu.:0.6936   3rd Qu.:0.5623
##  Max.   :0.9980   Max.   :1.1582     Max.   :2.3242   Max.   :2.0080
##                   NA's   :9          NA's   :8        NA's   :4
##  Root_width_60    Root_width_80    Root_width_100
##  Min.   :0.2399   Min.   :0.1722   Min.   :0.1915
##  1st Qu.:0.4387   1st Qu.:0.4088   1st Qu.:0.3413
##  Median :0.4876   Median :0.4703   Median :0.4062
##  Mean   :0.4972   Mean   :0.4774   Mean   :0.4071
##  3rd Qu.:0.5425   3rd Qu.:0.5343   3rd Qu.:0.4613
##  Max.   :1.5634   Max.   :1.7414   Max.   :1.3660
##  NA's   :6        NA's   :4        NA's   :1

data$phosphate <- as.factor(data$phosphate)
data$day <- as.factor(data$day)
data$Root_growth_rate <- as.numeric(data$Root_growth_rate)
data$Relative_root_growth_rate <-
as.numeric(data$Relative_root_growth_rate)

###effect of phosphate * genotype on total length

data %>%
      split(.$day) %>%
      map(
~aov(Total_length ~ phosphate*ACC ,.x) %>%
summary(.)
)

## $`0`
##                   Df Sum Sq Mean Sq F value   Pr(>F)
## phosphate          1    219  219.14  27.646 1.61e-07 ***
## ACC              128  11192   87.43  11.030  < 2e-16 ***
## phosphate:ACC    117   2498   21.35   2.694  < 2e-16 ***
## Residuals       1983  15719    7.93
## ---
## Signif. codes:  0 '***' 0.001 '**' 0.01 '*' 0.05 '.' 0.1 ' ' 1
##
## $`1`
##                   Df Sum Sq Mean Sq F value Pr(>F)
## phosphate          1   1272  1271.8 120.086 <2e-16 ***
## ACC              128  14142   110.5  10.433 <2e-16 ***
## phosphate:ACC    117   3379    28.9   2.727 <2e-16 ***
## Residuals       1961  20768    10.6
## ---
## Signif. codes:  0 '***' 0.001 '**' 0.01 '*' 0.05 '.' 0.1 ' ' 1
##
## $`2`
```

```
##                   Df Sum Sq Mean Sq F value Pr(>F)    
## phosphate          1   2505  2504.7 171.727 <2e-16 ***
## ACC              127  14804   116.6   7.992 <2e-16 ***
## phosphate:ACC  115   4484    39.0   2.673 <2e-16 ***
## Residuals     1827  26648    14.6                   
## ---
## Signif. codes:  0 '***' 0.001 '**' 0.01 '*' 0.05 '.' 0.1 ' ' 1
## 
## $`3`
##                   Df Sum Sq Mean Sq F value   Pr(>F)    
## phosphate          1   2937  2937.0 151.976  < 2e-16 ***
## ACC              127  17570   138.3   7.159  < 2e-16 ***
## phosphate:ACC  116   4644    40.0   2.072 8.54e-10 ***
## Residuals     1751  33839    19.3                     
## ---
## Signif. codes:  0 '***' 0.001 '**' 0.01 '*' 0.05 '.' 0.1 ' ' 1
## 
## $`4`
##                   Df Sum Sq Mean Sq F value   Pr(>F)    
## phosphate          1   3856    3856 143.638  < 2e-16 ***
## ACC              128  23874     187   6.948  < 2e-16 ***
## phosphate:ACC  117   6411      55   2.041 1.52e-09 ***
## Residuals     1862  49984      27                     
## ---
## Signif. codes:  0 '***' 0.001 '**' 0.01 '*' 0.05 '.' 0.1 ' ' 1
## 
## $`5`
##                   Df Sum Sq Mean Sq F value   Pr(>F)    
## phosphate          1   4086    4086 118.546  < 2e-16 ***
## ACC              128  32993     258   7.479  < 2e-16 ***
## phosphate:ACC  118   7995      68   1.966 1.07e-08 ***
## Residuals     1807  62279      34                     
## ---
## Signif. codes:  0 '***' 0.001 '**' 0.01 '*' 0.05 '.' 0.1 ' ' 1
## 
## $`6`
##                   Df Sum Sq Mean Sq F value  Pr(>F)    
## phosphate          1   3048  3048.1  68.790 < 2e-16 ***
## ACC              128  40344   315.2   7.113 < 2e-16 ***
## phosphate:ACC  117   9807    83.8   1.892 8.5e-08 ***
## Residuals     1781  78918    44.3                    
## ---
## Signif. codes:  0 '***' 0.001 '**' 0.01 '*' 0.05 '.' 0.1 ' ' 1
## 
## $`7`
##                   Df Sum Sq Mean Sq F value   Pr(>F)    
## phosphate          1   3329    3329  62.216 5.42e-15 ***
## ACC              128  51640     403   7.541  < 2e-16 ***
## phosphate:ACC  117  11973     102   1.913 5.15e-08 ***
## Residuals     1726  92340      53                     
```

```
## ---
## Signif. codes:  0 '***' 0.001 '**' 0.01 '*' 0.05 '.' 0.1 ' ' 1
##
## $`8`
##                   Df Sum Sq Mean Sq F value   Pr(>F)    
## phosphate          1   1913  1913.3  28.604 1.01e-07 ***
## ACC              128  66745   521.4   7.796  < 2e-16 ***
## phosphate:ACC    118  15354   130.1   1.945 1.98e-08 ***
## Residuals       1725 115385    66.9                     
## ---
## Signif. codes:  0 '***' 0.001 '**' 0.01 '*' 0.05 '.' 0.1 ' ' 1
##
## $`9`
##                   Df Sum Sq Mean Sq F value   Pr(>F)    
## phosphate          1    929   929.0  11.668 0.000651 ***
## ACC              128  80597   629.7   7.908  < 2e-16 ***
## phosphate:ACC    117  19288   164.9   2.071 7.78e-10 ***
## Residuals       1732 137903    79.6                     
## ---
## Signif. codes:  0 '***' 0.001 '**' 0.01 '*' 0.05 '.' 0.1 ' ' 1
```

2-way Anova on Lotus screening

```
###effect of phosphate * genotype on Root width

data %>%
      split(.$day) %>%
      map(
~aov(Root_width_100 ~ phosphate*ACC ,.x) %>%
summary(.)
)

## $`0`
##                   Df Sum Sq  Mean Sq F value   Pr(>F)    
## phosphate          1  0.031 0.031409   9.361 0.002246 ** 
## ACC              128  1.294 0.010107   3.012  < 2e-16 ***
## phosphate:ACC    117  0.586 0.005007   1.492 0.000692 ***
## Residuals       1983  6.654 0.003355                     
## ---
## Signif. codes:  0 '***' 0.001 '**' 0.01 '*' 0.05 '.' 0.1 ' ' 1
##
## $`1`
##                   Df Sum Sq  Mean Sq F value   Pr(>F)    
## phosphate          1  0.009 0.008856   1.971 0.160449    
## ACC              128  1.536 0.012001   2.671  < 2e-16 ***
## phosphate:ACC    117  0.788 0.006737   1.500 0.000602 ***
## Residuals       1961  8.809 0.004492                     
## ---
## Signif. codes:  0 '***' 0.001 '**' 0.01 '*' 0.05 '.' 0.1 ' ' 1
##
## $`2`
```

```
##                 Df Sum Sq Mean Sq F value   Pr(>F)    
## phosphate        1  0.036 0.03569   7.688  0.00562 ** 
## ACC            127  1.650 0.01300   2.799  < 2e-16 ***
## phosphate:ACC  115  1.122 0.00976   2.101 4.07e-10 ***
## Residuals     1827  8.483 0.00464                     
## ---
## Signif. codes:  0 '***' 0.001 '**' 0.01 '*' 0.05 '.' 0.1 ' ' 1
## 
## $`3`
##                 Df Sum Sq Mean Sq F value   Pr(>F)    
## phosphate        1  0.172 0.17239  25.862 4.06e-07 ***
## ACC            127  3.089 0.02432   3.649  < 2e-16 ***
## phosphate:ACC  116  1.574 0.01357   2.036 2.25e-09 ***
## Residuals     1751 11.672 0.00667                     
## ---
## Signif. codes:  0 '***' 0.001 '**' 0.01 '*' 0.05 '.' 0.1 ' ' 1
## 
## $`4`
##                 Df Sum Sq Mean Sq F value   Pr(>F)    
## phosphate        1  0.314 0.31365  34.077 6.23e-09 ***
## ACC            128  3.822 0.02986   3.244  < 2e-16 ***
## phosphate:ACC  117  2.076 0.01775   1.928 3.13e-08 ***
## Residuals     1862 17.138 0.00920                     
## ---
## Signif. codes:  0 '***' 0.001 '**' 0.01 '*' 0.05 '.' 0.1 ' ' 1
## 
## $`5`
##                 Df Sum Sq Mean Sq F value   Pr(>F)    
## phosphate        1  0.267 0.26717  29.351 6.85e-08 ***
## ACC            128  4.940 0.03860   4.240  < 2e-16 ***
## phosphate:ACC  118  2.206 0.01869   2.054 9.95e-10 ***
## Residuals     1807 16.449 0.00910                     
## ---
## Signif. codes:  0 '***' 0.001 '**' 0.01 '*' 0.05 '.' 0.1 ' ' 1
## 
## $`6`
##                 Df Sum Sq Mean Sq F value   Pr(>F)    
## phosphate        1  0.556  0.5563  54.989 1.86e-13 ***
## ACC            128  5.477  0.0428   4.230  < 2e-16 ***
## phosphate:ACC  117  2.385  0.0204   2.015 3.30e-09 ***
## Residuals     1781 18.017  0.0101                     
## ---
## Signif. codes:  0 '***' 0.001 '**' 0.01 '*' 0.05 '.' 0.1 ' ' 1
## 
## $`7`
##                 Df Sum Sq Mean Sq F value   Pr(>F)    
## phosphate        1  0.373  0.3732  36.343 2.02e-09 ***
## ACC            128  5.639  0.0441   4.290  < 2e-16 ***
## phosphate:ACC  117  2.220  0.0190   1.847 2.71e-07 ***
## Residuals     1726 17.726  0.0103                     
```

```
## ---
## Signif. codes:  0 '***' 0.001 '**' 0.01 '*' 0.05 '.' 0.1 ' ' 1
##
## $`8`
##                  Df Sum Sq Mean Sq F value   Pr(>F)    
## phosphate         1  0.328  0.3282  31.613 2.19e-08 ***
## ACC             128  5.685  0.0444   4.279  < 2e-16 ***
## phosphate:ACC   118  2.362  0.0200   1.928 3.11e-08 ***
## Residuals      1724 17.896  0.0104                     
## ---
## Signif. codes:  0 '***' 0.001 '**' 0.01 '*' 0.05 '.' 0.1 ' ' 1
## 1 observation deleted due to missingness
##
## $`9`
##                  Df Sum Sq Mean Sq F value   Pr(>F)    
## phosphate         1  0.420  0.4196  35.146 3.68e-09 ***
## ACC             128  5.597  0.0437   3.662  < 2e-16 ***
## phosphate:ACC   117  2.721  0.0233   1.948 2.08e-08 ***
## Residuals      1732 20.679  0.0119                     
## ---
## Signif. codes:  0 '***' 0.001 '**' 0.01 '*' 0.05 '.' 0.1 ' ' 1
```

Primary root of 130 Lotus accessions (~8 replicates each) grow equally on both low and high phosphate.

Supplementary figure 2a

```
ggplot (data, aes(x=day, y=Total_length, fill=phosphate)) +
      geom_boxplot(outlier.size=0.3, lwd = 0.3) +
      scale_fill_manual(values=c("#bae4bc","#43a2ca")) +
      theme_classic() +
      ggtitle("Primary root length (mm)") +
      theme(plot.title = element_text(hjust = 0.5), axis.title.y =
element_blank())
```

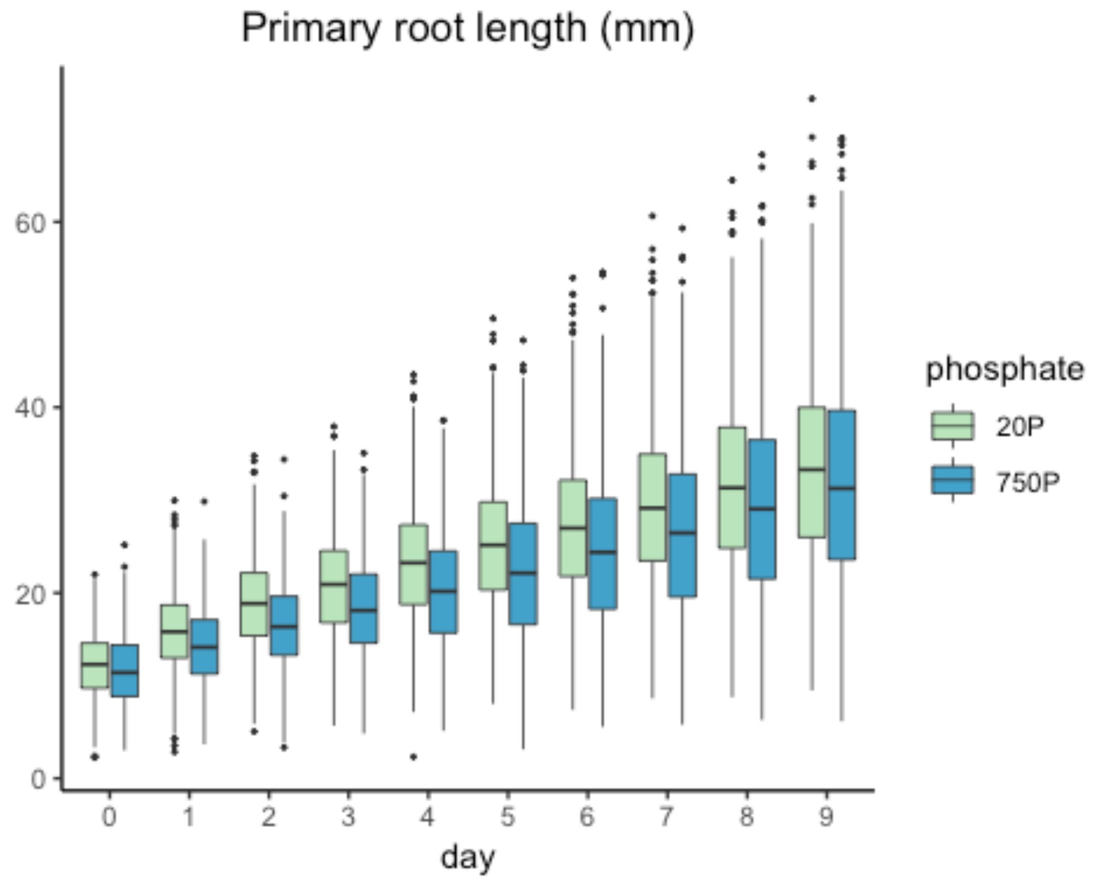

Supplementary figure 2b - Root width 20

```
ggplot (data, aes(x=day, y=Root_width_20, fill=phosphate)) +
      geom_boxplot(outlier.size=0.3, lwd = 0.3)    +
      scale_y_continuous(limits = c(0.4, 0.9)) +
      scale_fill_manual(values=c("#bae4bc","#43a2ca")) +
      theme_classic()+ ggtitle("Root_20 width (mm)") +
      theme(plot.title = element_text(hjust = 0.5), axis.title.y =
element_blank())

## Warning: Removed 506 rows containing non-finite values
(stat_boxplot).
```

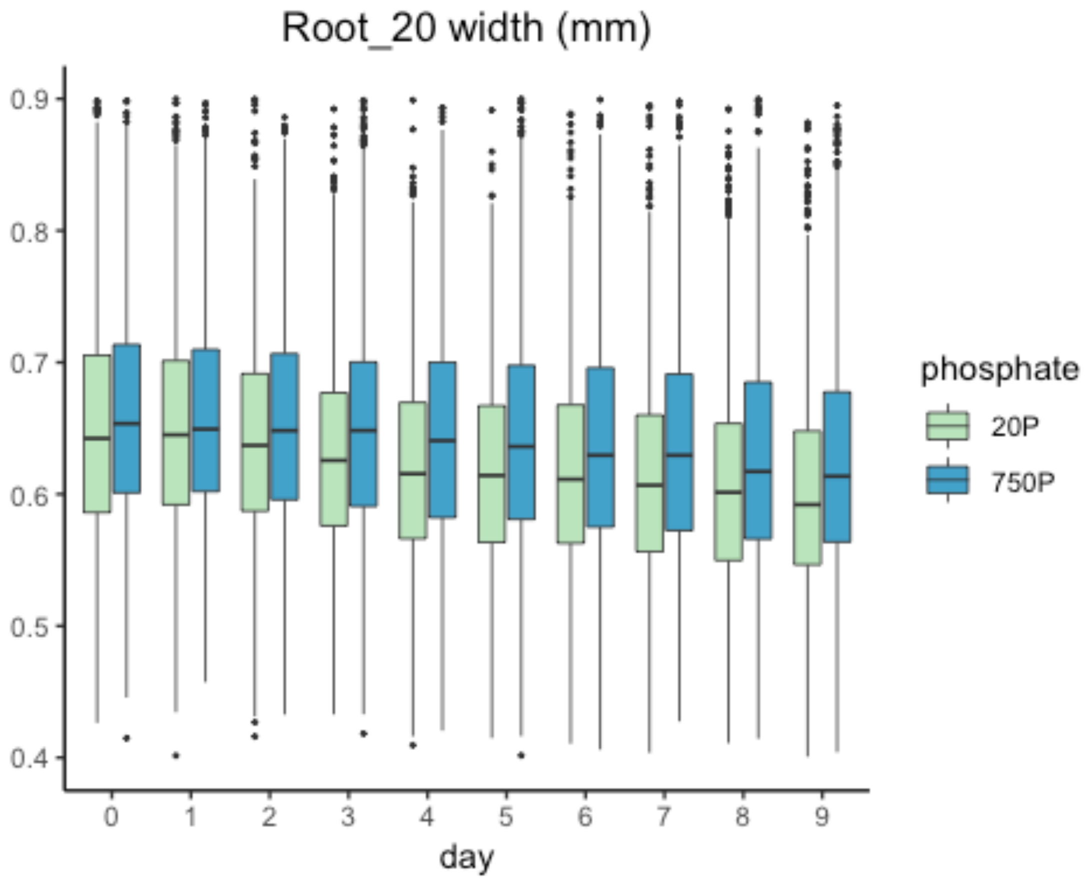

Supplementary figure 2c - Root width 100

```
ggplot (data, aes(x=day, y=Root_width_100, fill=phosphate)) +
      geom_boxplot(outlier.size=0.3, lwd = 0.3)    +
      scale_y_continuous(limits = c(0.2, 0.75)) +
      scale_fill_manual(values=c("#bae4bc","#43a2ca")) +
      theme_classic(base_size=16)+
      ggtitle("Root_100 width (mm)") +
      theme(plot.title = element_text(hjust = 0.5), axis.title.y =
element_blank())

## Warning: Removed 90 rows containing non-finite values
(stat_boxplot).
```

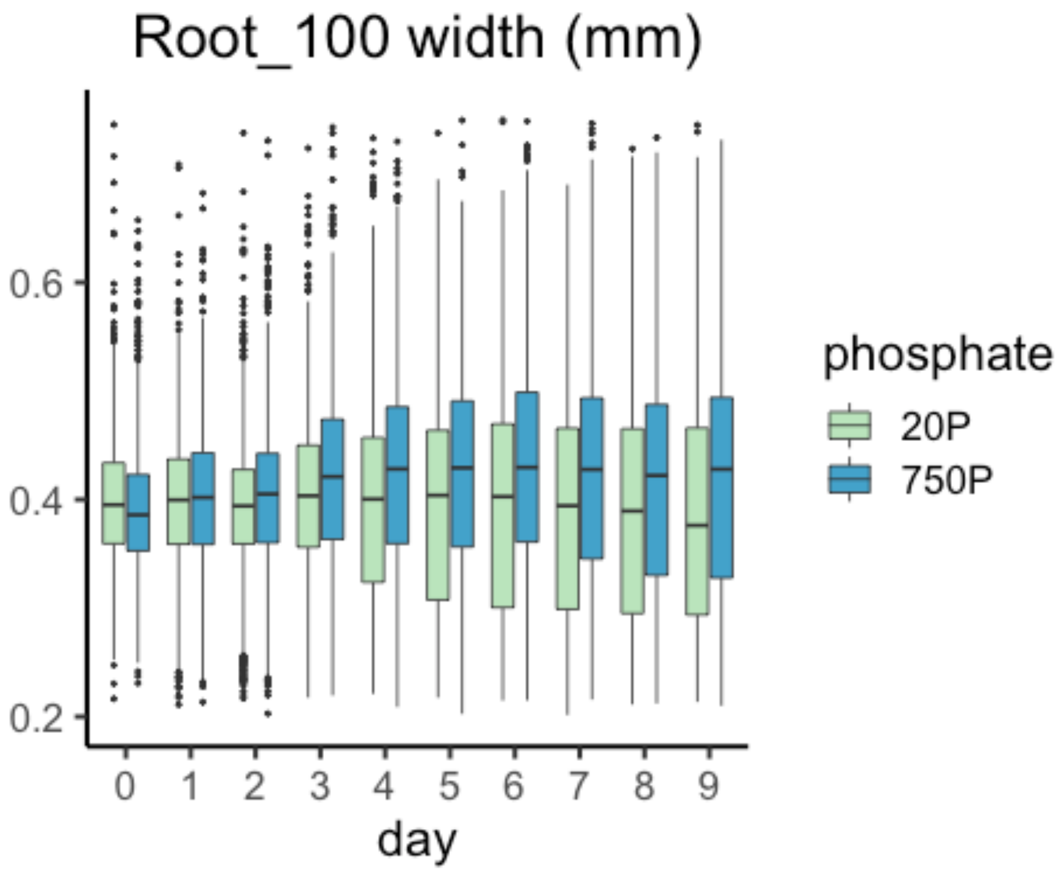

Figure 1 b, c - plotting nutrient distribution

```
test <- data %>%
      select(1:15) %>%
      gather(key=trait, value="value",colnames(.[10:ncol(.)]))
test$trait_f = factor(test$trait,
levels=c('shoot_NO3','shoot_SO4','shoot_PO4','root_NO3','root_SO4','roo
t_PO4'))

test %>% ggplot(aes(x=phosphate, y=value, fill=phosphate)) +
      geom_boxplot(outlier.size=0.3, lwd = 0.3)   +
      scale_fill_manual(values=c("#bae4bc","#43a2ca"))    +
      xlab("P media")    +
      theme_classic() +
      facet_wrap(~trait_f, scales="free") +
      ggtitle("Anions' concentration (ng/mg)") +
      theme(plot.title = element_text(hjust = 0.5), axis.title.y =
element_blank())

## Warning: Removed 119143 rows containing non-finite values
(stat_boxplot).
```

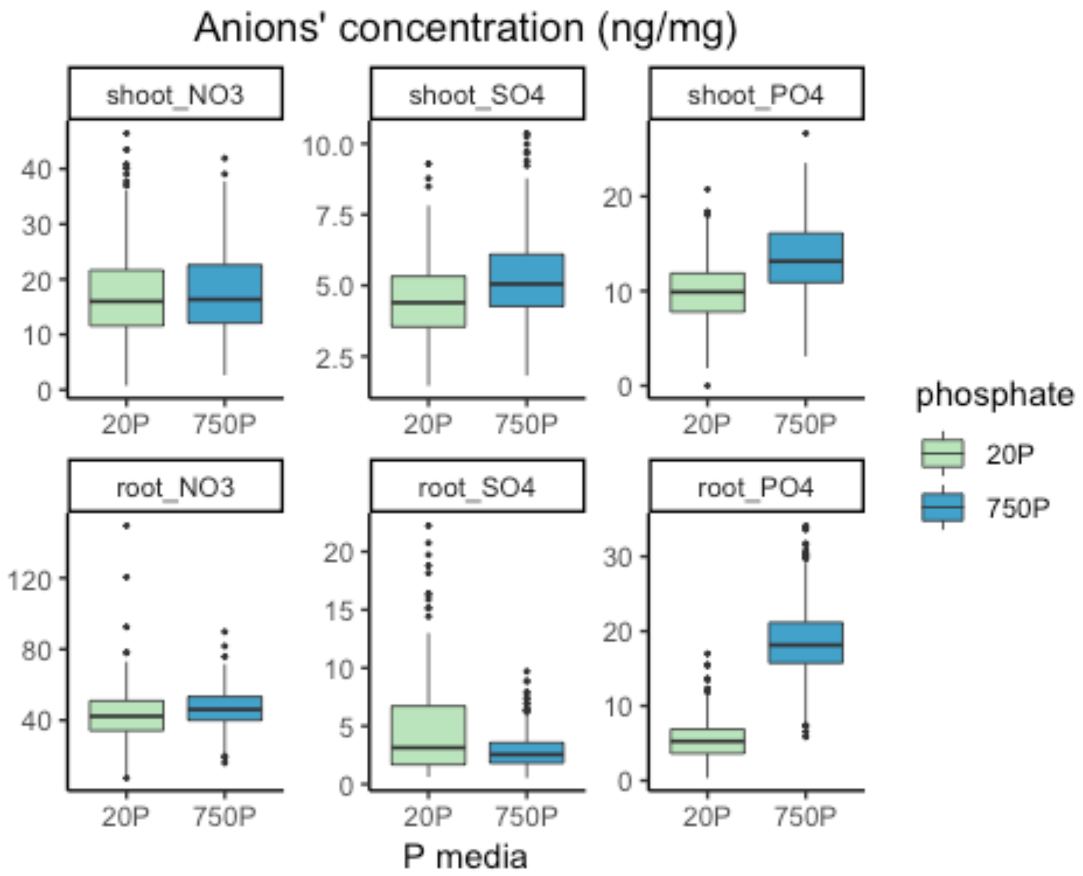

Figure 2 b - Correlation between root traits and anions' uptake on low phosphate

```
library(corrplot)

## corrplot 0.84 loaded

data %>% filter (data$day=="9" & data$phosphate == "20P") ->
data_day9_LP
corr_data_root_anions_trait_day9_LP <- data_day9_LP [, c("shoot_NO3",
"shoot_PO4", "shoot_SO4","root_NO3", "root_PO4", "root_SO4",
"shoot_over_root_NO3", "shoot_over_root_PO4", "shoot_over_root_SO4",
"Total_length", "Euclidian_length" , "Root_tortuosity",
"Root_growth_rate"    , "Relative_root_growth_rate", "Root_angle",
"Root_direction_index" ,"Root_horizontal_index" ,
"Root_vertical_index","Root_linearity", "Average_root_width" ,
"Root_width_20","Root_width_40","Root_width_60",
"Root_width_80","Root_width_100" )]
corr_data_root_anions_trait_day9_LP$shoot_SO4 <-
as.numeric(corr_data_root_anions_trait_day9_LP$shoot_SO4)
library(corrplot)
#data <-
read.csv("/Volumes/busch/lab_new/Marco/Lotus_nutrient/LP_acc_phenotypes
```

```
_reduced.csv", header=T, na.string=c("na", "NA"))
M <- cor(corr_data_root_anions_trait_day9_LP,
use='pairwise.complete.obs')
M
```

```
##                              shoot_NO3   shoot_PO4    shoot_SO4
## shoot_NO3                  1.000000000 -0.06426749  0.312174769
## shoot_PO4                 -0.064267494  1.00000000  0.114654454
## shoot_SO4                  0.312174769  0.11465445  1.000000000
## root_NO3                   0.287180791  0.08570203  0.079919257
## root_PO4                   0.127105759  0.05793829  0.133607026
## root_SO4                  -0.029413937 -0.12841550 -0.090941567
## shoot_over_root_NO3        0.545677990 -0.09996607  0.100247311
## shoot_over_root_PO4        0.002246994  0.37867139 -0.004669462
## shoot_over_root_SO4        0.175885039  0.20121060  0.451529300
## Total_length              -0.076206818 -0.08499683 -0.208647019
## Euclidian_length          -0.069052127 -0.07881851 -0.202972627
## Root_tortuosity           -0.043625225 -0.04820607  0.028169340
## Root_growth_rate          -0.088652400 -0.05611817 -0.078752474
## Relative_root_growth_rate -0.048228968 -0.03779344 -0.051116169
## Root_angle                 0.011945425 -0.09641452  0.003645679
## Root_direction_index      -0.054165810 -0.08223129  0.063918978
## Root_horizontal_index     -0.077727808 -0.09365331 -0.054599345
## Root_vertical_index       -0.067215851 -0.07514166 -0.205155672
## Root_linearity            -0.015056671  0.01518155 -0.057637237
## Average_root_width         0.079232021 -0.03735733  0.075188318
## Root_width_20              0.041012246 -0.01215688 -0.081886424
## Root_width_40              0.018272646  0.06250664 -0.105087692
## Root_width_60              0.103941642 -0.01918100  0.111402137
## Root_width_80              0.094986846 -0.03742209  0.168001892
## Root_width_100             0.039339394 -0.06987935  0.160499954
##                               root_NO3    root_PO4     root_SO4
## shoot_NO3                  0.287180791  0.127105759 -0.029413937
## shoot_PO4                  0.085702034  0.057938286 -0.128415503
## shoot_SO4                  0.079919257  0.133607026 -0.090941567
## root_NO3                   1.000000000  0.165618725 -0.143905500
## root_PO4                   0.165618725  1.000000000 -0.069976392
## root_SO4                  -0.143905500 -0.069976392  1.000000000
## shoot_over_root_NO3       -0.338962221 -0.047007925  0.071952725
## shoot_over_root_PO4       -0.020237657 -0.668260229  0.002552096
## shoot_over_root_SO4        0.198869017  0.140456732 -0.660551292
## Total_length              -0.005534831 -0.115912370  0.311223666
## Euclidian_length          -0.005603130 -0.120240582  0.311469238
## Root_tortuosity            0.008818767  0.117610127 -0.133434149
## Root_growth_rate          -0.058703874 -0.070791998  0.197650883
## Relative_root_growth_rate -0.060173602 -0.056257815  0.124245931
## Root_angle                 0.011628363  0.009549397 -0.073263196
## Root_direction_index      -0.018690035  0.130818765 -0.118149201
## Root_horizontal_index     -0.037746380  0.046927057  0.013309193
## Root_vertical_index       -0.002386468 -0.122465087  0.310516209
```

```
## Root_linearity             0.058155284 -0.024837051 -0.086179599
## Average_root_width         0.044847190  0.086155631 -0.113939899
## Root_width_20              0.049501362  0.042601225 -0.109876294
## Root_width_40              0.002013098 -0.055076525  0.026574160
## Root_width_60              0.046130716  0.094974842 -0.046287009
## Root_width_80              0.028543173  0.121671680 -0.101628039
## Root_width_100             0.049333200  0.125200331 -0.084663233
##                           shoot_over_root_NO3 shoot_over_root_PO4
## shoot_NO3                        0.5456779896         0.002246994
## shoot_PO4                       -0.0999660749         0.378671387
## shoot_SO4                        0.1002473109        -0.004669462
## root_NO3                        -0.3389622209        -0.020237657
## root_PO4                        -0.0470079251        -0.668260229
## root_SO4                         0.0719527254         0.002552096
## shoot_over_root_NO3              1.0000000000         0.066093010
## shoot_over_root_PO4              0.0660930103         1.000000000
## shoot_over_root_SO4             -0.0297045998         0.007356765
## Total_length                    -0.0020486870         0.008004971
## Euclidian_length                 0.0066999013         0.016466634
## Root_tortuosity                 -0.1003693167        -0.112466150
## Root_growth_rate                -0.0385217973         0.017362434
## Relative_root_growth_rate       -0.0264002879         0.025472124
## Root_angle                      -0.0039112239        -0.007167795
## Root_direction_index            -0.1036846058        -0.144625172
## Root_horizontal_index           -0.0929272516        -0.077574408
## Root_vertical_index              0.0077319331         0.019941944
## Root_linearity                  -0.0595187145         0.078168338
## Average_root_width               0.0067034487        -0.026374926
## Root_width_20                    0.0131469626        -0.036689331
## Root_width_40                    0.0007361729         0.071599137
## Root_width_60                   -0.0014672377        -0.040876430
## Root_width_80                    0.0429473889        -0.027508730
## Root_width_100                  -0.0046582865        -0.078224605
##                           shoot_over_root_SO4 Total_length
## shoot_NO3                         0.175885039 -0.076206818
## shoot_PO4                         0.201210602 -0.084996830
## shoot_SO4                         0.451529300 -0.208647019
## root_NO3                          0.198869017 -0.005534831
## root_PO4                          0.140456732 -0.115912370
## root_SO4                         -0.660551292  0.311223666
## shoot_over_root_NO3              -0.029704600 -0.002048687
## shoot_over_root_PO4               0.007356765  0.008004971
## shoot_over_root_SO4               1.000000000 -0.302627202
## Total_length                     -0.302627202  1.000000000
## Euclidian_length                 -0.301170172  0.991806192
## Root_tortuosity                   0.099394762 -0.207853195
## Root_growth_rate                 -0.204675893  0.547398727
## Relative_root_growth_rate        -0.146969661  0.254226526
## Root_angle                        0.068264291 -0.096524655
## Root_direction_index              0.086893439 -0.365091715
```

```
## Root_horizontal_index           -0.035574998  0.153903659
## Root_vertical_index             -0.300521196  0.994603361
## Root_linearity                   0.009548851 -0.067151772
## Average_root_width               0.091422541 -0.288589614
## Root_width_20                   -0.010191994 -0.171634438
## Root_width_40                   -0.032131878  0.030583645
## Root_width_60                    0.064752781 -0.177704631
## Root_width_80                    0.104520189 -0.305175364
## Root_width_100                   0.105440872 -0.227299701
##                            Euclidian_length Root_tortuosity
## shoot_NO3                      -0.069052127    -0.043625225
## shoot_PO4                      -0.078818508    -0.048206069
## shoot_SO4                      -0.202972627     0.028169340
## root_NO3                       -0.005603130     0.008818767
## root_PO4                       -0.120240582     0.117610127
## root_SO4                        0.311469238    -0.133434149
## shoot_over_root_NO3             0.006699901    -0.100369317
## shoot_over_root_PO4             0.016466634    -0.112466150
## shoot_over_root_SO4            -0.301170172     0.099394762
## Total_length                    0.991806192    -0.207853195
## Euclidian_length                1.000000000    -0.313486414
## Root_tortuosity                -0.313486414     1.000000000
## Root_growth_rate                0.547949519    -0.140449479
## Relative_root_growth_rate       0.257421107    -0.110326978
## Root_angle                     -0.117902157     0.534916436
## Root_direction_index           -0.446164461     0.737308345
## Root_horizontal_index           0.080563686     0.423012797
## Root_vertical_index             0.998689839    -0.289589001
## Root_linearity                 -0.056150477    -0.088157232
## Average_root_width             -0.310891436     0.312018962
## Root_width_20                  -0.178441526     0.106701984
## Root_width_40                   0.021915929     0.088301294
## Root_width_60                  -0.183530431     0.100096940
## Root_width_80                  -0.321850140     0.227848576
## Root_width_100                 -0.254423331     0.355555667
##                            Root_growth_rate Relative_root_growth_rate
## shoot_NO3                       -0.08865240              -0.048228968
## shoot_PO4                       -0.05611817              -0.037793443
## shoot_SO4                       -0.07875247              -0.051116169
## root_NO3                        -0.05870387              -0.060173602
## root_PO4                        -0.07079200              -0.056257815
## root_SO4                         0.19765088               0.124245931
## shoot_over_root_NO3             -0.03852180              -0.026400288
## shoot_over_root_PO4              0.01736243               0.025472124
## shoot_over_root_SO4             -0.20467589              -0.146969661
## Total_length                     0.54739873               0.254226526
## Euclidian_length                 0.54794952               0.257421107
## Root_tortuosity                 -0.14044948              -0.110326978
## Root_growth_rate                 1.00000000               0.885734185
## Relative_root_growth_rate        0.88573418               1.000000000
```

```
## Root_angle                         -0.06538031            -0.071585816
## Root_direction_index               -0.22370045            -0.149581195
## Root_horizontal_index               0.03613139            -0.018348152
## Root_vertical_index                 0.54929524             0.259628204
## Root_linearity                      0.00534774             0.044519433
## Average_root_width                 -0.14900029            -0.059296123
## Root_width_20                      -0.06270315             0.005656068
## Root_width_40                       0.01152250             0.035496002
## Root_width_60                      -0.05388337             0.049887280
## Root_width_80                      -0.13674116            -0.055260381
## Root_width_100                     -0.21498853            -0.238791934
##                              Root_angle Root_direction_index
## shoot_NO3                    0.011945425          -0.05416581
## shoot_PO4                   -0.096414519          -0.08223129
## shoot_SO4                    0.003645679           0.06391898
## root_NO3                     0.011628363          -0.01869003
## root_PO4                     0.009549397           0.13081877
## root_SO4                    -0.073263196          -0.11814920
## shoot_over_root_NO3         -0.003911224          -0.10368461
## shoot_over_root_PO4         -0.007167795          -0.14462517
## shoot_over_root_SO4          0.068264291           0.08689344
## Total_length                -0.096524655          -0.36509171
## Euclidian_length            -0.117902157          -0.44616446
## Root_tortuosity              0.534916436           0.73730834
## Root_growth_rate            -0.065380312          -0.22370045
## Relative_root_growth_rate -0.071585816          -0.14958120
## Root_angle                   1.000000000           0.20129514
## Root_direction_index         0.201295143           1.00000000
## Root_horizontal_index        0.058940850           0.60423175
## Root_vertical_index         -0.116205998          -0.44531170
## Root_linearity              -0.021751761           0.02123393
## Average_root_width           0.246466880           0.25648822
## Root_width_20                0.032288845           0.11992866
## Root_width_40                0.091347460          -0.02864083
## Root_width_60                0.110273032           0.07669279
## Root_width_80                0.130911943           0.23300439
## Root_width_100               0.302324267           0.29068078
##                           Root_horizontal_index Root_vertical_index
## shoot_NO3                         -0.0777278085        -0.067215851
## shoot_PO4                         -0.0936533102        -0.075141663
## shoot_SO4                         -0.0545993452        -0.205155672
## root_NO3                          -0.0377463795        -0.002386468
## root_PO4                           0.0469270573        -0.122465087
## root_SO4                           0.0133091928         0.310516209
## shoot_over_root_NO3               -0.0929272516         0.007731933
## shoot_over_root_PO4               -0.0775744077         0.019941944
## shoot_over_root_SO4               -0.0355749975        -0.300521196
## Total_length                       0.1539036591         0.994603361
## Euclidian_length                   0.0805636857         0.998689839
## Root_tortuosity                    0.4230127971        -0.289589001
```

```
## Root_growth_rate                0.0361313863         0.549295239
## Relative_root_growth_rate      -0.0183481525         0.259628204
## Root_angle                      0.0589408504        -0.116205998
## Root_direction_index            0.6042317508        -0.445311697
## Root_horizontal_index           1.0000000000         0.074032208
## Root_vertical_index             0.0740322080         1.000000000
## Root_linearity                  0.4014199619        -0.067714569
## Average_root_width             -0.0010844819        -0.303664247
## Root_width_20                  -0.0351162343        -0.174349067
## Root_width_40                  -0.0334198784         0.028015601
## Root_width_60                   0.0006063623        -0.182256328
## Root_width_80                   0.0056682231        -0.318615789
## Root_width_100                  0.0458883677        -0.247668575
##                             Root_linearity Average_root_width
Root_width_20
## shoot_NO3                     -0.015056671        0.079232021
0.0410122457
## shoot_PO4                      0.015181546       -0.037357333 -
0.0121568819
## shoot_SO4                     -0.057637237        0.075188318 -
0.0818864240
## root_NO3                       0.058155284        0.044847190
0.0495013619
## root_PO4                      -0.024837051        0.086155631
0.0426012248
## root_SO4                      -0.086179599       -0.113939899 -
0.1098762935
## shoot_over_root_NO3           -0.059518715        0.006703449
0.0131469626
## shoot_over_root_PO4            0.078168338       -0.026374926 -
0.0366893309
## shoot_over_root_SO4            0.009548851        0.091422541 -
0.0101919938
## Total_length                  -0.067151772       -0.288589614 -
0.1716344377
## Euclidian_length              -0.056150477       -0.310891436 -
0.1784415264
## Root_tortuosity               -0.088157232        0.312018962
0.1067019843
## Root_growth_rate               0.005347740       -0.149000287 -
0.0627031484
## Relative_root_growth_rate      0.044519433       -0.059296123
0.0056560684
## Root_angle                    -0.021751761        0.246466880
0.0322888452
## Root_direction_index           0.021233934        0.256488216
0.1199286574
## Root_horizontal_index          0.401419962       -0.001084482 -
0.0351162343
## Root_vertical_index           -0.067714569       -0.303664247 -
```

```
0.1743490672
## Root_linearity               1.000000000       -0.031008703 -
0.0107725918
## Average_root_width          -0.031008703        1.000000000
0.4489106603
## Root_width_20               -0.010772592        0.448910660
1.0000000000
## Root_width_40               -0.014245567        0.470305561
0.4111176806
## Root_width_60                0.008970569        0.699320171
0.0004417725
## Root_width_80               -0.030338985        0.776858377
0.0489900761
## Root_width_100              -0.035952629        0.652515882
0.0270300891
##                            Root_width_40 Root_width_60 Root_width_80
## shoot_NO3                    0.0182726464  0.1039416418   0.094986846
## shoot_PO4                    0.0625066371 -0.0191810004  -0.037422093
## shoot_SO4                   -0.1050876916  0.1114021368   0.168001892
## root_NO3                     0.0020130975  0.0461307163   0.028543173
## root_PO4                    -0.0550765249  0.0949748421   0.121671680
## root_SO4                     0.0265741601 -0.0462870087  -0.101628039
## shoot_over_root_NO3          0.0007361729 -0.0014672377   0.042947389
## shoot_over_root_PO4          0.0715991366 -0.0408764298  -0.027508730
## shoot_over_root_SO4         -0.0321318779  0.0647527808   0.104520189
## Total_length                 0.0305836453 -0.1777046311  -0.305175364
## Euclidian_length             0.0219159287 -0.1835304310  -0.321850140
## Root_tortuosity              0.0883012940  0.1000969396   0.227848576
## Root_growth_rate             0.0115225007 -0.0538833678  -0.136741156
## Relative_root_growth_rate  0.0354960021  0.0498872799  -0.055260381
## Root_angle                   0.0913474605  0.1102730321   0.130911943
## Root_direction_index        -0.0286408265  0.0766927940   0.233004393
## Root_horizontal_index       -0.0334198784  0.0006063623   0.005668223
## Root_vertical_index          0.0280156013 -0.1822563280  -0.318615789
## Root_linearity              -0.0142455665  0.0089705686  -0.030338985
## Average_root_width           0.4703055607  0.6993201711   0.776858377
## Root_width_20                0.4111176806  0.0004417725   0.048990076
## Root_width_40                1.0000000000  0.2595295063   0.024896389
## Root_width_60                0.2595295063  1.0000000000   0.621309478
## Root_width_80                0.0248963889  0.6213094782   1.000000000
## Root_width_100               0.0225423067  0.2888449614   0.668232075
##                            Root_width_100
## shoot_NO3                     0.039339394
## shoot_PO4                    -0.069879353
## shoot_SO4                     0.160499954
## root_NO3                      0.049333200
## root_PO4                      0.125200331
## root_SO4                     -0.084663233
## shoot_over_root_NO3          -0.004658286
## shoot_over_root_PO4          -0.078224605
```

```
## shoot_over_root_SO4           0.105440872
## Total_length                 -0.227299701
## Euclidian_length             -0.254423331
## Root_tortuosity               0.355555667
## Root_growth_rate             -0.214988526
## Relative_root_growth_rate    -0.238791934
## Root_angle                    0.302324267
## Root_direction_index          0.290680775
## Root_horizontal_index         0.045888368
## Root_vertical_index          -0.247668575
## Root_linearity               -0.035952629
## Average_root_width            0.652515882
## Root_width_20                 0.027030089
## Root_width_40                 0.022542307
## Root_width_60                 0.288844961
## Root_width_80                 0.668232075
## Root_width_100                1.000000000

write.csv (M, file=
"/Volumes/busch/lab_new/Marco/20180301_draft_manuscript/correlation/LP_
corr_data_root_trait_matrix.csv")

corrplot(M, method="color", type="upper", diag=FALSE,tl.col="black",
tl.srt=45, tl.cex=0.8,  sig.level=0.05)
```

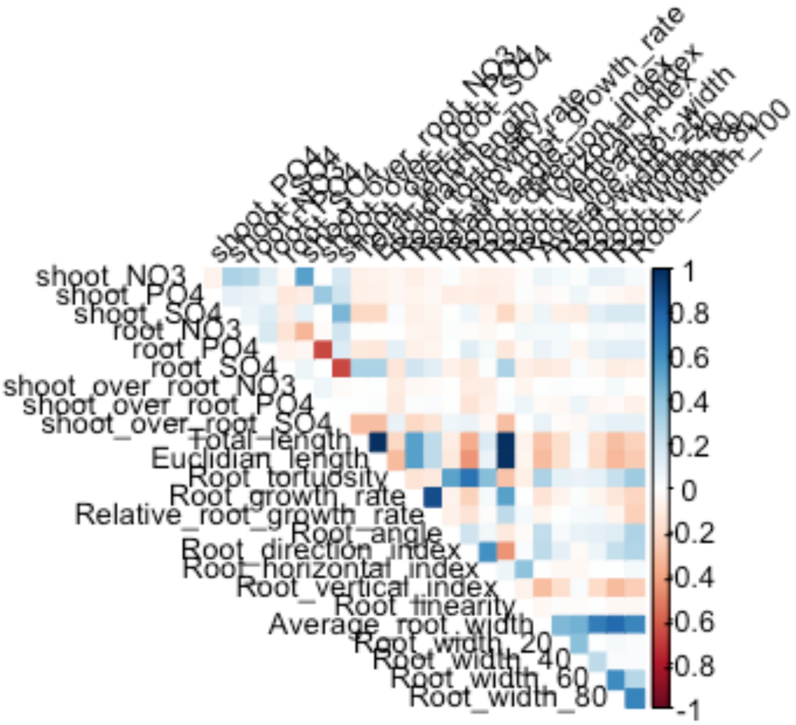

Correlation between root traits and anions' uptake on high phosphate

Figure 2 b

```
library("corrplot")
data %>% filter (data$day=="9" & data$phosphate == "750P") ->
data_day9_HP
data_day9_HP$phosphate <-factor (data_day9_HP$phosphate)
corr_data_root_anions_trait_day9_HP <- data_day9_HP [, c("shoot_NO3",
"shoot_PO4", "shoot_SO4","root_NO3", "root_PO4", "root_SO4",
"shoot_over_root_NO3", "shoot_over_root_PO4", "shoot_over_root_SO4",
"Total_length", "Euclidian_length" , "Root_tortuosity",
"Root_growth_rate"   , "Relative_root_growth_rate", "Root_angle",
"Root_direction_index" ,"Root_horizontal_index" ,
"Root_vertical_index","Root_linearity", "Average_root_width" ,
"Root_width_20","Root_width_40","Root_width_60",
"Root_width_80","Root_width_100" )]
corr_data_root_anions_trait_day9_HP$shoot_SO4 <-
as.numeric(corr_data_root_anions_trait_day9_HP$shoot_SO4)
library(corrplot)
#data <-
read.csv("/Volumes/busch/lab_new/Marco/Lotus_nutrient/LP_acc_phenotypes
```

```
_reduced.csv", header=T, na.string=c("na", "NA"))
M_HP <- cor(corr_data_root_anions_trait_day9_HP,
use='pairwise.complete.obs')
write.csv (M_HP, file=
"/Volumes/busch/lab_new/Marco/20180301_draft_manuscript/correlation/HP_
corr_data_root_trait_matrix.csv")

corrplot(M_HP, method="color", type="upper",
diag=FALSE,tl.col="black", tl.srt=45, tl.cex=0.8,  sig.level=0.05)
```

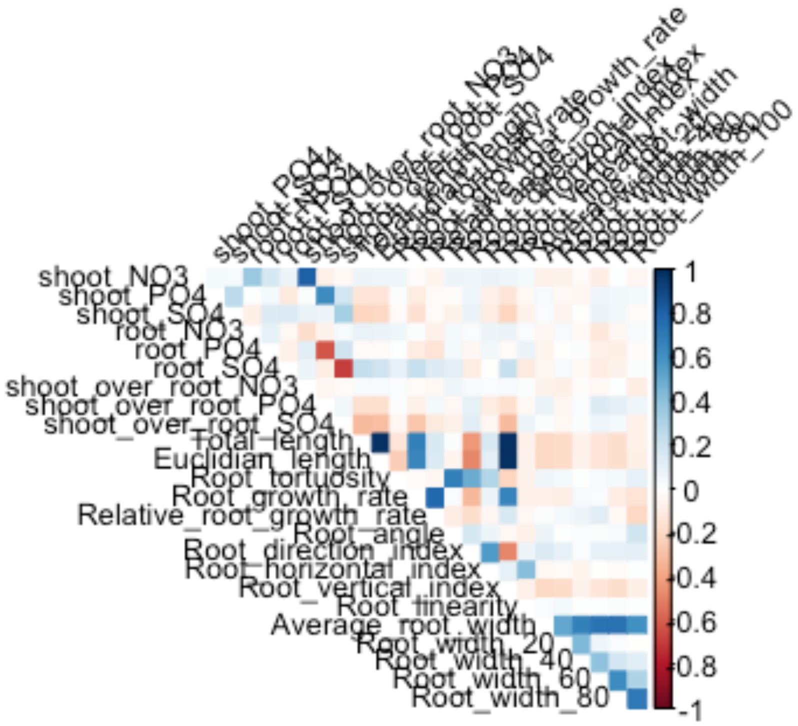

Supplementary figure 3 - Significance correlation among total root length and root phosphate accumulation only happen under low phosphate

```
data %>% filter (data$day=="9") -> data_day9
#data_day9_LP$day <-factor (data_day9_LP$day)
ggplot (data_day9, aes(x=root_PO4, y=Total_length, color=phosphate)) +
      geom_point(size=0.9) +
      scale_colour_manual(values=c("#bae4bc","#43a2ca")) +
      geom_smooth(method="lm", se=F) +
      theme_classic() +
      facet_wrap (~phosphate)
```

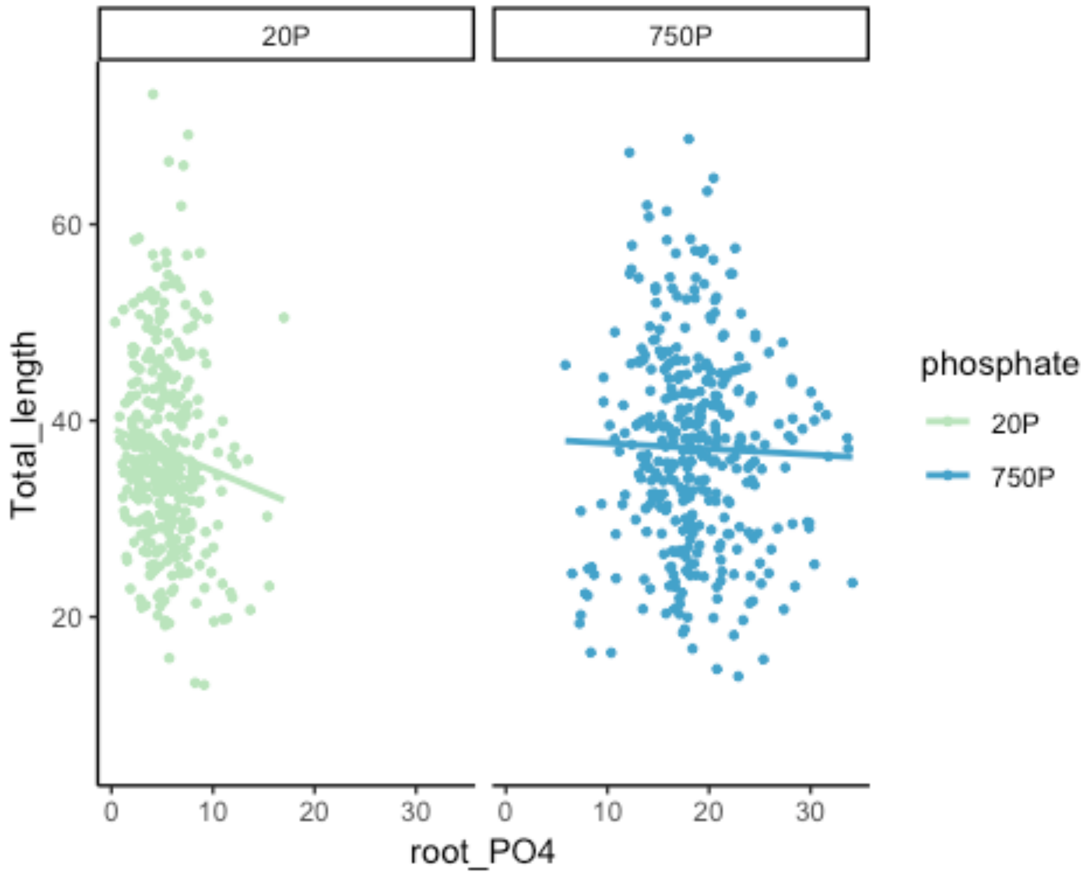


```
data %>% filter (data$day=="9" & phosphate == "20P") -> data_day9_LP
cor.test(data_day9_LP$shoot_over_root_PO4, data_day9_LP$Total_length,
na.omit=TRUE)

##
##  Pearson's product-moment correlation
##
## data:  data_day9_LP$shoot_over_root_PO4 and
data_day9_LP$Total_length
## t = 0.14912, df = 347, p-value = 0.8815
## alternative hypothesis: true correlation is not equal to 0
## 95 percent confidence interval:
##  -0.09705668  0.11289019
## sample estimates:
##         cor
## 0.008004971

data %>% filter (data$day=="9" & phosphate == "750P") -> data_day9_HP
cor.test(data_day9_HP$shoot_over_root_PO4, data_day9_HP$Total_length,
na.omit=TRUE)

##
##  Pearson's product-moment correlation
##
```

```
## data:  data_day9_HP$shoot_over_root_PO4 and
data_day9_HP$Total_length
## t = -3.2449, df = 362, p-value = 0.001284
## alternative hypothesis: true correlation is not equal to 0
## 95 percent confidence interval:
##  -0.26631165 -0.06647951
## sample estimates:
##        cor
## -0.1681223

data %>% filter (data$day=="9" & phosphate == "750P") -> data_day9_HP
cor.test(data_day9_HP$shoot_over_root_PO4, data_day9_HP$Root_width_60,
na.omit=TRUE)

##
##  Pearson's product-moment correlation
##
## data:  data_day9_HP$shoot_over_root_PO4 and
data_day9_HP$Root_width_60
## t = 2.7758, df = 362, p-value = 0.005793
## alternative hypothesis: true correlation is not equal to 0
## 95 percent confidence interval:
##  0.04219718 0.24354035
## sample estimates:
##       cor
## 0.1443627

data %>% filter (data$day=="9" & phosphate == "20P") -> data_day9_LP
cor.test(data_day9_LP$shoot_over_root_PO4, data_day9_LP$Root_width_60,
na.omit=TRUE)

##
##  Pearson's product-moment correlation
##
## data:  data_day9_LP$shoot_over_root_PO4 and
data_day9_LP$Root_width_60
## t = -0.76098, df = 346, p-value = 0.4472
## alternative hypothesis: true correlation is not equal to 0
## 95 percent confidence interval:
##  -0.14538267  0.06453189
## sample estimates:
##         cor
## -0.04087643

data %>% filter (data$day=="9" & phosphate == "20P") -> data_day9_LP
cor.test(data_day9_LP$root_PO4, data_day9_LP$Total_length,
na.omit=TRUE)

##
##  Pearson's product-moment correlation
##
```

```
## data:  data_day9_LP$root_PO4 and data_day9_LP$Total_length
## t = -2.177, df = 348, p-value = 0.03015
## alternative hypothesis: true correlation is not equal to 0
## 95 percent confidence interval:
##  -0.21809214 -0.01121887
## sample estimates:
##        cor
## -0.1159124

data %>% filter (data$day=="9" & phosphate == "750P") -> data_day9_HP
cor.test(data_day9_HP$root_PO4, data_day9_HP$Total_length,
na.omit=TRUE)

##
##  Pearson's product-moment correlation
##
## data:  data_day9_HP$root_PO4 and data_day9_HP$Total_length
## t = -0.52607, df = 362, p-value = 0.5992
## alternative hypothesis: true correlation is not equal to 0
## 95 percent confidence interval:
##  -0.13006106  0.07536685
## sample estimates:
##         cor
## -0.02763892
```

Figure 3 a - overlap between SNPs

```
##mapping overlapping top SNPs < 10-5
#install.packages("UpSetR")
library("UpSetR")
data_overlap <- read.csv
("/Volumes/busch/lab_new/Marco/20180301_draft_manuscript/overlap/201901
18_overlapping_top_SNPs.csv")  ###Supplementary table 8
data1 <- data_overlap [,c("Target_anions", "Target_brat")]
listInput <- list(Lotus_phosphate = data1$Target_anions, Lotus_brat =
data1$Target_brat)
upset(fromList(listInput),text.scale=c(1.3, 1.3, 1, 1.1, 1.5, 3))
```

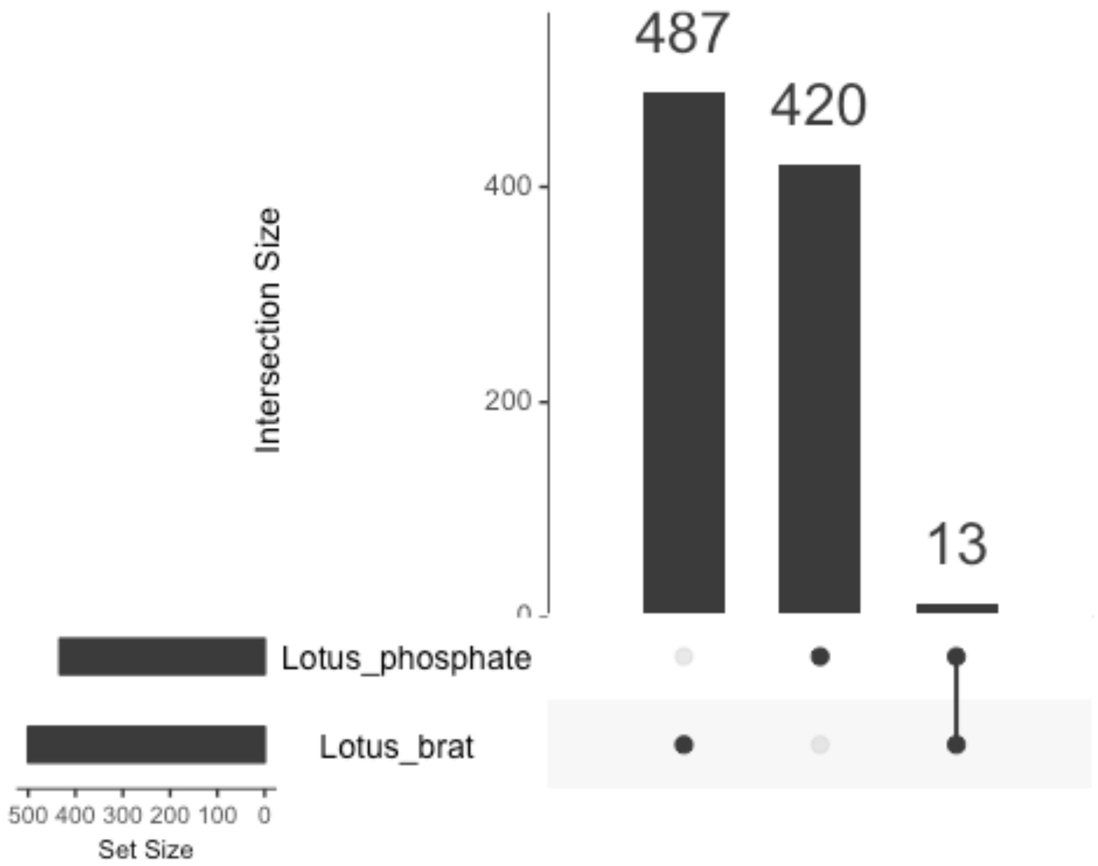

Figure 4b

```
###plots for cytB5 mutants
data <-
read.csv("/Volumes/busch/lab_new/Marco/20180301_draft_manuscript/figure
/set10_set11_merge/20181130_set10_set11_merge.csv", header=T,
na.string=c("na", "NA", "null"))
summary(data)

##     sample..          set             genotype             organ
##  Min.   :  1.0   Min.   :10.00   gifu-wt  :108   root       :204
##  1st Qu.: 51.0   1st Qu.:10.00   43.15    : 54   shoot      :204
##  Median :102.5   Median :10.00   53.29    : 54   total_plant:204
##  Mean   :102.7   Mean   :10.47   54.25_wt : 54
##  3rd Qu.:153.0   3rd Qu.:11.00   54.4_mut : 54
##  Max.   :216.0   Max.   :11.00   55.12_mut: 54
##                                  (Other)  :234
##  treatment    weight_mg      adj_abs_over_weight   rel_P_conc
##  100P:198   Min.   : 2.00   Min.   :0.1687      Min.   : 0.3884
##  20P :216   1st Qu.: 6.70   1st Qu.:1.0992      1st Qu.: 1.7391
##  750P:198   Median : 9.50   Median :1.6890      Median : 2.7464
##             Mean   :10.21   Mean   :1.7403      Mean   : 3.2312
##             3rd Qu.:13.62   3rd Qu.:2.3500      3rd Qu.: 4.5379
##             Max.   :23.60   Max.   :4.3288      Max.   :10.7216
```

```
##
##   rel_P_conc2             X                X.1               X.2        
##  Min.   : 0.3884   Min.   :0.0002   Min.   :0.3132   Min.   :0.1938  
##  1st Qu.: 2.4552   1st Qu.:0.0081   1st Qu.:0.3308   1st Qu.:0.3938  
##  Median : 3.7989   Median :0.0692   Median :0.3717   Median :0.4037  
##  Mean   : 3.8705   Mean   :0.1148   Mean   :0.4037   Mean   :0.4854  
##  3rd Qu.: 5.1138   3rd Qu.:0.1932   3rd Qu.:0.4869   3rd Qu.:0.6315  
##  Max.   :10.7216   Max.   :0.4543   Max.   :0.5234   Max.   :0.7497  
##                    NA's   :599      NA's   :606      NA's   :605     
##       X.3       
##  Min.   :0.499  
##  1st Qu.:0.499  
##  Median :0.499  
##  Mean   :0.499  
##  3rd Qu.:0.499  
##  Max.   :0.499  
##  NA's   :611
```

```
data$treatment = factor(data$treatment, levels=c('20P','100P', '750P'))
data$genotype = factor(data$genotype,
levels=c('53.29','54.4_mut','55.12_mut','gifu-wt'))
data <- mutate (data, genotype= fct_recode(genotype,  "cyt3" =
"53.29"))
data <- mutate (data, genotype= fct_recode(genotype,  "cyt2" =
"54.4_mut"))
data <- mutate (data, genotype= fct_recode(genotype,  "cyt1" =
"55.12_mut"))
data$genotype = factor(data$genotype,
levels=c('cyt3','cyt2','cyt1','gifu-wt'))
data %>%
      filter(genotype %in% c('cyt3','cyt2','cyt1','gifu-wt')) %>%
      filter(set==10) %>%
  split(.$treatment) %>%
  map(
        ~ggplot(data=.,aes(x=genotype, y=rel_P_conc2, fill=genotype)) +
              scale_fill_manual(values=c( "#79c4c1",  "#79c4c1",
"#79c4c1","#666666","#666666")) +
              geom_dotplot(binaxis ="y", stackdir = "center", dotsize =
2.5) +
                  stat_summary(fun.y = median, fun.ymin = median,
fun.ymax = median, geom = "crossbar", width = 0.5) +
        theme_classic()  +
              theme(text = element_text(size=13),
legend.position="none") +
              ylab("relative plant phosphate concentration") +
        facet_wrap(~organ, scales="free") +
        coord_flip() +
        ggtitle(paste0("Media phosphate concentration ",
unique(.$treatment)))
  )
```

```
## $`20P`

## `stat_bindot()` using `bins = 30`. Pick better value with
`binwidth`.
```

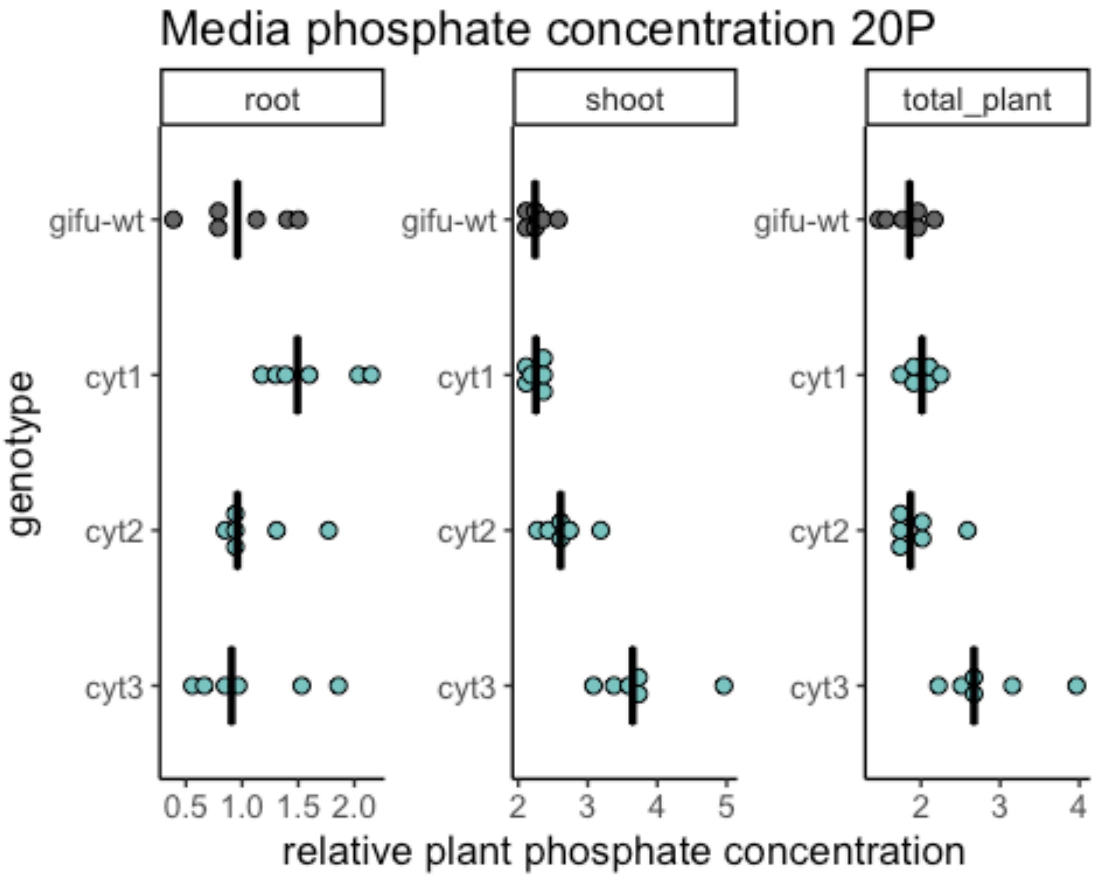


```
##
## $`100P`

## `stat_bindot()` using `bins = 30`. Pick better value with
`binwidth`.
```

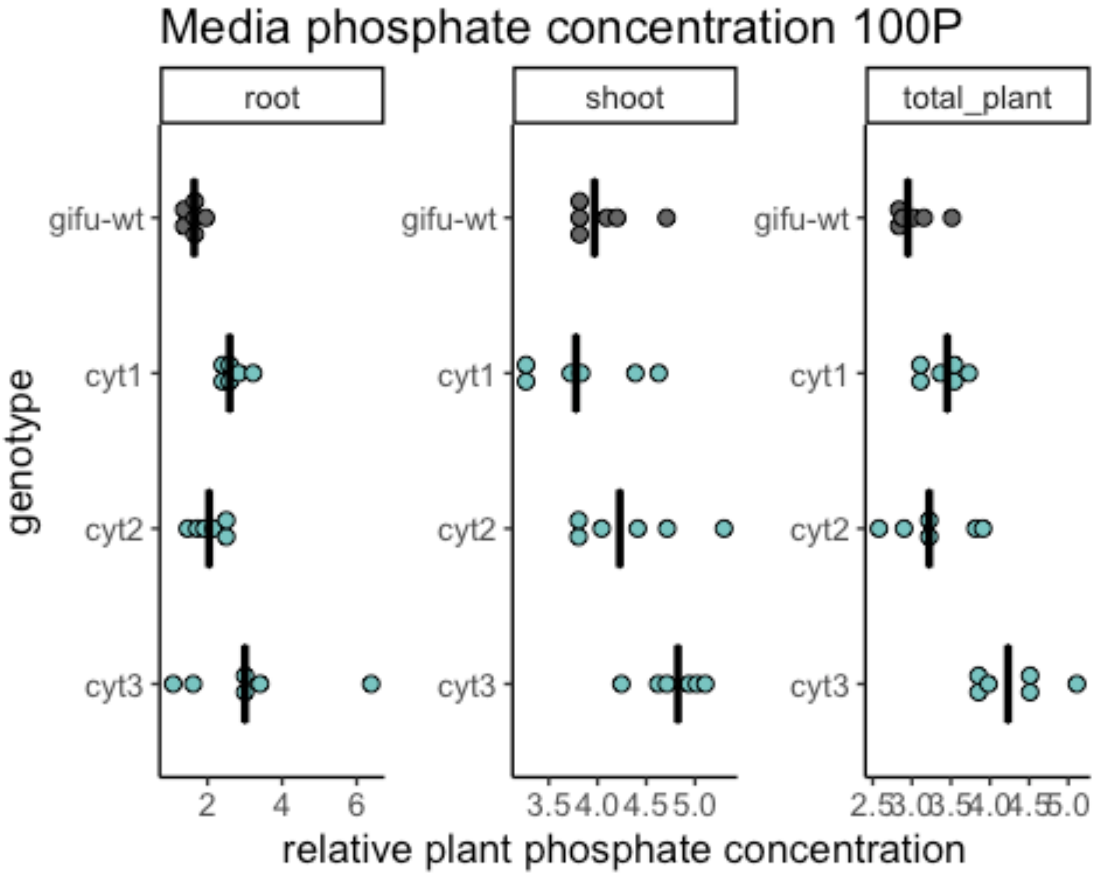


```
##
## $`750P`

## `stat_bindot()` using `bins = 30`. Pick better value with
`binwidth`.
```

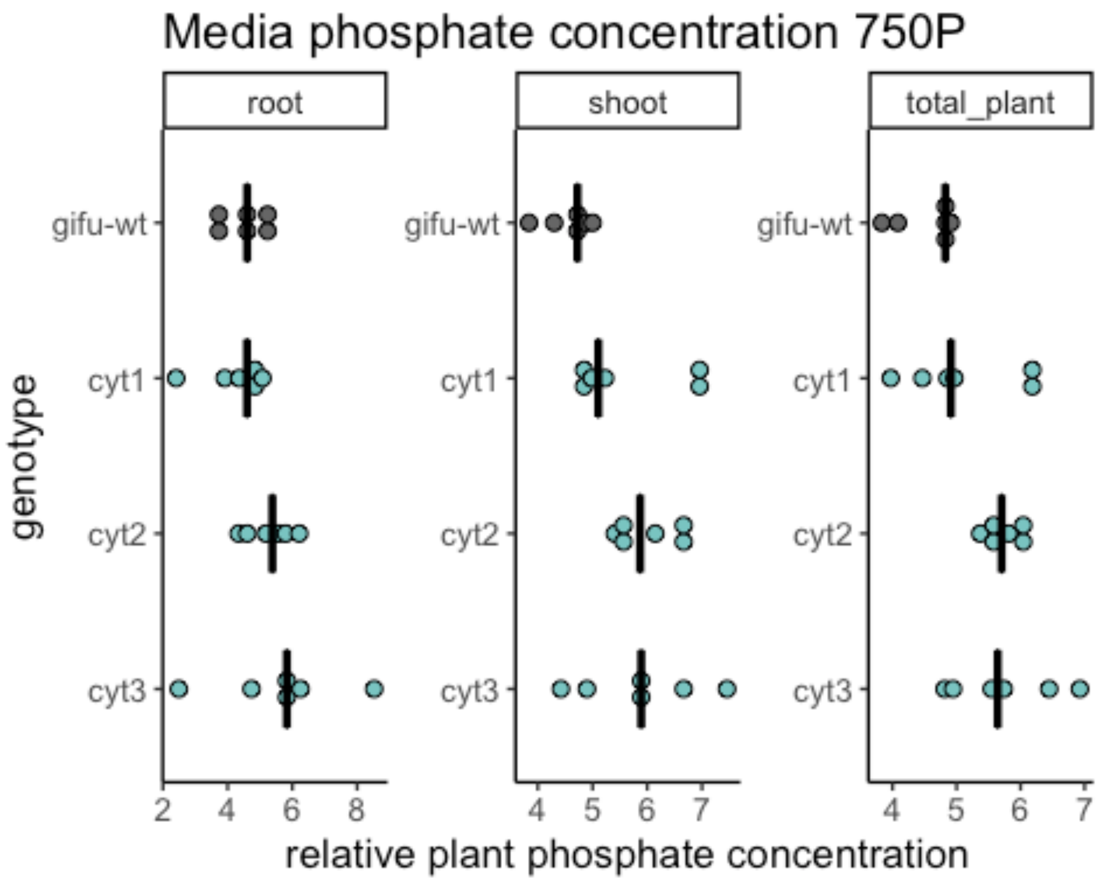

Figure 4a

```
library(emmeans)
data <-
read.csv("/Volumes/busch/lab_new/Marco/20180301_draft_manuscript/figure
/set10_set11_merge/20181130_set10_set11_merge.csv", header=T,
na.string=c("na", "NA", "null"))
summary(data)

##     sample..          set               genotype            organ
##  Min.   :  1.0   Min.   :10.00   gifu-wt  :108   root       :204
##  1st Qu.: 51.0   1st Qu.:10.00   43.15    : 54   shoot      :204
##  Median :102.5   Median :10.00   53.29    : 54   total_plant:204
##  Mean   :102.7   Mean   :10.47   54.25_wt : 54
##  3rd Qu.:153.0   3rd Qu.:11.00   54.4_mut : 54
##  Max.   :216.0   Max.   :11.00   55.12_mut: 54
##                                  (Other)  :234
##  treatment    weight_mg     adj_abs_over_weight   rel_P_conc
##  100P:198   Min.   : 2.00   Min.   :0.1687      Min.   : 0.3884
##  20P :216   1st Qu.: 6.70   1st Qu.:1.0992      1st Qu.: 1.7391
##  750P:198   Median : 9.50   Median :1.6890      Median : 2.7464
##             Mean   :10.21   Mean   :1.7403      Mean   : 3.2312
##             3rd Qu.:13.62   3rd Qu.:2.3500      3rd Qu.: 4.5379
##             Max.   :23.60   Max.   :4.3288      Max.   :10.7216
```

```
##
##    rel_P_conc2            X                X.1              X.2
##   Min.    : 0.3884   Min.    :0.0002   Min.    :0.3132   Min.    :0.1938
##   1st Qu.: 2.4552   1st Qu.:0.0081   1st Qu.:0.3308   1st Qu.:0.3938
##   Median : 3.7989   Median :0.0692   Median :0.3717   Median :0.4037
##   Mean   : 3.8705   Mean   :0.1148   Mean   :0.4037   Mean   :0.4854
##   3rd Qu.: 5.1138   3rd Qu.:0.1932   3rd Qu.:0.4869   3rd Qu.:0.6315
##   Max.   :10.7216   Max.   :0.4543   Max.   :0.5234   Max.   :0.7497
##                     NA's   :599      NA's   :606      NA's   :605
##        X.3
##   Min.   :0.499
##   1st Qu.:0.499
##   Median :0.499
##   Mean   :0.499
##   3rd Qu.:0.499
##   Max.   :0.499
##   NA's   :611

data$treatment = factor(data$treatment, levels=c('20P','100P', '750P'))

colnames(data)[7] <- "P_conc"

###Plots
data <- data %>%
      filter(!genotype %in% c("P9-18", "P8-8","P5-20","P3-9", "62.4"))
###plots for LRR mutants
###emmeans for set 11 merging gifu with 61.3_WT
data %>% filter (organ=="total_plant" & genotype != "54.25_wt") %>%
filter (set==11) %>%
      #filter (genotype != "62.4") %>%
      mutate (., genotype= fct_recode(genotype,  "gifu-wt" =
"61.3_wt")) %>%
  split(.$treatment) %>%
  map(
#aov <- aov(Total_length_day003 ~ genotype*treatment, data)
~lm(rel_P_conc ~ genotype , data = .x) %>%
   emmeans(., ~ genotype, options = list(adjust = "tukey", alpha =
0.05, level = 0.95)) %>%
    cld(Letters = letters, alpha = 0.05)
)

## $`20P`
##   genotype   emmean         SE df  lower.CL upper.CL .group
##   gifu-wt  1.084128 0.07376623 26 0.8867755 1.281481  a
##   61.7_mut 1.276006 0.10432121 26 0.9969073 1.555105  ab
##   59.27    1.370307 0.10432121 26 1.0912084 1.649406  ab
##   43.15    1.546780 0.10432121 26 1.2676818 1.825879   b
##
## Confidence level used: 0.95
## Conf-level adjustment: sidak method for 4 estimates
```

```
## P value adjustment: tukey method for comparing a family of 4
estimates
## significance level used: alpha = 0.05
##
## $`100P`
##  genotype   emmean         SE df lower.CL upper.CL .group
##  gifu-wt  1.985194 0.08749244 26 1.751119 2.219269  a
##  59.27    2.162704 0.12373299 26 1.831671 2.493736  ab
##  43.15    2.273573 0.12373299 26 1.942540 2.604605  ab
##  61.7_mut 2.630563 0.12373299 26 2.299530 2.961595   b
##
## Confidence level used: 0.95
## Conf-level adjustment: sidak method for 4 estimates
## P value adjustment: tukey method for comparing a family of 4
estimates
## significance level used: alpha = 0.05
##
## $`750P`
##  genotype   emmean        SE df lower.CL upper.CL .group
##  gifu-wt  2.597810 0.1134713 26 2.294231 2.901388  a
##  61.7_mut 3.141698 0.1604726 26 2.712373 3.571023   b
##  59.27    3.225898 0.1604726 26 2.796573 3.655223   b
##  43.15    3.592227 0.1604726 26 3.162902 4.021552   b
##
## Confidence level used: 0.95
## Conf-level adjustment: sidak method for 4 estimates
## P value adjustment: tukey method for comparing a family of 4
estimates
## significance level used: alpha = 0.05

data <- mutate (data, genotype= fct_recode(genotype,  "gifu-wt" =
"61.3_wt"))
data <- mutate (data, genotype= fct_recode(genotype,  "lrr3" =
"59.27"))
data <- mutate (data, genotype= fct_recode(genotype,  "lrr2" =
"43.15"))
data <- mutate (data, genotype= fct_recode(genotype,  "lrr1" =
"61.7_mut"))
data$genotype = factor(data$genotype,
levels=c('lrr3','lrr2','lrr1','gifu-wt'))

  data %>%   filter(genotype %in% c('lrr3','lrr2','lrr1','gifu-wt'))
%>%
      filter(set==11) %>%

  split(.$treatment) %>%
  map(
       ~ggplot(data=.,aes(x=genotype, y=rel_P_conc2, fill=genotype)) +
             scale_fill_manual(values=c( "#79c4c1",  "#79c4c1",
"#79c4c1","#666666","#666666")) +
```

```
              geom_dotplot(binaxis ="y", stackdir = "center", dotsize =
2.5) +
                  stat_summary(fun.y = median, fun.ymin = median,
fun.ymax = median, geom = "crossbar", width = 0.5) +
        theme_classic()  +
        theme(text = element_text(size=13), legend.position="none") +
        ylab("relative plant phosphate concentration") +
        facet_wrap(~organ, scales="free") +
            coord_flip() +
        ggtitle(paste0("Media phosphate concentration ",
unique(.$treatment)))
  )

## $`20P`

## `stat_bindot()` using `bins = 30`. Pick better value with
`binwidth`.
```

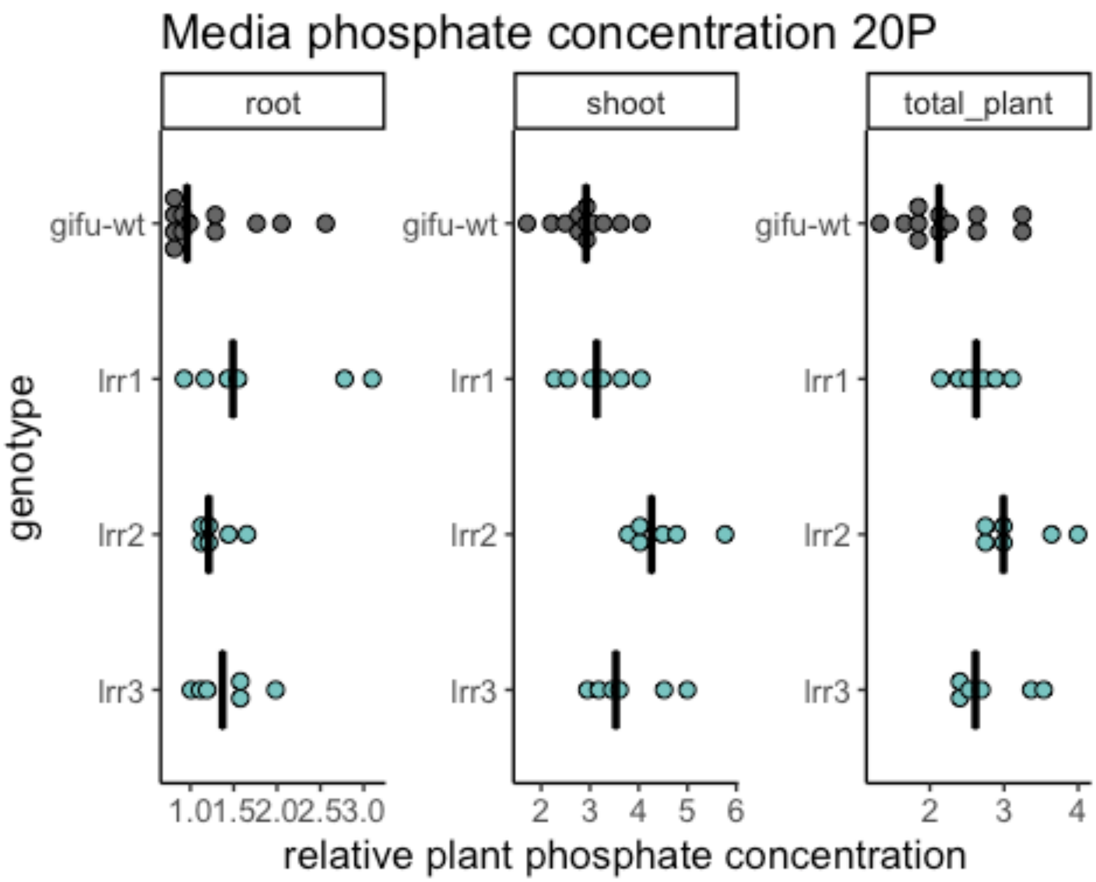


```
##
## $`100P`

## `stat_bindot()` using `bins = 30`. Pick better value with
`binwidth`.
```

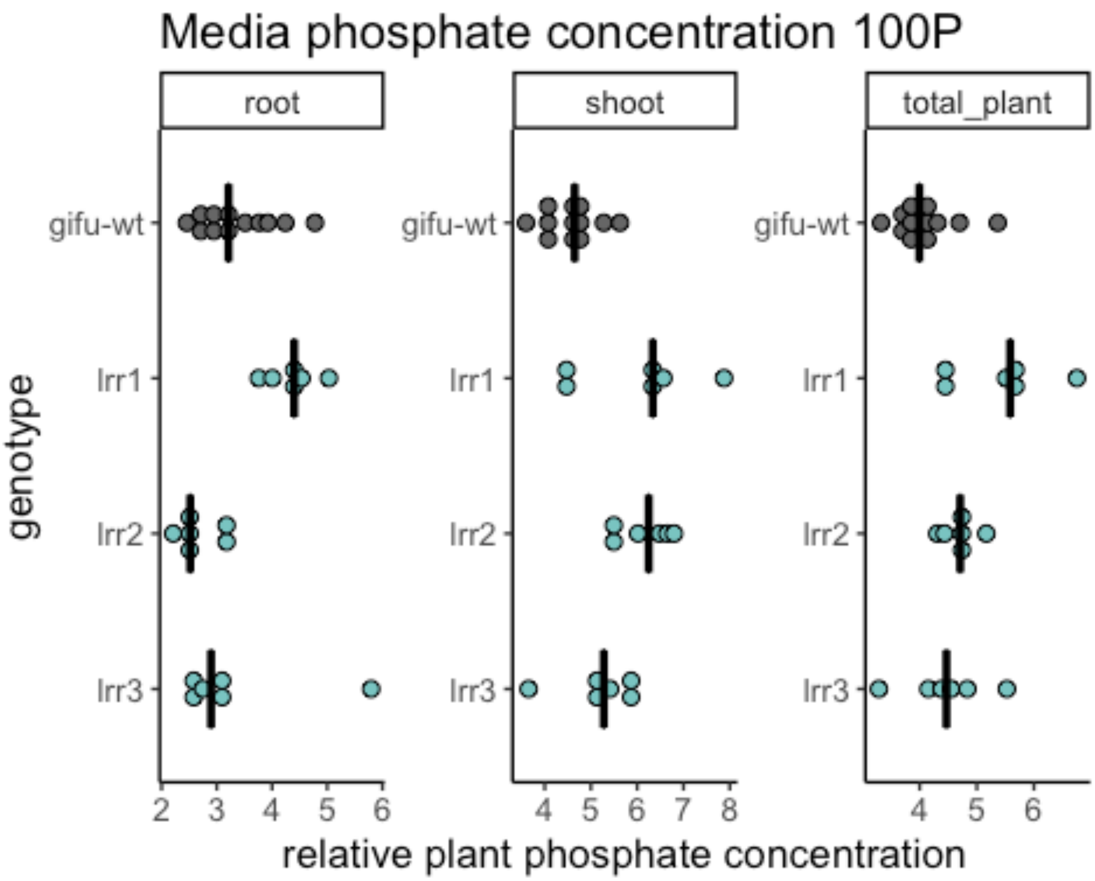


```
##
## $`750P`

## `stat_bindot()` using `bins = 30`. Pick better value with
`binwidth`.
```

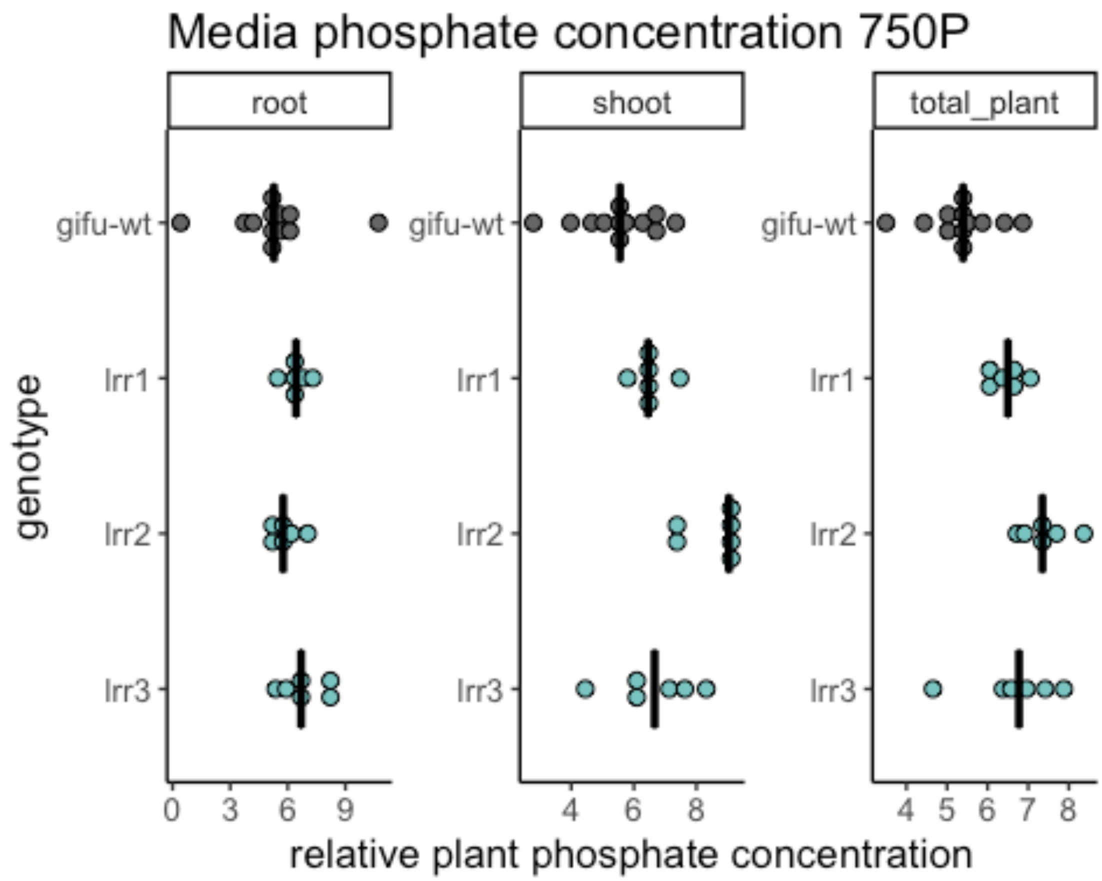

Figure 5 - correlation plot

```
data <-
read.csv("/Volumes/busch/lab_new/Marco/20180301_draft_manuscript/figure
/set10_set11_merge/20181130_set10_set11_merge.csv", header=T,
na.string=c("na", "NA", "null"))
summary(data)

##     sample..          set              genotype           organ
##  Min.   :  1.0   Min.   :10.00   gifu-wt  :108   root       :204
##  1st Qu.: 51.0   1st Qu.:10.00   43.15    : 54   shoot      :204
##  Median :102.5   Median :10.00   53.29    : 54   total_plant:204
##  Mean   :102.7   Mean   :10.47   54.25_wt : 54
##  3rd Qu.:153.0   3rd Qu.:11.00   54.4_mut : 54
##  Max.   :216.0   Max.   :11.00   55.12_mut: 54
##                                  (Other)  :234
##  treatment    weight_mg     adj_abs_over_weight   rel_P_conc
##  100P:198   Min.   : 2.00   Min.   :0.1687      Min.   : 0.3884
##  20P :216   1st Qu.: 6.70   1st Qu.:1.0992      1st Qu.: 1.7391
##  750P:198   Median : 9.50   Median :1.6890      Median : 2.7464
##             Mean   :10.21   Mean   :1.7403      Mean   : 3.2312
##             3rd Qu.:13.62   3rd Qu.:2.3500      3rd Qu.: 4.5379
##             Max.   :23.60   Max.   :4.3288      Max.   :10.7216
##
```

```
##   rel_P_conc2            X                 X.1               X.2        
##  Min.   : 0.3884   Min.   :0.0002   Min.   :0.3132   Min.   :0.1938  
##  1st Qu.: 2.4552   1st Qu.:0.0081   1st Qu.:0.3308   1st Qu.:0.3938  
##  Median : 3.7989   Median :0.0692   Median :0.3717   Median :0.4037  
##  Mean   : 3.8705   Mean   :0.1148   Mean   :0.4037   Mean   :0.4854  
##  3rd Qu.: 5.1138   3rd Qu.:0.1932   3rd Qu.:0.4869   3rd Qu.:0.6315  
##  Max.   :10.7216   Max.   :0.4543   Max.   :0.5234   Max.   :0.7497  
##                    NA's   :599      NA's   :606      NA's   :605     
##       X.3       
##  Min.   :0.499  
##  1st Qu.:0.499  
##  Median :0.499  
##  Mean   :0.499  
##  3rd Qu.:0.499  
##  Max.   :0.499  
##  NA's   :611

data$treatment = factor(data$treatment, levels=c('20P','100P', '750P'))

colnames(data)[7] <- "P_conc"
#correlation plot
data %>%
  split(.$treatment) %>%
  map(
        ~ggplot(data=.,aes(x=weight_mg, y=rel_P_conc2)) +
              geom_smooth(method=lm, level=0.90, fullrange=TRUE) +
              geom_point(size =2) +
              theme_classic()  +
              facet_wrap(~organ, scales="free") +
              ggtitle(paste0("Media phosphate concentration ",
unique(.$treatment)))

        )

## $`20P`
```

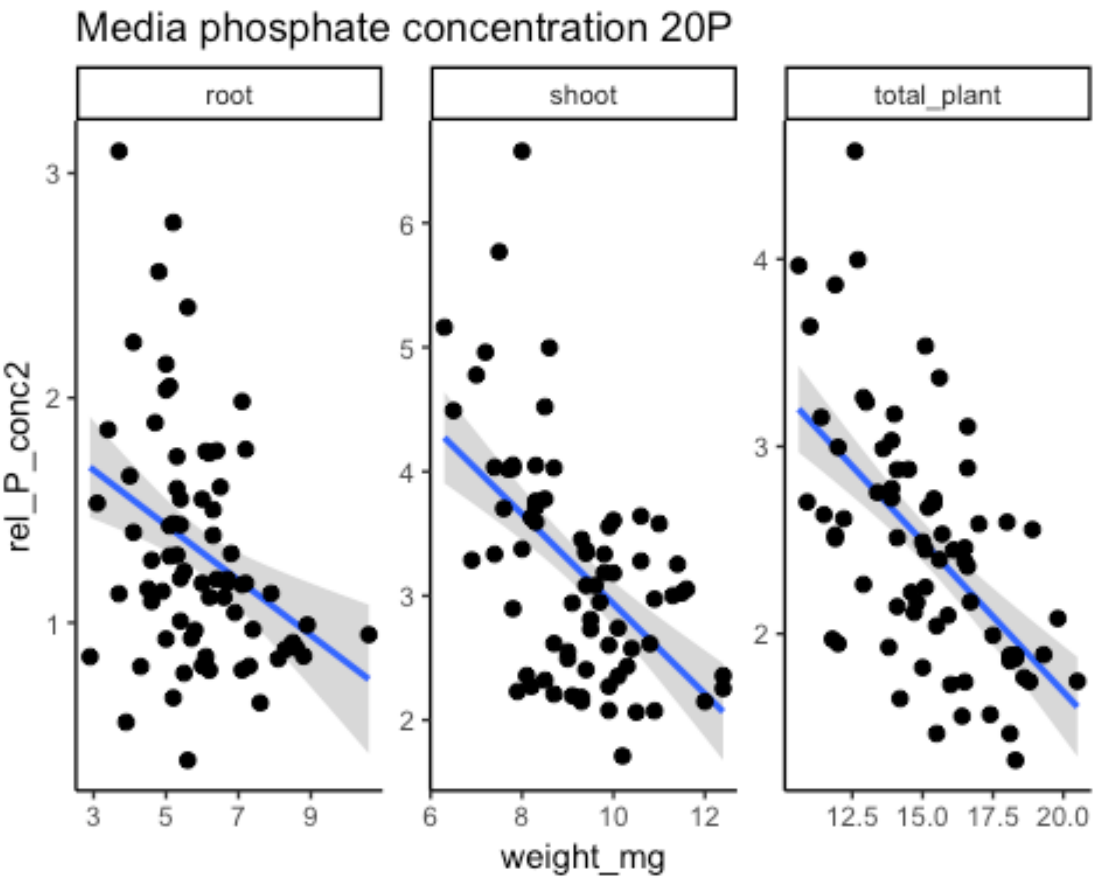
Media phosphate concentration 20P
root
shoot
total_plant
rel_P_conc2
weight_mg
3
2
1
6
5
4
3
2
4
3
2
3
5
7
9
6
8
10
12
12.5
15.0
17.5
20.0

```
##
## $`100P`
```

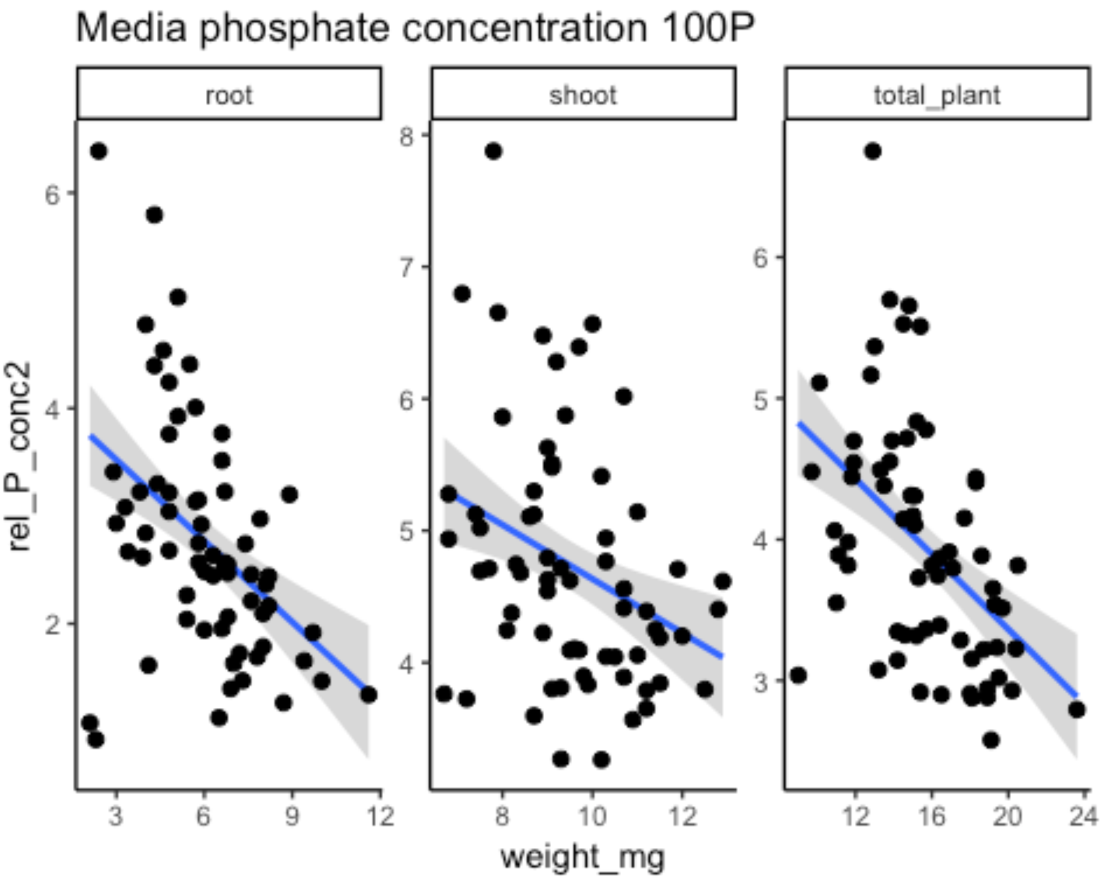
Media phosphate concentration 100P
root
shoot
total_plant
rel_P_conc2
weight_mg
2
4
6
3
6
9
12
4
5
6
7
8
8
10
12
3
4
5
6
12
16
20
24

```
##
## $`750P`
```

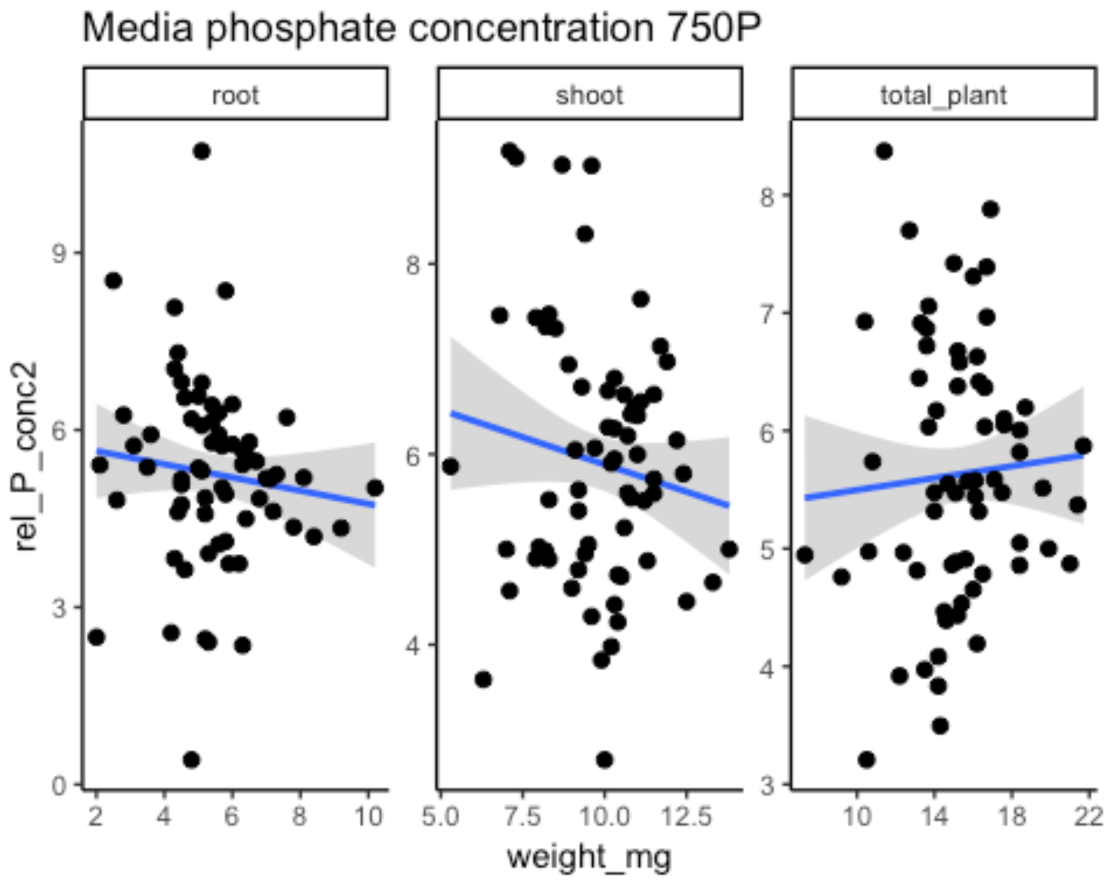

Supplementary Figure 7b

```
###plots for cytB5 mutants root growth scored manually with Fiji
library(tidyverse)
data <-
read.csv("/Volumes/busch/lab_new/Marco/20180301_draft_manuscript/figure
/mutants_root_growth/CYTB5/20181220_scoring_root_growth_mutants.csv",
header=T, na.string=c("na", "NA", "null"))
summary(data)

##                              Plate.ID      Plate          Genotype
Treatment
##                                  :74   Min.   : 4.00   53.29   :15
100P:25
##   P_set10_day0_20181029_004.tif: 1   1st Qu.: 7.00   54.4    :17   20P
:30
##                                        Median :24.00   55.12   :18
750P:20
##                                        Mean   :22.95   gifu-wt:25
##                                        3rd Qu.:27.00
##                                        Max.   :48.00
##
##    Replicate             X0               X2               X4
##   Min.    : 1.000   Min.    :238.8   Min.    :355.2   Min.    : 396.1
```

```
##  1st Qu.: 2.000   1st Qu.:391.7   1st Qu.:575.1   1st Qu.: 704.1
##  Median : 4.000   Median :448.8   Median :676.9   Median : 868.0
##  Mean   : 3.893   Mean   :457.2   Mean   :668.8   Mean   : 837.9
##  3rd Qu.: 5.000   3rd Qu.:519.2   3rd Qu.:757.2   3rd Qu.: 971.9
##  Max.   :10.000   Max.   :699.8   Max.   :898.0   Max.   :1187.5
##
##        X7               X10              LRl.1            LRl.2
##  Min.   : 556.8   Min.   : 615.3   Min.   : 44.6   Min.   : 44.72
##  1st Qu.: 889.3   1st Qu.:1028.5   1st Qu.:102.4   1st Qu.: 73.76
##  Median :1110.8   Median :1297.6   Median :141.6   Median :110.31
##  Mean   :1082.3   Mean   :1264.6   Mean   :154.4   Mean   :118.06
##  3rd Qu.:1271.7   3rd Qu.:1496.8   3rd Qu.:192.3   3rd Qu.:130.23
##  Max.   :1590.5   Max.   :1806.7   Max.   :433.3   Max.   :259.31
##                                    NA's   :36      NA's   :62
##      LRl.3            LR.             LRL              ARG
##  Min.   :34.99   Min.   :0.00   Min.   : 51.90   Min.   : 55.36
##  1st Qu.:36.44   1st Qu.:0.00   1st Qu.: 95.03   1st Qu.:120.91
##  Median :37.90   Median :1.00   Median :141.60   Median :161.32
##  Mean   :37.90   Mean   :0.72   Mean   :150.20   Mean   :161.48
##  3rd Qu.:39.35   3rd Qu.:1.00   3rd Qu.:190.39   3rd Qu.:197.88
##  Max.   :40.80   Max.   :3.00   Max.   :433.33   Max.   :259.19
##  NA's   :73                     NA's   :36
##      avg_P4             X                X.1               X.2
##  Min.   : 957.4   Min.   :0.6151   Min.   :0.7484   Min.   :0.00037
##  1st Qu.:1208.7   1st Qu.:0.7881   1st Qu.:0.8716   1st Qu.:0.00969
##  Median :1344.9   Median :0.9143   Median :0.9167   Median :0.07348
##  Mean   :1290.4   Mean   :0.9239   Mean   :0.9336   Mean   :0.08463
##  3rd Qu.:1426.6   3rd Qu.:1.0437   3rd Qu.:0.9724   3rd Qu.:0.14283
##  Max.   :1514.6   Max.   :1.2617   Max.   :1.1636   Max.   :0.20605
##  NA's   :71       NA's   :30       NA's   :67       NA's   :69
```

```r
data$Treatment = factor(data$Treatment, levels=c('20P','100P', '750P'))
data$Genotype = factor(data$Genotype,
levels=c('53.29','54.4','55.12','gifu-wt'))

data <- data %>%
mutate(`Genotype` = str_replace(`Genotype`, "53.29", "cyt2")) %>%
mutate(`Genotype` = str_replace(`Genotype`, "54.4", "cyt3")) %>%
mutate(`Genotype` = str_replace(`Genotype`, "55.12", "cyt1"))

data$Genotype <- as.factor(data$Genotype)
data <- gather(data, colnames(data[6:10]), key = 'day', value= 'PR',
convert = TRUE)

data$day = factor(data$day, levels=c('X0', 'X2', 'X4', 'X7', 'X10'))

data <- mutate (data, day2 = extract_numeric(day))
```

```
## extract_numeric() is deprecated: please use readr::parse_number()
instead
```

```
data %>%

  split(.$Treatment) %>%
  map(
        ~ggplot(data=.,aes(x=day2, y=PR, colour=Genotype)) +
              scale_colour_manual(values=c( "#1b9e77",  "#d95f02",
"#7570b3","#636363")) +
              stat_smooth(method ="lm", se=TRUE, alpha=0.2, size = 2,
linetype = 6) +
              theme_classic()  +
              theme(text = element_text(size=15)) +
        ggtitle(paste0("Media phosphate concentration ",
unique(.$Treatment)))
  )

## $`20P`
```

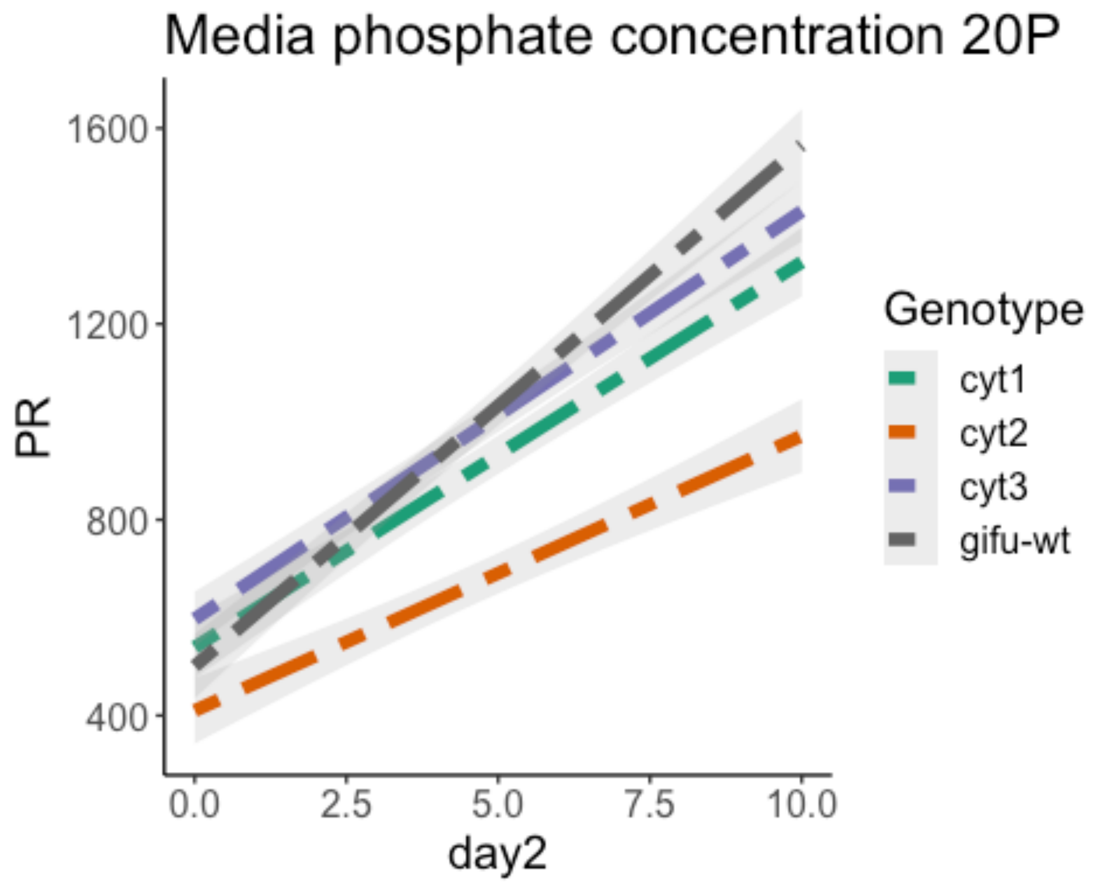


```
##
## $`100P`
```

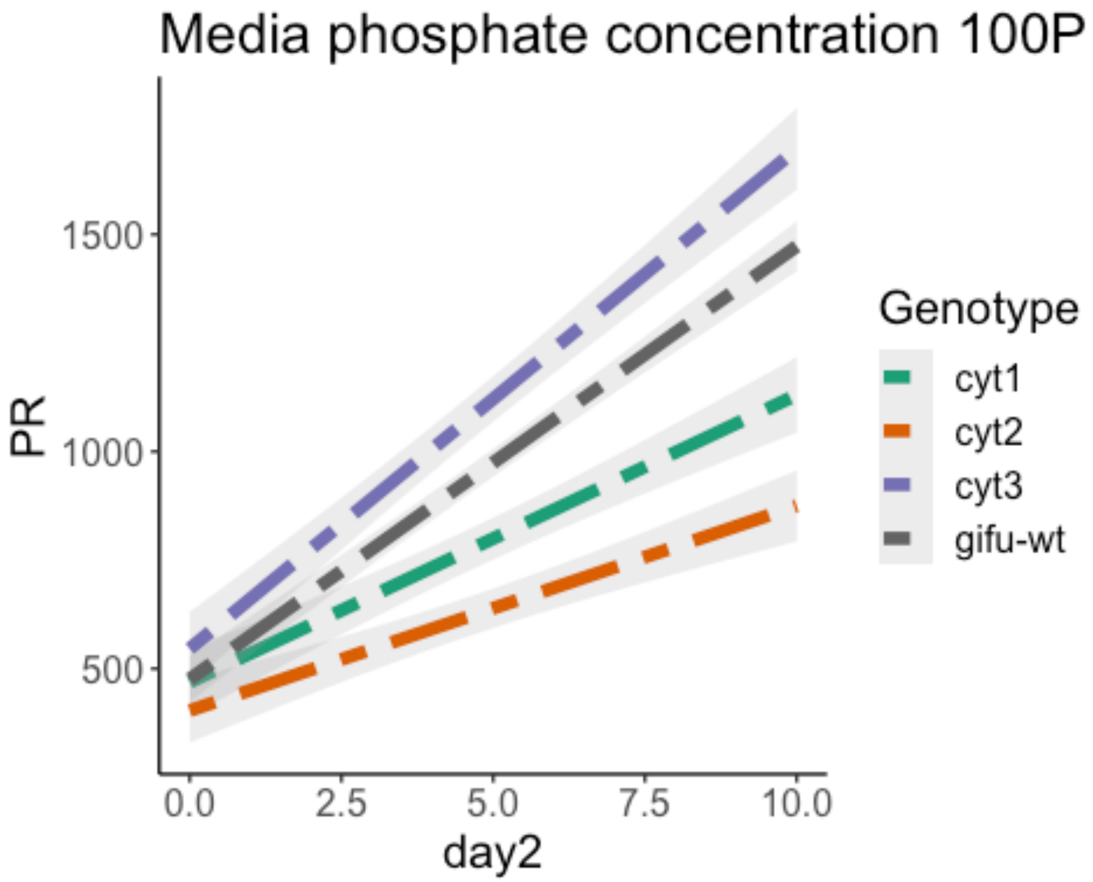

Media phosphate concentration 100P
PR
1500
1000
500
0.0
2.5
5.0
7.5
10.0
day2
Genotype
cyt1
cyt2
cyt3
gifu-wt


```
##
## $`750P`
```

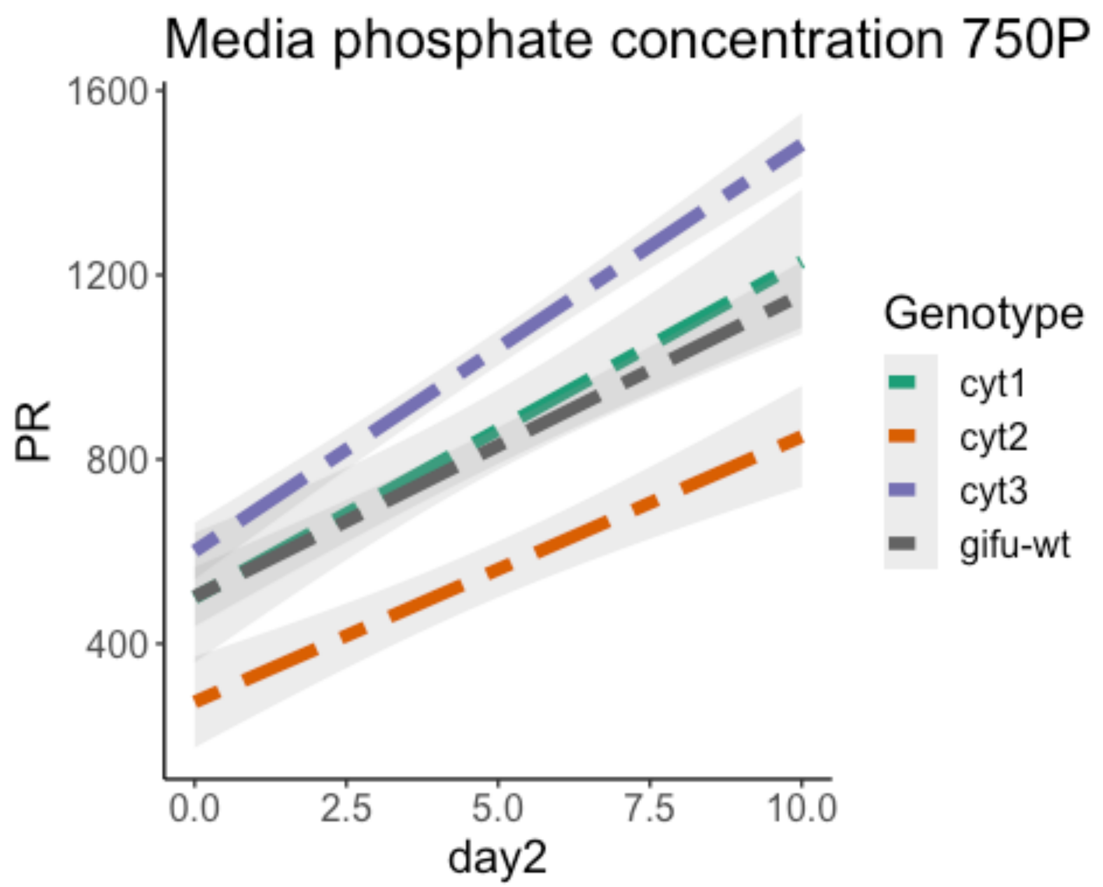


```
ggplot(data,aes(x=day2, y=PR, colour=Genotype)) +
              scale_colour_manual(values=c( "#1b9e77",  "#d95f02",
"#7570b3","#636363")) +
              stat_smooth(method ="lm", se=TRUE, alpha=0.2, size = 2,
linetype = 6) +
              theme_classic()  +
              theme(text = element_text(size=15)) +
              labs(x = "Time (d)", y = "Primary root length (px)",
title = "CYT-B5 root growth over phosphate concentration") +
              facet_grid(~Treatment)
```

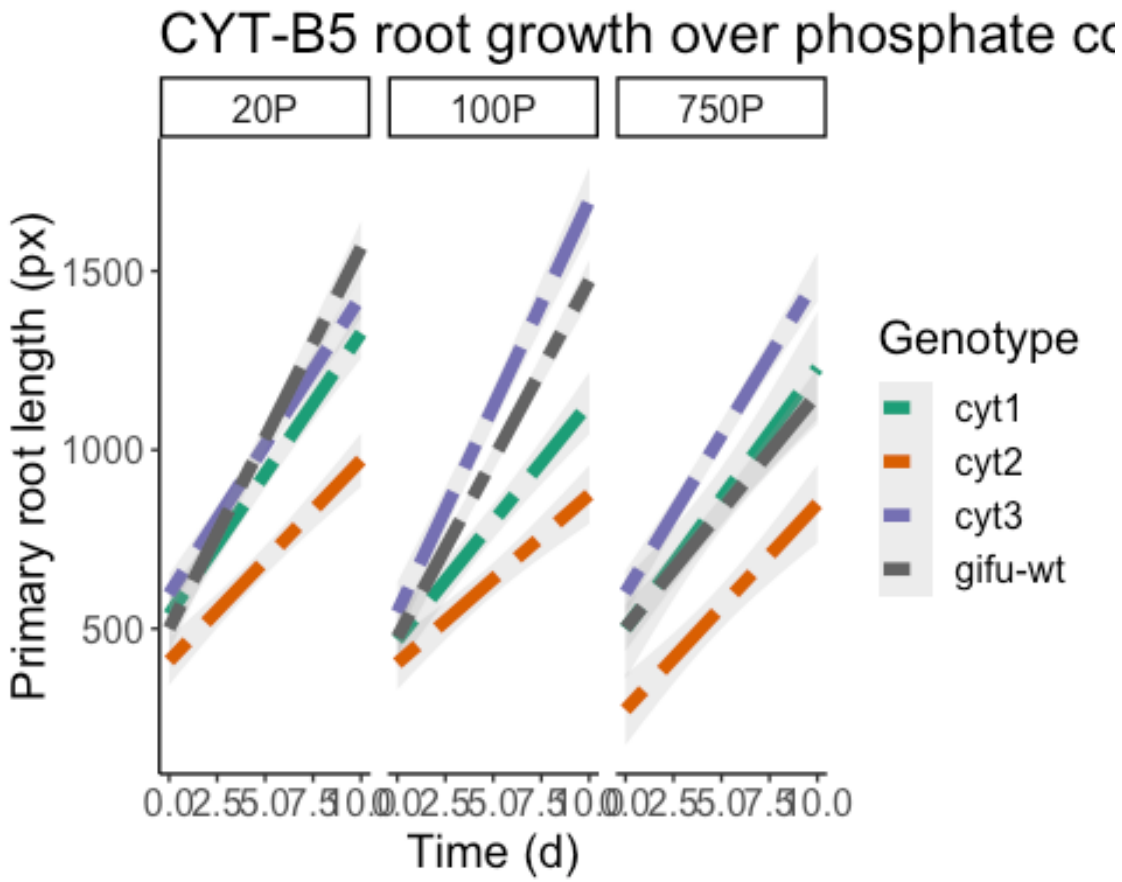

Supplementary Figure 7a

```
library(gridExtra)

##
## Attaching package: 'gridExtra'

## The following object is masked from 'package:dplyr':
##
##     combine

library(tidyverse)
data <-
read.csv("/Volumes/busch/lab_new/Marco/20180301_draft_manuscript/figure
/mutants_root_growth/LRR-
RK/20181220_scoring_set11_LRR_root_growth_mutants.csv", header=T,
na.string=c("na", "NA", "null"))
summary(data)

##          Plate      Genotype    Treat      Replicate          P0
##  4          :11    43.6 :23   100P:29    Min.   : 1.000   Min.
:318.4
##  6          :11    59.27:30   20P :33    1st Qu.: 2.750   1st
Qu.:490.4
```

```
##  3           : 9   61.7 :19   750P:34   Median : 4.500   Median
:539.6
##  46-43-37-29: 8   gifu :24             Mean   : 4.656   Mean
:535.3
##  47-44-38-30: 8                        3rd Qu.: 6.250   3rd
Qu.:590.0
##  48-45-39-31: 8                        Max.   :11.000   Max.
:700.0
##  (Other)    :41
##        P5               P7               P9              LRl.1
##  Min.   : 571.3   Min.   : 759.2   Min.   : 762.2   Min.   : 59.17
##  1st Qu.: 907.4   1st Qu.:1052.9   1st Qu.:1232.5   1st Qu.: 94.87
##  Median :1001.5   Median :1177.8   Median :1367.3   Median :169.06
##  Mean   :1010.8   Mean   :1188.5   Mean   :1395.6   Mean   :190.45
##  3rd Qu.:1135.5   3rd Qu.:1304.0   3rd Qu.:1544.9   3rd Qu.:282.40
##  Max.   :1448.3   Max.   :1728.8   Max.   :2004.0   Max.   :418.80
##                                                     NA's   :63
##      LRl.2             LRl.3              LR.              LRL
##  Min.   : 36.12   Min.   : 56.41   Min.   :0.0000   #DIV/0!:63
##  1st Qu.:101.92   1st Qu.: 59.83   1st Qu.:0.0000   106.532: 1
##  Median :121.77   Median : 63.25   Median :0.0000   118.8  : 1
##  Mean   :126.02   Mean   : 90.10   Mean   :0.4479   123.993: 1
##  3rd Qu.:136.99   3rd Qu.:106.95   3rd Qu.:1.0000   130.4  : 1
##  Max.   :246.40   Max.   :150.65   Max.   :3.0000   138.13 : 1
##  NA's   :89       NA's   :93                        (Other):28
##       ARG
##  Min.   : 54.03
##  1st Qu.:138.16
##  Median :171.68
##  Mean   :172.05
##  3rd Qu.:208.78
##  Max.   :297.99
##

data$Treat = factor(data$Treat, levels=c('20P','100P', '750P'))
data$Genotype <- as.factor(data$Genotype)
data$Genotype = factor(data$Genotype,
levels=c('43.6','59.27','61.7','gifu'))
data <- data %>%
mutate(`Genotype` = str_replace(`Genotype`, "61.7", "lrr1")) %>%
mutate(`Genotype` = str_replace(`Genotype`, "43.6", "lrr2")) %>%
mutate(`Genotype` = str_replace(`Genotype`, "59.27", "lrr3")) %>%
mutate(`Genotype` = str_replace(`Genotype`, "gifu", "gifu-wt"))

data$Genotype <- as.factor(data$Genotype)

data <- gather(data, colnames(data[5:8]), key = 'day', value= 'PR',
convert = TRUE)

data <- mutate (data, day2 = extract_numeric(day))
```

```
## extract_numeric() is deprecated: please use readr::parse_number()
instead

data %>%
  split(.$Treat) %>%
  map(
        ~ggplot(data=.,aes(x=day2, y=PR, colour=Genotype)) +
              scale_colour_manual(values=c("#636363", "#1b9e77",
"#d95f02",  "#7570b3")) +
              stat_smooth(method ="lm", se=TRUE, alpha=0.2, size = 2,
linetype = 6) +
        theme_classic()  +
              theme(text = element_text(size=15)) +
        ggtitle(paste0("Media phosphate concentration ",
unique(.$Treat)))
  )

## $`20P`
```

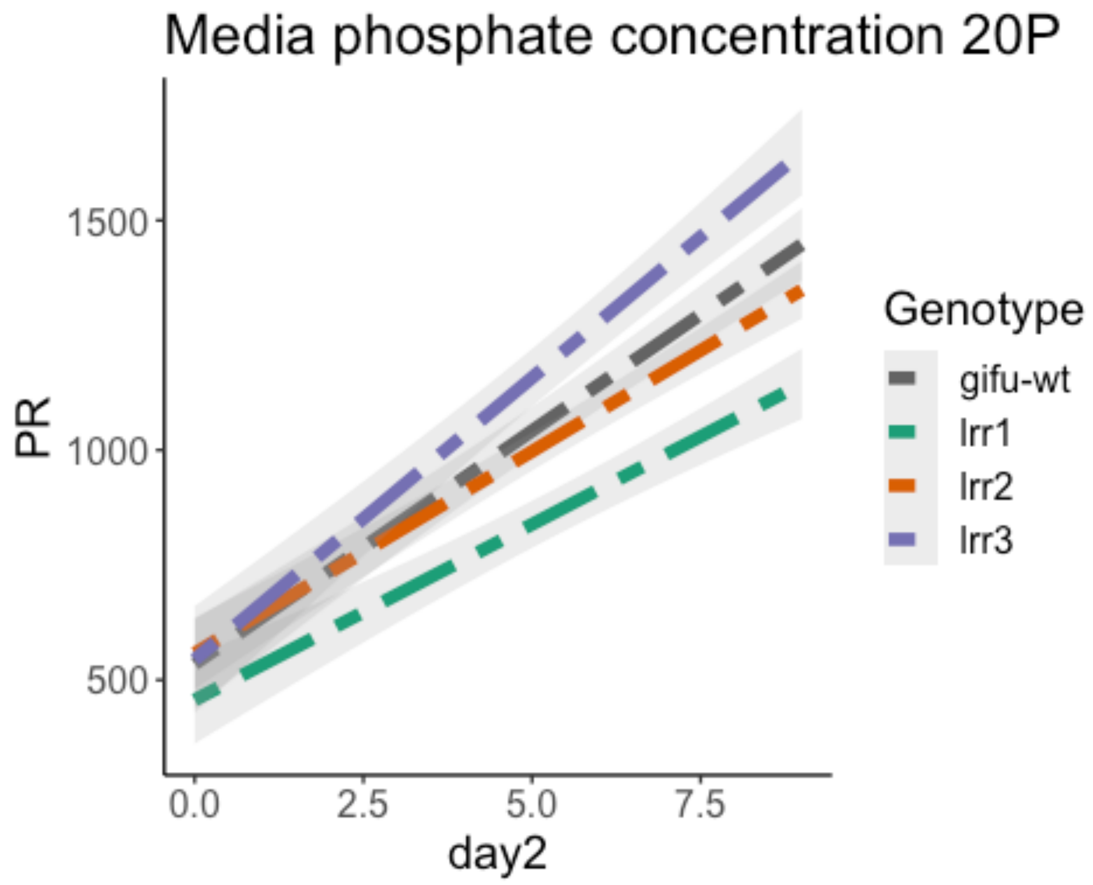


```
##
## $`100P`
```

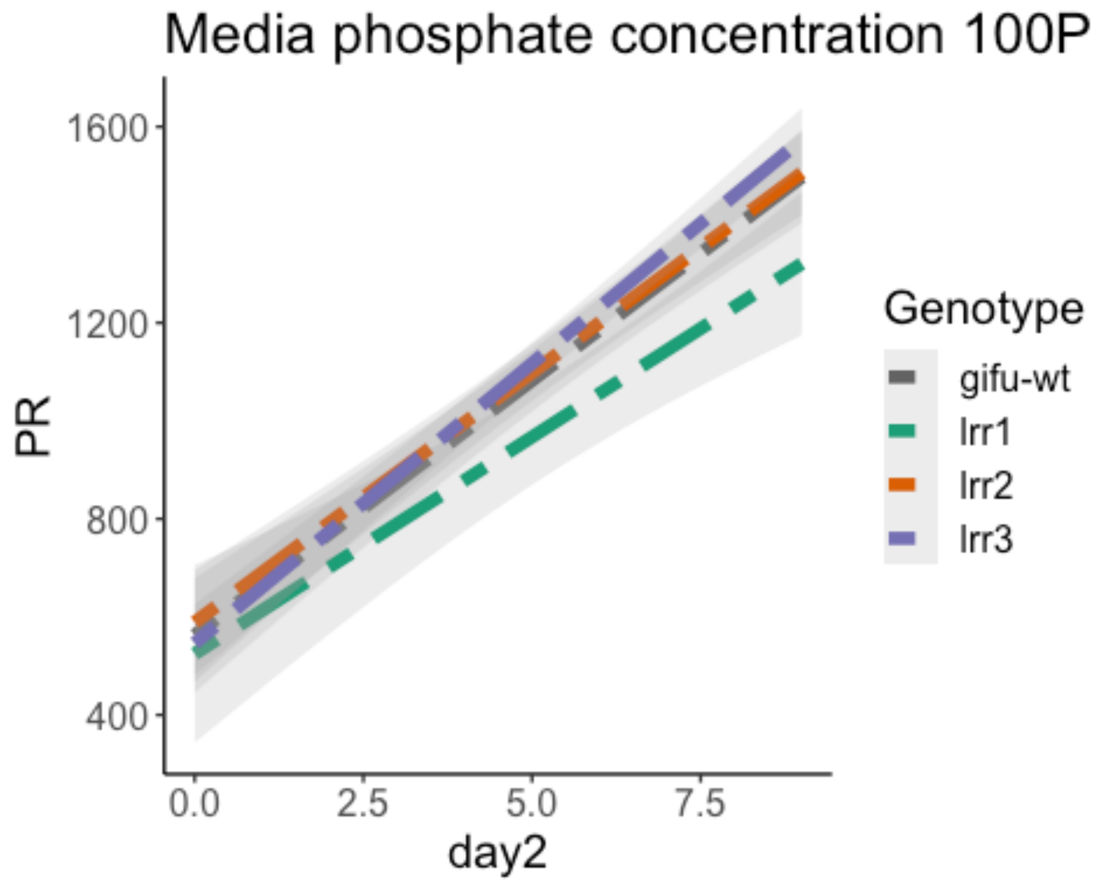
Media phosphate concentration 100P
1600
1200
800
400
PR
0.0
2.5
5.0
7.5
day2
Genotype
gifu-wt
lrr1
lrr2
lrr3

```
##
## $`750P`
```

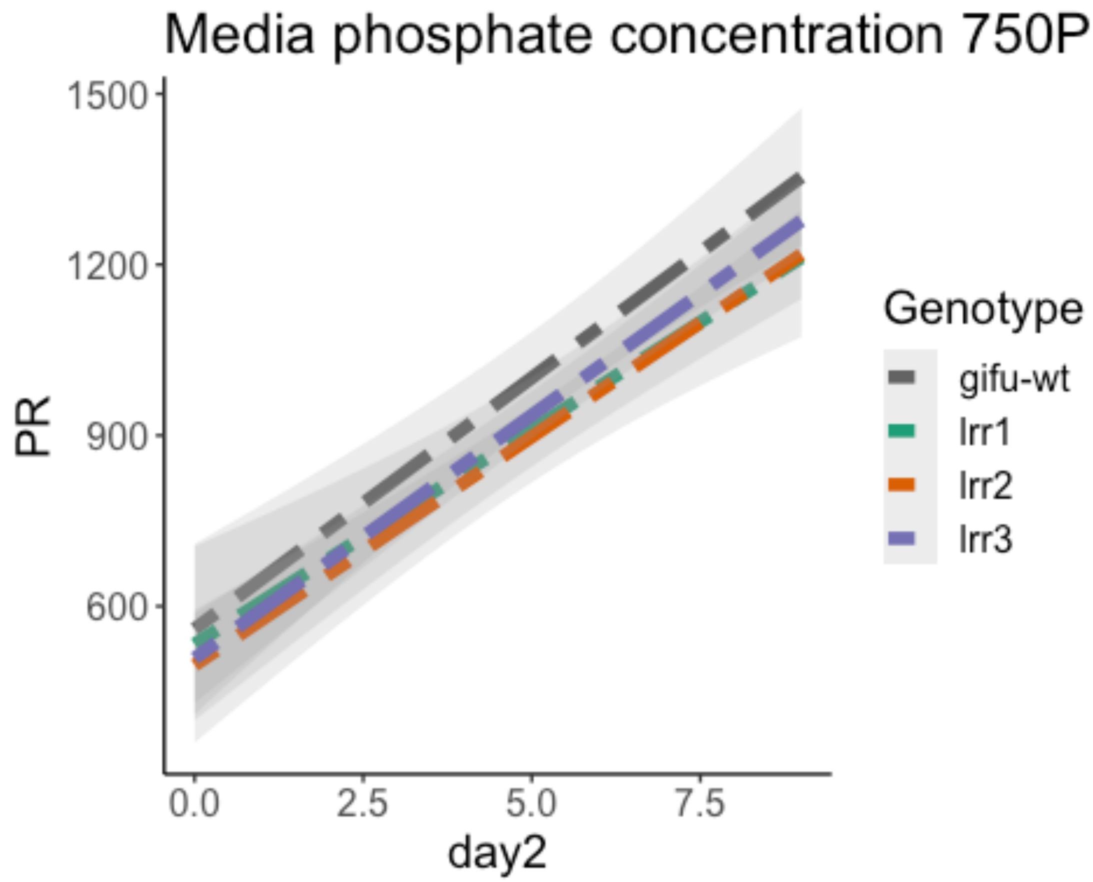


```
ggplot(data,aes(x=day2, y=PR, colour=Genotype)) +
              scale_colour_manual(values=c( "#636363","#1b9e77",
"#d95f02",  "#7570b3")) +
              #geom_point(alpha=0.5) +
              stat_smooth(method ="lm", se=TRUE, alpha=0.2, size = 2,
linetype = 6) +
              theme_classic()  +
              theme(text = element_text(size=15)) +
              labs(x = "Time (d)", y = "Primary root length (px)",
title = "LRR-RK root growth over phosphate concentration") +
              facet_grid(~Treat)
```

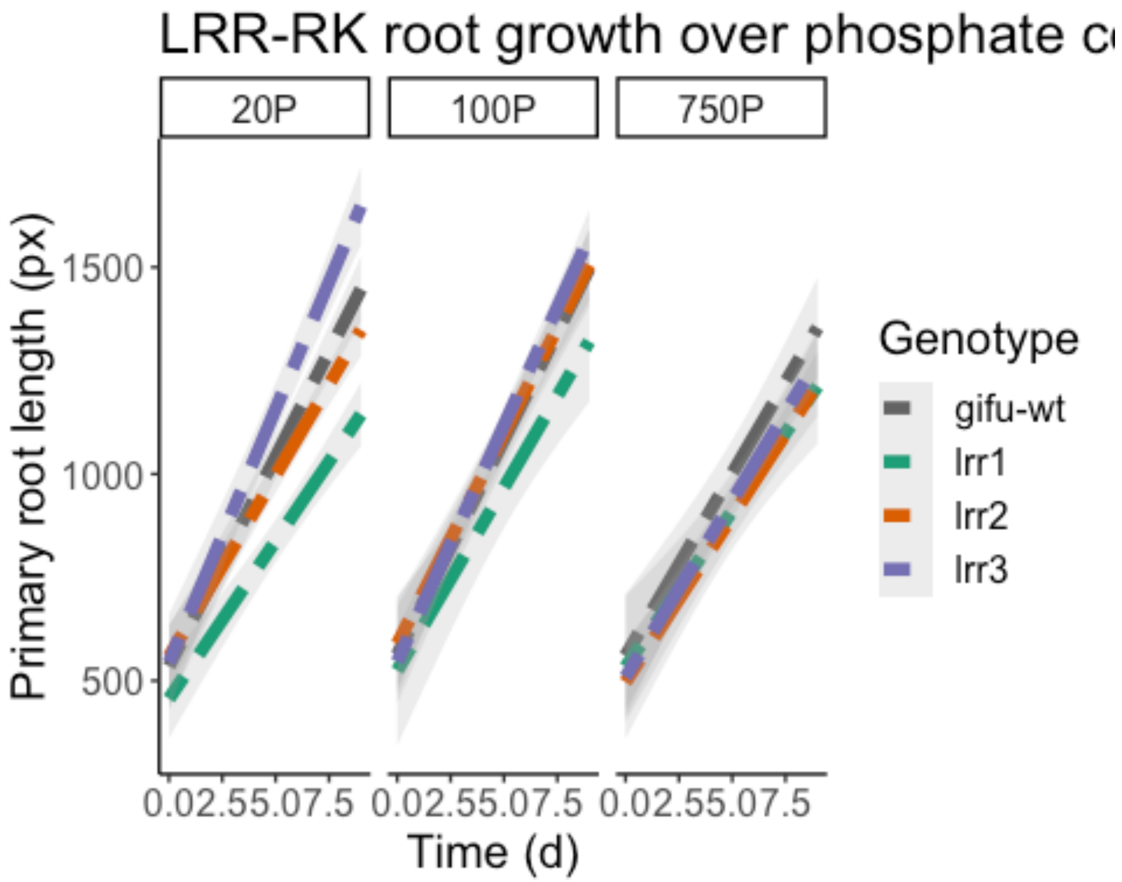

LRR-RK root growth over phosphate c
20P
100P
750P
Primary root length (px)
1500
1000
500
0.02.55.07.5
0.02.55.07.5
0.02.55.07.5
Time (d)
Genotype
gifu-wt
lrr1
lrr2
lrr3
